# High-Throughput Automated Olfactory Phenotyping of Group-Housed Mice

**DOI:** 10.3389/fnbeh.2019.00267

**Published:** 2019-12-17

**Authors:** Janine K. Reinert, Andreas T. Schaefer, Thomas Kuner

**Affiliations:** ^1^Institute of Anatomy and Cell Biology, Heidelberg University, Heidelberg, Germany; ^2^Neurophysiology of Behaviour Laboratory, The Francis Crick Institute, London, United Kingdom; ^3^Department of Neuroscience, Physiology and Pharmacology, University College London, London, United Kingdom

**Keywords:** olfaction, operant conditioning, automated, behavior assay, olfactory testing

## Abstract

Behavioral phenotyping of mice is often compromised by manual interventions of the experimenter and limited throughput. Here, we describe a fully automated behavior setup that allows for quantitative analysis of mouse olfaction with minimized experimenter involvement. Mice are group-housed and tagged with unique RFID chips. They can freely initiate trials and are automatically trained on a go/no-go task, learning to distinguish a rewarded from an unrewarded odor. Further, odor discrimination tasks and detailed training aspects can be set for each animal individually for automated execution without direct experimenter intervention. The procedure described here, from initial RFID implantation to discrimination of complex odor mixtures at high accuracy, can be completed within <2 months with cohorts of up to 25 male mice. Apart from the presentation of monomolecular odors, the setup can generate arbitrary mixtures and dilutions from any set of odors to create complex stimuli, enabling demanding behavioral analyses at high-throughput.

## Introduction

So far, behavioral analysis of mice has mostly relied on the manual characterization of individually housed animals (Buccafusco, 2008; Wahlsten, 2010). In addition to being highly time and labor intensive, manual tests are prone to experimenter-induced variations due to extensive human interaction (Wahlsten, 2001; Balcombe et al., 2004; Lewejohann et al., 2006; Sorge et al., 2014). Recently, however, commercial as well as non-commercial automated systems have become available, clearly demonstrating the trend toward more standardized approaches (Jhuang et al., 2010; Patel et al., 2014; Remmelink et al., 2015; Samson et al., 2015; Murphy et al., 2016; Nguyen et al., 2016; Aoki et al., 2017; Maor et al., 2018). Most of the commercially available setups [e.g., IntelliCage (TSE Systems), PhenoTyper (Noldus Information Technology), and ColonyRack (PhenoSys GmbH)] have been developed to examine general behavioral parameters, yet are difficult to adapt for additional custom behavioral tasks. Furthermore, most of these setups continue to require manual placement of mice in the device for limited time periods and mice are otherwise housed individually, a condition that can significantly bias the test results (Van Loo et al., 2003, 2004; Lewejohann et al., 2006).

Olfactory discrimination, too, has mostly been studied using semi-automated behavioral setups (Bodyak and Slotnick, 1999; Uchida and Mainen, 2003; Abraham et al., 2004, 2010, 2012; Slotnick and Restrepo, 2005; Kepecs et al., 2007; Slotnick, 2007; Nunes and Kuner, 2015). These typically include a testing chamber combined with a single olfactometer containing a limited number (usually 6–8) of individually addressable odor reservoirs. While each discrimination experiment is carried out automatically, mice need to be manually placed into the testing chamber at short intervals, thereby imposing continuous demand for labor and potentially even require shift operation by multiple experimenters to maximize capacity. While the number of conditioning chambers can be increased, this approach can lead to an even higher workload as potential variations between the different setups and olfactometers need to be controlled for. Last but not least, the individual training sessions are externally imposed on the animals and hence might not coincide with a native activity phase, limiting the overall number of trials attainable.

Recent work has shown that simultaneous behavior testing of large cohorts of mice can be achieved by housing a group of up to 14 mice in a home cage with free access to a behavioral testing area (“AutonoMouse”) (Erskine et al., 2019). Such a design can house two cohorts of mice (i.e., genetically modified mice and their littermate controls) for simultaneous behavior testing using a single olfactometer, thereby reducing potential sources of variation. As animals are able to freely access the testing area whenever they are motivated to obtain a reward, this setup produces a large number of trials performed by highly incentivized animals (Erskine et al., 2019).

**FIGURE 1:**
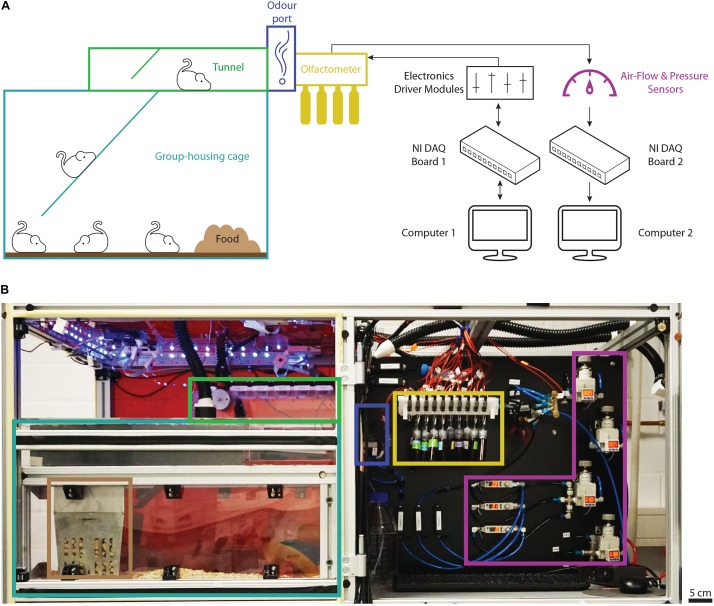
Overview of the automated behavior setup. **(A)** Schematic representation of the main components of the setup and their connections. The group-housing cage (light blue) is connected to the tunnel (green) via a ramp and provides the animals with food (brown) *ad libitum*. The odor port (dark blue) forms the end of the tunnel. Odors are presented from the custom olfactometer supplied with odor reservoirs (yellow) while the air flow is controlled and monitored by air-flow and pressure sensors (purple). Black arrows show the directionality of the main electronic connections to the data-acquisition and driver modules (black). **(B)** Photograph of the top half of the behavior setup with the main components highlighted as described in panel **(A)**. Note that the front door shielding the housing area from the outside has been removed to provide a better overview. Computers and electronics driver modules are located in the bottom half of the setup not depicted in the photograph.

Here, we describe our adaptation of the “AutonoMouse” concept, differing in the design of certain vital components as well as the software used to control it (further detailed in the section “Materials and Methods”). We provide a detailed step-by-step protocol to build the setup and to carry out odor discrimination experiments. The automated behavior setup allows for operant olfactory conditioning of a large group of mice over long time periods and complex odor discrimination paradigms without human interference. It consists of (1) the group housing cage, (2) a tunnel leading toward the odor port, (3) the odor port, (4) the olfactometer, as well as (5) computers and electronic modules driving the individual parts of the setup and acquiring sensor data (Figure 1). Due to the modular and fully customizable nature, this setup can serve as the platform for automated analysis of mouse behavior.

## Materials and Methods

### Animals

Male mice aged at least 3 weeks of any genetic background can be used. Exemplary results shown in Figures 5–7 were obtained using a total of 21 male wild-type C57BL/6N mice aged 3 weeks upon RFID implantation. Aggregate data shown in Figures 5, 8 were obtained using wild-type C57BL/6N mice (total of 35) as well as nestin^CreERT2+/–^/ROSA26^loxP(rtTA)–flox+/+^/tet-bi^4D– RFP–^ (total of 21) and nestin^CreERT2+/–^/ROSA26^loxP(rtTA)–flox +/+^/tet-bi^4D–RFP+^ (total of 17) mice. All experiments described in this protocol were conducted in accordance with the German animal welfare guidelines and approved by the responsible authority (Regierungspräsidium Karlsruhe, Germany; experimental license number 35-9185.81/G-61/15).

### Animal Housing

During post-operative recovery (7–10 days) animals were housed in groups of two to three in a non-inverted day–night cycle (dark phase: 6 PM till 6 AM; light phase: 6 AM till 6 PM) with *ad libitum* access to food (LasVendi, #Rod16) and water. Upon transfer to the automated behavior setup, animals were group-housed in the setup with *ad libitum* access to food while water (Danone Waters Germany, “Volvic Natural Mineral Water”) was only available through the behavior training paradigm. Animals were housed in a non-inverted day–night cycle (dark phase: 6 PM till 6 AM; light phase: 6 AM till 6 PM) and the lighting inside the behavior setup was controlled using a custom made LED lighting system (Adafruit, #285 and Conrad, #616020-62). Animals were provided with standard rodent bedding and nesting material (Abedd, #LTE-E-001 and #LTE-E-002 and #NBG-E-012) as well as enrichment (Plexx, #13100 and #13150; Tecniplast, #“Mouse House”).

### Implantation of RFID Chips

Mice were anesthetized by intraperitoneal injection of the anesthesia mixture [0.715 mg/kg body weight Medetomidine (Alvetra, #401295.00.00); 9.3 mg/kg body weight Midazolam (Hameln Pharma Plus, #47046.02.00); and 0.24 mg/kg body weight Fentanyl (Janssen, #6762282.01.00)]. Analgesia was provided by subcutaneous injection of Carprofen (Norbrook, #401182.00.00) at 5 mg/kg body weight. Anesthesia depth was confirmed by the absence of retraction reflexes upon toe pinching and body temperature was maintained using a feedback-regulated temperature control system (Stoelting, #50300) set at 33–35°C. The eyes were moisturized using eye ointment (Bepanthen, #“Eye and nose ointment”) and a small patch of skin on the lower back (approximately 1 × 1 cm) was shaved. The surgical field was disinfected using 70% ethanol and the skin opened through a small incision. The RFID injector (EURO ID, #IID100) was loaded with a sterile injection needle containing a single RFID chip (EURO ID, #ID100). The injection needle was inserted into the incision and pushed parallel to the spinal cord toward the neck of the animal. The RFID was released at the fold of the neck using the plunger of the injector and the injection needle was subsequently removed. The skin was sutured using surgical sutures (Braun, #0936022) which were additionally secured using skin glue (Braun, #1050052). Anesthesia was antagonized via intraperitoneal injection of the antidote mixture [1.898 mg/kg body weight Atipamezol (Prodivet, #401860.00.00); 0.506 mg/kg body weight Flumazenil (Fresenius Kabi, #63156.00.00); and 0.304 mg/kg body weight Naloxon (Inresa, #32029.00.00)]. During the initial post-surgery time, animals were given Carprofen (Norbrook, #401182.00.00) at 5 mg/kg body weight to alleviate any pain.

### Setup

A detailed parts list and 3D CAD plans are located in the accompanying online repository: https://github.com/AutomatedOlfactoryBehaviour/Beast.

#### Behavior Setup

The behavior setup consisted of a setup frame made from slotted aluminum profiles (Mesa Bammental, #1.11.040040.43S-AA4AA4) which held the group housing cage, the cage lid assembly, the olfactometer, as well as all pneumatics and electronic driver modules (Figure 1). Power for the electronic driver modules was provided using a NIM crate (Sigmann Elektronik, #3000750).

The group housing cage was constructed from slotted aluminum profiles and clear, 3 mm thick macrolon plates and measured approximately 20 × 62 × 58 cm (height × length × width). The cage lid assembly and the tunnel leading toward the odor port were custom designed and made from clear, 10 mm thick PVC plates. The tunnel was equipped with a door controlled by a rotary magnet (Magnet Schultz, #GDRX-050-X20-A01), driven by custom-made electronics (Rotary Magnet Controller; see Part 4 of the Supplementary Material). The door was not used during automated training of the animals but activated manually to block animals from entering the tunnel during maintenance or cleaning of the odor port. Additionally, the area immediately in front of the odor port contained a digital scale (Kern, #PCB 200-2) to allow for optional automated weight measurements.

#### Odor Port

**FIGURE 2:**
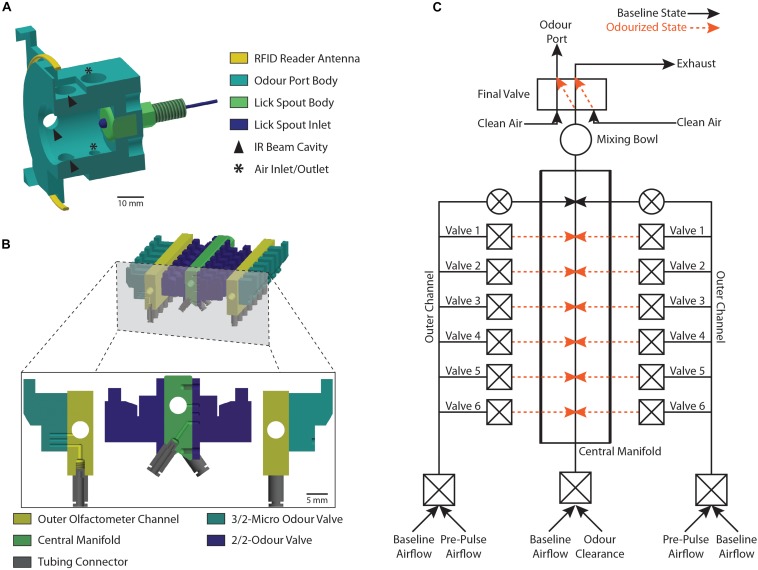
Schematic representation of the odor port and the olfactometer. **(A)** Cross-section view of the 3D printed odor port (light blue) showing the location of the RFID reader antenna (yellow) and the lick spout (dark blue and green). Arrows denote the cavities housing the IR beam LEDs and detectors while asterisks denote the position of the air-flow inlet and outlet. **(B)** Cross-section view of the olfactometer showing the location of the central manifold (green) and the two outer channels (yellow). The odor reservoirs are connected to the individual olfactometer parts using tubing connectors (gray) and are gated by two odor valves (light and dark blue) per reservoir. **(C)** Schematic representation of the olfactometer as shown in **(B)** highlighting the individual air-flow pathways. Each Valve ID (Valve 1–Valve 6) corresponds to the pair of valves gating a particular odor reservoir. Black lines show the baseline air-flow pathway when no odor is applied while orange lines represent the air-flow pathway during application of an odor. Constant flow of clean air is maintained through normally open valves (round valves) while odor application is gated through normally closed valves (square valves).

The odor port was a custom-designed 3D printed nose poke system, containing opposing air in- and outlets to create a vertical airflow within the port (Figure 2A). Nose pokes were registered using IR beams and detectors (Sigmann Elektronik, #3001046 and #3001047) while lick responses were measured using an electrical lick sensor (Sigmann Elektronik, #3001049 and #3001048). Contrary to most other published designs, the antenna (EURO ID, # EUR-3120-3m) for the RFID reader (EURO ID, # LID-665-Multi, # LID-665BP, and # CLK-665/650) was positioned around the odor port and not along the tunnel leading toward the port (Schaefer and Claridge-Chang, 2012; Schneider et al., 2012; König et al., 2015; Rivalan et al., 2017). This location ensured accurate and reliable identification of the animal currently sampling at the odor port.

#### Olfactometer

Odor stimuli were delivered using a custom made dual-channel olfactometer (see Part 1 of the Supplementary Material) with both channels providing independent input to a common central manifold (Figure 2B). Odor reservoirs were made from 20 ml brown glass bottles (Roth, #LC50.1) and custom-made silicone bottle caps (Roth, #EE02.1; Menzel Modellbau, #TC1603; Conrad, #886541-62; SMC, #TIUB01C-20; see Part 1 of the Supplementary Material). Each odor reservoir was connected to the respective olfactometer channel using tubing connectors and gated by two micro solenoid valves (Asco, #SCS067A028 and #18801088), one regulating influx of clean air and the other controlling the output of odorized air into a central manifold (Figure 2C). For odor presentation, a reservoir containing the desired odor was randomly selected and a short (120 ms) high-pressure “pre-pulse” air pulse was applied by diverting clean air through the diversion solenoid valve (Asco, #SCS067A108 and #36100040). Lastly, the odor pulse was presented to the animal by switching to the odorized air stream at the five-way final valve (SMC, #VK3120-5D-M5). All valves were chosen for their low internal volume and fast switching properties. Additionally, in contrast to commonly used cheaper pinch valves, these valves operate by diverting airstreams directly instead of clamping odor tubing, thus these valves do not lead to the gradual fatigue of connected tubing and are considerably less noisy (Bodyak and Slotnick, 1999).

#### Air Supply Control

Due to the high amount or air needed, regular pressurized air was used. Input air was cleaned using oil filters (SMC, #AMH250C-F02D AMH-EL250 and #AME250C-F02 AME-EL250) as well as an odor filter (SMC, #AMH250C-F02 AMF-EL250) before entering the olfactometer. Air pressure was adjusted using pressure reducers (SMC, #IR2000-F02 and #ISE30A-01-B-ML) while the speed was controlled using flow and mass sensors (SensorTechnics, #WTAL005DU, #WTAL010DUP, and #WBAM200DuHo; SMC, #PFM710S-F01-E). Air flow during odor presentation was controlled using custom-made air-pressure controllers (Sigmann Elektronik, #3000701 and #3000794). Typically, we used an air-flow of 2 l/min.

#### Water Delivery System

The water reward provided to the animal for correctly identified S+ trials was delivered using a micro annular gear pump (Harton, #11010103) controlled by a custom-made controller (Harton, #66020101 and #92000935; Part 2 of the Supplementary Material). Contrary to gravimetric systems, this active pump guarantees the precise delivery of the intended water amount irrespective of the fluid level of the water reservoir. Since this pump is also capable of accurately delivering fluids other than water, like saccharine or ethanol solutions, this system is compatible with many alternative training paradigms.

### Automated Behavior Training Paradigm

**FIGURE 3:**
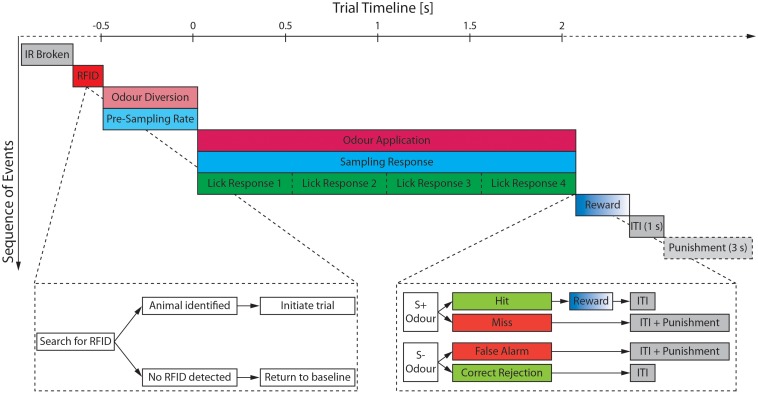
Schematic of the trial structure and the most relevant monitored parameters. After a break of the IR beam has been detected (gray), the search for an RFID signal is started (red). Once an animal has been detected and identified the trial is initiated starting with an initial 500 ms period during which the airstream is diverted through the odor reservoirs (light magenta) and the pre-sampling rate (light cyan) is measured. If the pre-sampling rate surpasses the defined threshold, the final valve opens and the odor is applied (magenta) for maximally 2 s unless the animal retracts its head from the odor port before the 2 s have elapsed. During the odor application both the sampling rate (cyan) as well as the lick response (green) of the animal are monitored. The water reward (blue) is only delivered upon correct identification of a S+ odor (=Hit) followed by a mandatory inter-trial interval (ITI; gray) of 1 s. Any false response to an odor (=Miss or False Alarm) incurs an additional time penalty of 3 s increasing the ITI to 4 s. Time is shown relative to opening of the final valve.

The training protocol and trial structure was based on the manual training paradigm described by Abraham et al. (2004) with minor modifications to allow for automated training. An animal could initiate a trial by inserting its head into the odor port and thus breaking the IR beams (Figure 3). However, a trial was only started if the RFID chip was identified. If an incomplete or no RFID was detected (i.e., because the animal directly withdrew its head from the port), the setup reverted to the baseline state and the trial was aborted (Figure 3). Automated training was divided into acquisition of the go/no-go paradigm (“pre-training”) and discrimination of two olfactory stimuli.

#### Pre-training

During the pre-training, animals were gradually trained on the behavior paradigm by successive addition of new conditions for receiving a water reward (Table 1). For example, at the start of the pre-training (phase 1) any insertion of the head into the odor port resulted in a water reward. Throughout the course of the pre-training, the delay between initiation of a trial and dispersion of the water reward was gradually increased (i.e., to 500 ms at the end of phase 3).

**TABLE 1:** Parameters used during the pre-training phase of automated behavior training.

**Pre-training phase**	**Pre-sampling threshold (minimal sampling rate before FV opening)**	**Lick criterion (number of 500 ms blocks until water reward is given)**	**Delay until water reward (ms)**	**Minimal trial number**	**Water reward (μl)**	**Inter-trial interval (s)**
1	0	0	0	50	20	0–0.5
2	0	0	0	50	20–10.5	0.5–3.9
3	12.5%	0	21–500	100	10	1
4	25%	0	500–1980	100	10	1
5	50%	1	2000	100	10	1
6	75%	2	2000	100	10	1
7	87.5%	3	2000	100	15	1

Mice could only progress from one training stage to the next upon completing a certain minimal number of trials (50 or 100; Table 1) and performing with at least 80% accuracy within the last 20 trials of this stage. If the performance criterion was not met, the mice remained in the stage for more trials until the performance criterion was reached. The last phase of the pre-training was identical to the trial structure during odor discrimination (Figure 3), the only exception being that mice were presented not with odorized air but clean air only.

#### Odor Discrimination

After completion of the final pre-training phase, animals could progress to the actual odor discrimination. During this, animals were presented with either of two odors with one being rewarded (S+ odor) and the other one not (S− odor; Figure 3). A water reward was given upon correct identification of the S+ odor (“Hit”) while the mandatory inter-trial interval was increased by 3 s upon incorrect identification of an S+ odor (“Miss”) or S− odor (“False Alarm”). During application of an odor, the sampling behavior at the odor port as well as the lick response were monitored. Animals would gradually cease licking for the unrewarded odor and retracted their head while they would leave their head in the odor port and continue to lick for the rewarded odor to receive the water reward.

The sequence of trials was pseudo-randomized in blocks of 1000 trials with (i) no more than three consecutive rewarded or unrewarded trials and (ii) approximately 50 rewarded and unrewarded trials within each 100 trial bin. Every sequence of 1000 trials could only be repeated after completion of the full sequence of 1000 trials. Additionally, designation of the odors as rewarded and unrewarded was counterbalanced within each treatment group to ensure that no intrinsic preferences for any odor could confound the results.

### Time Requirements

The behavior setup itself was built and wired in <1 week by a single experimenter, yet we recommend at least two people for building the frame of the setup. Validation of the olfactometer required roughly 2 h, but 1 day should be allotted for the initial validation in case modifications need to be made. The RFID surgery took roughly 10–20 min per animal depending on the surgery skills of the experimenter and mice were allowed to recover for at least 5 days before the start of the behavior experiment. This recovery time can be expanded to suit individual experimental plans.

Mice typically required 5–7 days to finish the pre-training phase while quick learners could complete the pre-training in as little as 3 days. While highly accurate odor discrimination alone was typically achieved in <1 week per odor pair, additional high-performance trials are needed to accurately determine the discrimination time (DT). Hence, training a set of 20–25 mice on a full assay (two pairs of pure odors and one pair of odor mixtures) typically took 20–30 days.

The animals were remotely monitored using Raspberry Pi-based video surveillance (see Part 3 of the Supplementary Material) reducing physical experimenter presence to check-ups as mandated by local animal welfare laws (i.e., 5–15 min/day).

Cleaning of the cage took roughly 1 h including disassembly and reassembly of the tunnel. We recommend cleaning once a week for a full set of 20–25 animals; however, the intervals could be increased if smaller group sizes are used and the bedding is thus less soiled.

### Odors

All odors (Table 2) used during the behavioral experiments and during validation of the olfactometer were diluted in mineral oil (Sigma, #69794) to a final concentration of 1% (v/v). For binary mixtures (“AA/EB Mix”), animals were tasked to discriminate a mixture containing 0.6% amyl acetate and 0.4% ethyl butyrate from a mixture containing 0.4% amyl acetate and 0.6% ethyl butyrate. All odors were presented at a speed of 2 l/min and odor reservoirs were periodically (ca. every 7–14 days) replaced.

**TABLE 2:** List of odors used for automated olfactory phenotyping and validation of the olfactometer.

**Odor**	**Abbreviation**	**Company**	**Catalog number**
Cineol	Cin	Sigma	27395
Eugenol	Eu	Fluka	46100
Amyl acetate	AA	Sigma	109584
Ethyl butyrate	EB	Sigma	E15701
2-Butanone	–	Sigma	34861

### Data Acquisition

The setup was controlled and the data acquired using a standard desktop computer [Minimal Requirements: Windows 7 (or later); min. 4 GB RAM; 1 PCI-slot; recommended: Windows 7; 8 GB RAM; 1 PCI-slot; SSD (recommended 120 GB) for operating system and separate HDD (recommended 1 TB) for temporary local data storage] with a PCI I/O-Device (National Instruments, #779068-01) and a BNC Connector Block (National Instruments, #779556-01). Data were analyzed using custom written algorithms based on Igor 6 (Wavemetrics, United States) utilizing the Igor XOP Toolkit and the NIDAQ Tools MX package. All code available from the accompanying online repository: https://github.com/AutomatedOlfactoryBehaviour/Beast.

### Differences From the “AutonoMouse” Variant

As our implementation was developed based on an earlier prototype of the “AutonoMouse” system, they share a similar design, yet some differences between the two systems should be highlighted: firstly, the setups use different software to run and analyze the behavioral data. While our software is based on a proprietary computing environment, it is directly pre-configured to run all habituation and odor discrimination tasks shown in this paper. Since it also includes built-in analysis options as well as tabular and graphical output options, it provides a self-contained, “ready-to-go” experimental platform, making it especially suited for novel users. Secondly, we incorporated a modular cage lid that fully encloses the housing cage yet remains physically independent from the cage. On the one hand, this system turns the housing cage and the lid into one unified and self-contained assembly. Still, parts or even the entire cage lid can easily be removed or switched without the need to disturb the animals in the housing cage. On the other hand, this design also allows for group-housing of experimental cohorts in detached home cages prior to the start of any behavior experiments, again removing the need to disturb the animals inside the housing cage when transferring them to the setup. Thirdly, we designed a modular and customizable 3D-printed nose poke (Figure 2A) that is fixed to the tunnel by a simple sliding mechanism. This system allows for easy maintenance as well as greatly increased flexibility for switching odor port designs. Users can, for example, freely adjust the number and position of the IR beams, add sniff sensors or switch from a single nose-poke to an alternate choice system without the need to dismantle the behavior testing area. Lastly, we revised some more general aspects of the behavior apparatus (i.e., use of a bedding chute compared to a large and heavy bedding drawer, incorporation of active ventilation of the housing cage and tunnel compared to passive ventilation through the floor of the behavior testing area, inclusion of low-cost surveillance cameras, etc.).

### Statistical Analysis

Statistical analysis was performed using Igor 6 (Wavemetrics, United States) and Prism 6.0 (Graphpad, United States) (^∗^*p* < 0.05; ^∗∗^*p* < 0.001; ^∗∗∗^*p* < 0.001). Potential outliers were identified using the ROUT method as detailed by Brown and Motulsky (2006) and as implemented in Prism 6.0 (Graphpad, United States). Graphical representations were generated using Prism 6.0 (Graphpad, United States) and Adobe Illustrator CS4 (Adobe Systems, United States). Unless otherwise noted data are shown as mean ± SEM.

## Results

### Odor Presentation

**FIGURE 4:**
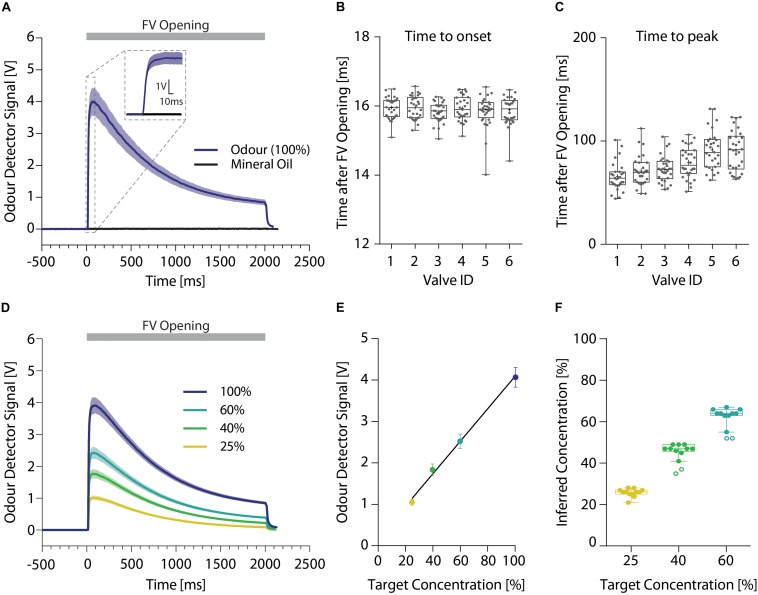
High resolution temporal analysis of different odor stimuli. **(A)** Representative signal obtained using an ionization detector for presentations of 2-butanone (dark blue; *n* = 12) or mineral oil (black; *n* = 10) without addition of any odors. Inset shows 20 ms prior and 100 ms after opening of the final valve in more detail. **(B)** Time until odor onset (defined as 10% of maximal odor concentration) and **(C)** peak odor concentration. **(D)** Representative odor pulse shapes for application of undiluted 2-butanone (dark blue) and applications of 60 (light blue), 40 (green), or 25% (yellow) 2-butanone dilutions directly generated using the olfactometer (*n* = 12 per dilution). **(E)** Correlation between the detected ionization intensity and the target odor dilution (*n* = 12 per dilution) (line denotes Pearson correlation coefficient). **(F)** Relationship of the intended target odor concentration and the inferred odor concentrations, extrapolated from the measured odor detector signal (*n* = 12 per dilution). **(A)**/**(D)**: Time relative to opening of the final valve. Line and shaded areas denote mean ± SEM. **(B)**/**(C)**/**(F)**: Whiskers show 1.5 interquartile range, line denotes median while cross denotes mean and circles represent potential outliers as identified by ROUT test.

To ensure reliable and precise delivery of the odor stimuli, we used a dual channel olfactometer capable of regulating up to nine individual odor reservoirs per channel (Figures 2B,C). Both olfactometer channels were connected to an individually regulated air supply, allowing for the independent application of air at freely adjustable air-pressures per channel. Odorized air from both channels was combined in the central manifold generating an airstream containing the desired concentrations or mixtures of the odors. Using this olfactometer at an air-flow rate of 2 l/min, a PID sensor placed within the odor stream detected the odor onset consistently 16 ms (15.91 ± 0.02 ms) after final valve opening (Figures 4A,B), regardless of the distance of the odor reservoir from the final valve [one-way ANOVA, *F*(5,167) = 0.6601; *p* = 0.654]. The peak odor concentration was on average reached in 80 ms (78.30 ± 3.98 ms; Figure 4C) yet there was a significant increase with increasing distance to the final valve [one-way ANOVA, *F*(5,172) = 13.33; *p* < 0.0001]. Despite this correlation, the maximal difference between the valves was <30 ms (valve ID 1: 65.38 ± 2.55 ms; valve ID 5: 91.38 ± 3.48 ms). Both of these times could be further reduced by increasing the air flow speed, enabling odor presentations suited for many different test paradigms. Lastly, this dual-channel design allowed for the generation of arbitrary odor dilutions and mixtures directly from the pure odor reservoirs (Figure 4D). By applying a range of different dilutions, we found a very tight correlation between the intended odor concentration and the concentrations measured using the ionization detectors (Figures 4E,F; Pearson correlation coefficient; *r* = 0.99, *p* = 0.002), greatly increasing flexibility of this olfactometer compared to single channel olfactometers.

### Acquisition of the Go/No-Go Task

**FIGURE 5:**
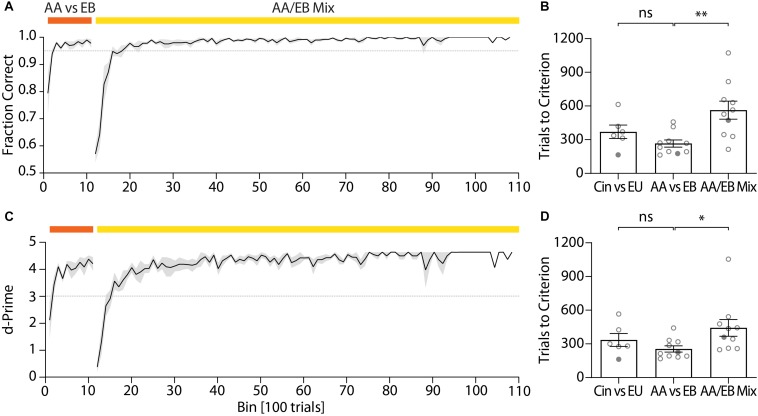
Representative performance during subsequent discrimination of pure odors and the binary mixtures of these two odors. **(A)** Discrimination accuracy throughout training for an exemplary cohort (*n* = 5) and **(B)** trials to reach criterion (0.95) for all tested cohorts (*n*_cohorts_ = 10). **(C)** d-Prime scores for the exemplary cohort shown in Graph **(A)** and **(D)** trials to reach d-Prime criterion (3) for all tested cohorts (*n*_cohorts_ = 10). **(A)**/**(C)**: Black line and shaded area denote mean ± SEM (*n* = 5). Dashed lines denote threshold (fraction correct: 0.95; d-Prime: 3). **(B)**/**(D)**: Circles denote individual test cohorts while filled circles highlight test cohort shown in **(A)**/**(C)**. ^∗^*p* < 0.05; ^∗∗^*p* < 0.001.

After the pre-training, mice typically reached high performance for the discrimination of two odors within a few hundred trials per odor pair (Figure 5A). An initial odor pair was usually acquired within less than 400 trials (cineol vs. eugenol: 370.1 ± 59.98 trials; Figure 5B). As the first odor pair also involved learning of the actual go/no-go paradigm, high performance for a second odor pair was usually reached faster (amyl acetate vs. ethyl butyrate: 265.9 ± 31.63 trials; paired *t*-test, *p* = 0.058; Figure 5B). For highly similar odors (such as binary mixtures of two odors), mice again required more trials to reach high performance due to the difficulty of the task (binary mixture of amyl acetate and ethyl butyrate: 563.3 ± 79.70 trials; paired *t*-test, *p* = 0.0055; Figure 5B) even if they previously successfully learned to distinguish the underlying pure odors. Still, for a 60/40% mixture of amyl acetate and ethyl butyrate a fraction of correct trials of at least 0.95 was typically reached after ca. 600 trials and could be maintained over thousands of trials (Figure 5A).

The quick acquisition of the discrimination paradigm was also evident from the d-prime scores, a measure of discriminability of two stimuli (Nevin, 1969), which remained high even over long training periods (Figure 5C). Mice typically reached a d-prime score of at least 3 within 300–400 trials for the initial odor pair (cineol vs. eugenol: 335.6 ± 57.23 trials; Figure 5D). Analogous to the performance values, high d-Prime scores for the second odor pair were attained faster (amyl acetate vs. ethyl butyrate: 255.3 ± 27.66 trials; paired *t*-test, *p* = 0.2387; Figure 5D) yet required more trials for complex odor mixtures (binary mixture of amyl acetate and ethyl butyrate: 442.7 ± 75.04 trials; paired *t*-test, *p* = 0.0435; Figure 5D).

### Effect of Group Size on Number Trials and Discrimination Accuracy

**FIGURE 6:**
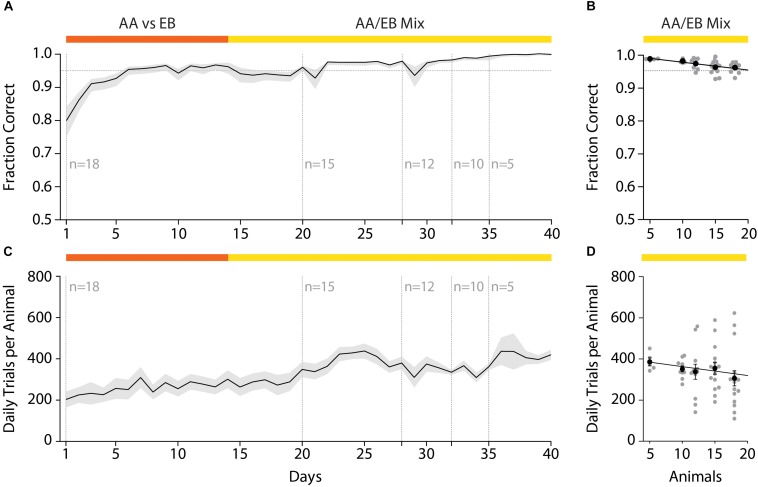
Effect of varying group sizes on daily discrimination accuracy and number of trials. **(A)** Discrimination accuracy over time with random removal of animals from the original test group. **(B)** Discrimination accuracy as negatively correlates with the number of animals in the group (Pearson correlation coefficient: *r* = –0.93; *p* = 0.022; slope = –0.49). **(C)** The number of trials over the course of the training duration with successive reduction of the group size. **(D)** The number of trials performed per animal negatively correlates with the number of animals in the group (Pearson correlation coefficient: *r* = –0.88; *p* = 0.049; slope = –5.124). **(A)**/**(C)**: Black line and shaded area denote mean ± SEM (*n* = 18–5). **(B)**/**(D)**: Line denotes best-fit of Pearson correlation, black dots represent group average (±SEM) while gray dots denote individual animals (*n* = 18–5).

After initial acquisition of the odor pair discrimination task, the discrimination accuracy remained stable even over the course of long (>1 month) training paradigms (Figure 6A). Changing the group size and composition by randomly removing animals from the initial test cohort revealed a correlation of the daily performance and the group size (Figure 6B; Pearson correlation coefficient: *r* = −0.93; *p* = 0.022; slope −0.49); however, the decrease in accuracy with increasing group size was found to be negligibly small (Fraction correct *n* = 5: 0.99; *n* = 18: 0.93) and might have been the result of continued training.

In this automated setting, mice would typically perform 200–400 trials per day which tended to increase over the course of the training paradigm (Figure 6C). This increase was to be expected, as increased accuracy due to training also reduces the “time punishment” imposed after incorrect trials, hence allowing for the initiation of trials in shorter intervals. In contrast to the daily discrimination accuracy, the number of trials performed per day increased when mice were removed from the home-cage (Figure 6D). While this effect was again found to be correlated with the number of animals in the cohort (Pearson correlation coefficient *r* = −0.88; *p* = 0.049: slope = −5.124), the increase was found to be small (average daily trials *n* = 5: 385.76 ± 19.38 trials; *n* = 18: 305.71 ± 36.75 trials) and might have been the result of increased metabolism for these growing young-adult mice. In conclusion, even large cohorts of mice could be used with minor, yet tolerable, decreases in the number of daily trials and daily discrimination accuracy.

### Effect of Circadian Rhythm

**FIGURE 7:**
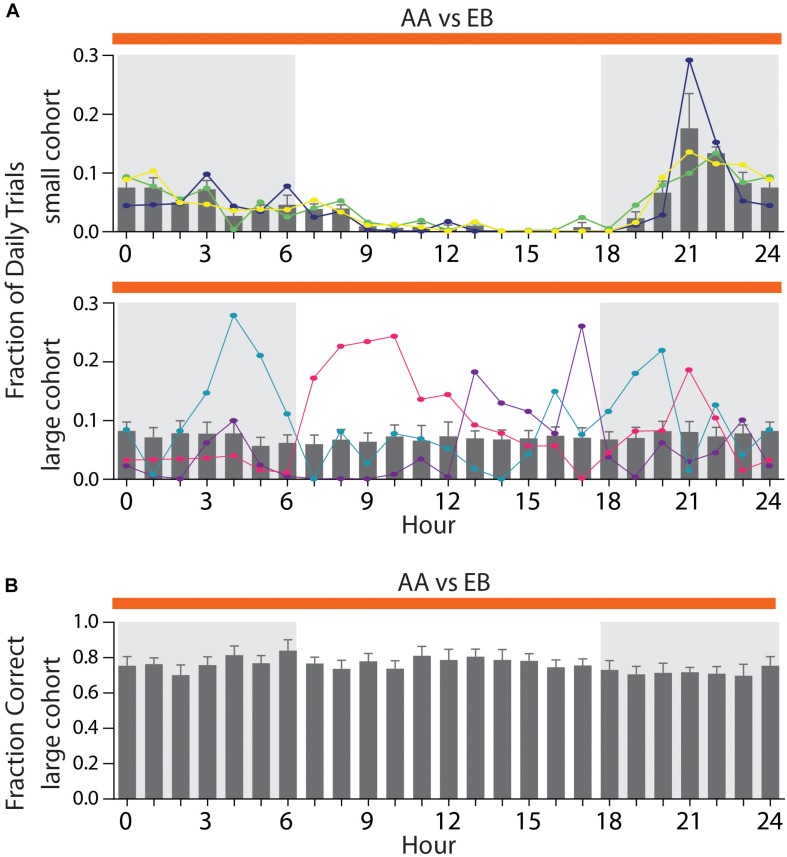
Distribution of activity throughout the day depending on cohort size. **(A)** Average fraction of daily trials for a small cohort (top; *n* = 3) compared to a large cohort (bottom; *n* = 17). Three representative animals per cohort are superimposed to highlight individual activity peaks per animal (top: green, yellow, and dark blue; bottom: light blue, pink, and purple). **(B)** Average performance per hour across all animals in a large cohort [*n* = 17; same cohort as shown in Graph **(A)**]. **(A)**/**(B)**: Shaded areas denote the dark phase of the day–night cycle (6 PM till 6 AM). Gray bars denote group averages ± SEM. Note that hour 0 and 24 are identical and duplicated for display purposes only.

In smaller cohorts, all mice would usually perform the majority of their daily trials during the dark phase (Figure 7A, top), with the activity peaking in the fourth hour of the night phase (maximal activity: 17.6 ± 5.9%). In larger cohorts (Figure 7A, bottom), however, the average activity was distributed throughout the day with no appreciable peak in activity (mean: 7.19 ± 0.14%). This occurs since some mice will inevitably show their peak activity phase during the light phase of the day/night cycle (Figure 7A, bottom, orange trace), while others retain their peak activity within the dark-phase (Figure 7A, bottom, blue trace). We have found these preferred activity phases to remain constant even over long training periods or after switching odor pairs (data not shown). We have found no correlation between the hour of the day and the discrimination accuracy [Figure 7B, two-way ANOVA, *F*(24,384) = 0.446, *p* = 0.99], indicating that even in large groups of animals, trials of the same quality can be obtained throughout the entire day.

### Reproducibility

**FIGURE 8:**
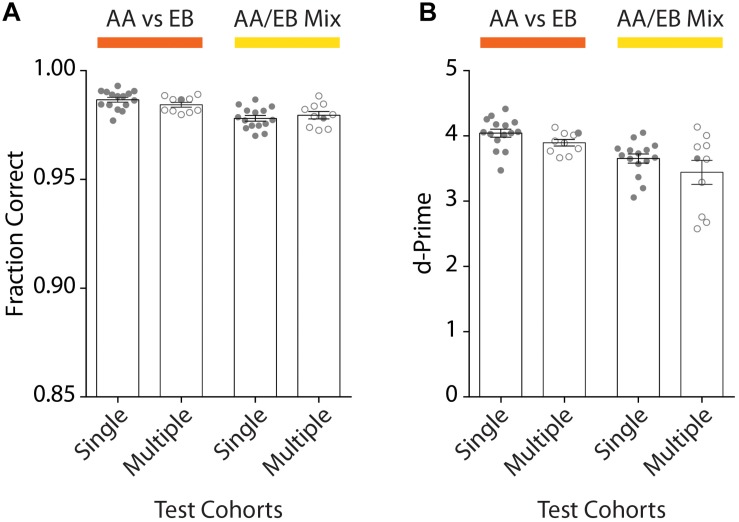
Comparison of the discrimination accuracy of one exemplary cohort of animals compared to all tested cohorts for the discrimination or pure and mixed odors. **(A)** Average discrimination accuracy after reaching the performance threshold of 95% performance for one representative cohort (“Single Test Cohort”: *n* = 15) compared to all average accuracies of all behavioral cohorts (“Multiple Test Cohort”: *n*_cohorts_ = 10; *n*_animals_ = 73; 4–15 animals per cohort). The accuracy remains above the threshold for individual animals as well as entire cohorts for the discrimination of both pure (orange; “AA vs. EB”) and mixed (yellow; “AA/EB Mix”) odors. **(B)** d-Prime scores measured across the entire training period remain well above 2 both for the representative cohort [same cohort as shown in Graph **(A)**] as well as all behavior cohorts across the individual discrimination tasks (“Single Test Cohort”: *n* = 15; “Multiple Test Cohort”: *n*_cohorts_ = 10; *n*_animals_ = 73; 4–15 animals per cohort). **(A)**/**(B)**: Bar graphs show mean ± SEM. Points in “Single Test Cohort” bars represent individual animals while circles in “Multiple Test Cohort” bars represent the average of individual genotypes per experimental run. Filled circles in “Multiple Test Cohort” bars correspond to data shown in respective “Single Test Cohort” bars.

To assess the reproducibility of our automated go/no-go conditioning paradigm, we trained a total of 73 mice (*n*_cohorts_ = 10) of different genetic backgrounds. After initial acquisition of the odor pairs, high discrimination accuracy was well above the threshold both for the animals of any given cohort (mean_Single_: 0.99 ± 0.001) as well as across all experimental runs (mean_Multiple_: 0.98 ± 0.001; Figure 8A). Similarly, the d-Prime score remained above 3 for individual cohorts (mean_Single_: 4.04 ± 0.06) and all experimental runs (mean_Multiple_: 3.90 ± 0.05; Figure 8B). For complex stimuli we observed a slight decrease in the discrimination accuracy (mean_Single_: 0.98 ± 0.001; mean_Multiple_: 0.98 ± 0.001) as well as a more pronounced drop of the d-prime scores (mean_Single_: 3.65 ± 0.07; mean_Multiple_: 3.44 ± 0.18). Still, the fraction of correct trials of at least 0.95 and a d-prime score well above 2 even for complex stimuli demonstrate the robustness of training and the versatility of the setup.

## Discussion

Using the automated behavior setup, we were able to train mice on a go/no-go odor discrimination task with animals reaching high accuracy within similar numbers of trials reported for manual setups, yet without the drawback of constant experimenter interaction with the animals (Abraham et al., 2004; Nunes and Kuner, 2015, 2018). While we did ostensibly find a link between the size of the behavior cohort and the number of trials required to reach high accuracy odor discrimination, the observed variance was again well within the range of manual setups and may reflect the typical variance of behavior testing (Abraham et al., 2004; Nunes and Kuner, 2015, 2018).

### Analysis of Olfactory Perturbations Using Automated Behavior Testing

Among other tests, we have utilized this setup to phenotype a previously uncharacterized transgenic mouse line in which adult neurogenesis could be increased in a temporally and spatially controlled manner, resulting in the increase of adult born olfactory bulb interneurons (Bragado Alonso et al., 2019). The high degree of automation with limited experimenter interference allowed for the screening of a total of 38 mice (with as many as 25 mice within a single experimental cohort) and training durations of over 3 months, spanning a large array of odor pairs and dilutions (Bragado Alonso et al., 2019). These results show that it is feasible to characterize previously untested mouse lines using this automated approach. Similarly, the “AutonoMouse” variant of this setup (see the section “Materials and Methods” for description of the differences between the variants) has recently been used to rigorously quantify the impact of graded olfactory bulb lesions on odor discrimination (Erskine et al., 2019). Taken together, the results gathered independently using the different variants clearly demonstrate the versatility and reproducibility of this approach to automated olfactory phenotyping.

### Further Applications of the Automated Training Setup

In addition to simultaneously phenotyping large cohorts of mice or testing a large variety of arbitrary odor mixtures, the setup could also be a useful tool to prepare mice for more complex experiments like *in vivo* imaging or electrophysiological recordings. As these experiments themselves can be very time consuming, the setup could be used to, for example, generate a continuous supply of pre-trained animals without the need for potentially time and labor-intensive manual training. Along these lines, the setup could also be used to identify well performing animals prior to, for example, habituating them to a head-fixed live imaging setup, reducing the risk of wasting resources on animals which may turn out to perform only very few trials or be particularly inept at a given behavior task.

Furthermore, due to the modular and non-proprietary nature, the setup can be modified to encompass additional stimuli (like visual or tactile cues) or more complex olfactory tests including sniff sensors or multiple lick ports for alternative choice tasks. Lastly, the odor port could even be adjusted to include voluntary head restraining coupled with chronic imaging or even wireless optogenetic manipulation, allowing for a myriad of experimental designs (Scott et al., 2013; Montgomery et al., 2015; Murphy et al., 2016).

## Data Availability Statement

The datasets generated for this study are available on request to the corresponding author.

## Ethics Statement

The animal study was reviewed and approved by the Regierungspräsidium Karlsruhe, Germany (experimental license number 35-9185.81/G-61/15).

## Author Contributions

AS conceived the automated behavior setup and wrote part of the hardware control code. JR wrote the analysis code, the sensor acquisition code, and integrated the code by AS, and wrote the manuscript with input from TK and AS. JR built and validated the setup, designed part of the hardware, designed and performed the experiments, analyzed the data. TK supervised the above mentioned tasks. All authors approved the final manuscript.

## Conflict of Interest

The authors declare that the research was conducted in the absence of any commercial or financial relationships that could be construed as a potential conflict of interest.
