# Supplementary material for: High-Throughput Automated Olfactory Phenotyping of Group-Housed Mice
Source: Front Behav Neurosci. 2019 Dec 17;13:267. doi: 10.3389/fnbeh.2019.00267 (PMC6927946; doi:10.3389/fnbeh.2019.00267)
Supplement: Supplementary file 1 [file Data_Sheet_1.pdf]

Supplementary Information for:

High-throughput automated olfactory phenotyping of group-housed mice

Authors

Janine K Reinert[1,2], Andreas T Schaefer[1,3,4], Thomas Kuner[1]

[1] Institute for Anatomy and Cell Biology, Heidelberg University, Heidelberg, Germany

[2] present address: Sensory and Behavioural Neuroscience Unit, Okinawa Institute of Science and Technology Graduate University, Okinawa, Japan

[3] The Francis Crick Institute, Neurophysiology of Behaviour Laboratory, London, United Kingdom

[4] Department of Neuroscience, Physiology and Pharmacology, University College London, London, United Kingdom

Corresponding authors:

Janine Reinert: janine.reinert@oist.jp

Thomas Kuner: kuner@uni-heidelberg.de

Table of Content

Table of Content i

Preface 1

Part 1 – Step by step procedures 1

- Construction of the behaviour setup 1
- Preparation of odour reservoirs 17
- Olfactometer preparation and validation 18
  - General considerations for odour application and validation of the olfactometer 18
- Animal and setup preparations 20
- Automated behaviour training 21
- Setup cleaning 22
- Troubleshooting 22

Part 2 – Detailed installation instructions 25

- Connection of pneumatic tubing 25
- Electrical Connections 26
  - Physical connection of the DAQ devices 26
    - PC 1 26
    - PC2 26
  - Wiring of the BNC Boards 26
    - BNC 1 – BNC Connectors 26
    - BNC 1 – Spring Block Terminal 26
    - BNC 2 – BNC Connectors 27
    - BNC 2 – Spring Block Terminal 28
  - Wiring and Settings of the Electronic Driver Modules 28
    - Valve Driver 28
    - Rotary Magnet Controller 28
    - IR-LED Barrier Driver 29
    - Micro Annular Gear Pump Driver 29
    - Gas Flow Regulator 1 29
    - Gas Flow Regulator 2 29
    - Air Regulator Main Block 29
- Installation of the RFID reader 29
- Installation of the video surveillance 30
  - Software 30
- Installation of temperature and humidity logging 31

Part 3 - BEAST Software 32

Introduction 32
Principle 32
Installation of Igor Pro 6 32
Recommended changes to the default parameters 32
Installation of DAQ devices 32
Hardware Referencing 33
Networking of the two PCs 33
Behaviour Software (PC 1) 33
Principle 33
Main window 33
Starting a new behaviour run 34
Naming conventions for training schedules 35
Adjusting the training schedule after initial initialization of the training 35
Quick monitoring 35
Built-in analysis options 36
Advanced options 37
Creating new training stages 37
Quick changes to training stage templates 37
Manually moving animals to specific training stages 38
Switching locations of odour reservoirs 39
Technical considerations for the calculation of discrimination times 40
Sensor Acquisition Software (PC 2) 42
Principle 42
User interface 42
Required user input 42
Data output 43
Potential modifications to conserve storage space 43
Part 4 – Schematics of custom made electronics modules 44
Supplementary Figure 30 – Rotary Magnet Controller 45
Supplementary Figure 31.1 – Gear Pump Controller Part 1 46
Supplementary Figure 31.2 – Gear Pump Controller Part 2 47
Supplementary Figure 31.3 – Gear Pump Controller Part 3 48
Supplementary Figure 32.1 – Valve Controller Part 1 49
Supplementary Figure 32.2 – Valve Controller Part 2 50
Supplementary Figure 32.3 – Valve Controller Part 3 51
References 52

Preface

The following guide specifies how to assemble and install the automated behaviour setup and the necessary components. The full bill of materials as well as the CAD schematics can be found in the accompanying online repository (https://github.com/AutomatedOlfactoryBehaviour/Beast).

Please also refer to the original publication for details.

Part 1 – Step by step procedures

The following stepwise guide is aimed especially at novel users to allow for efficient training progress. A non-exhaustive troubleshooting guide for the most common caveats can be found in Table 1.

All procedures involving animals must be conducted in accordance with any applicable local legislation, regardless of the procedures specified in this guide.

Construction of the behaviour setup

1. Assemble the housing cage (**Supplementary Figure 1**).

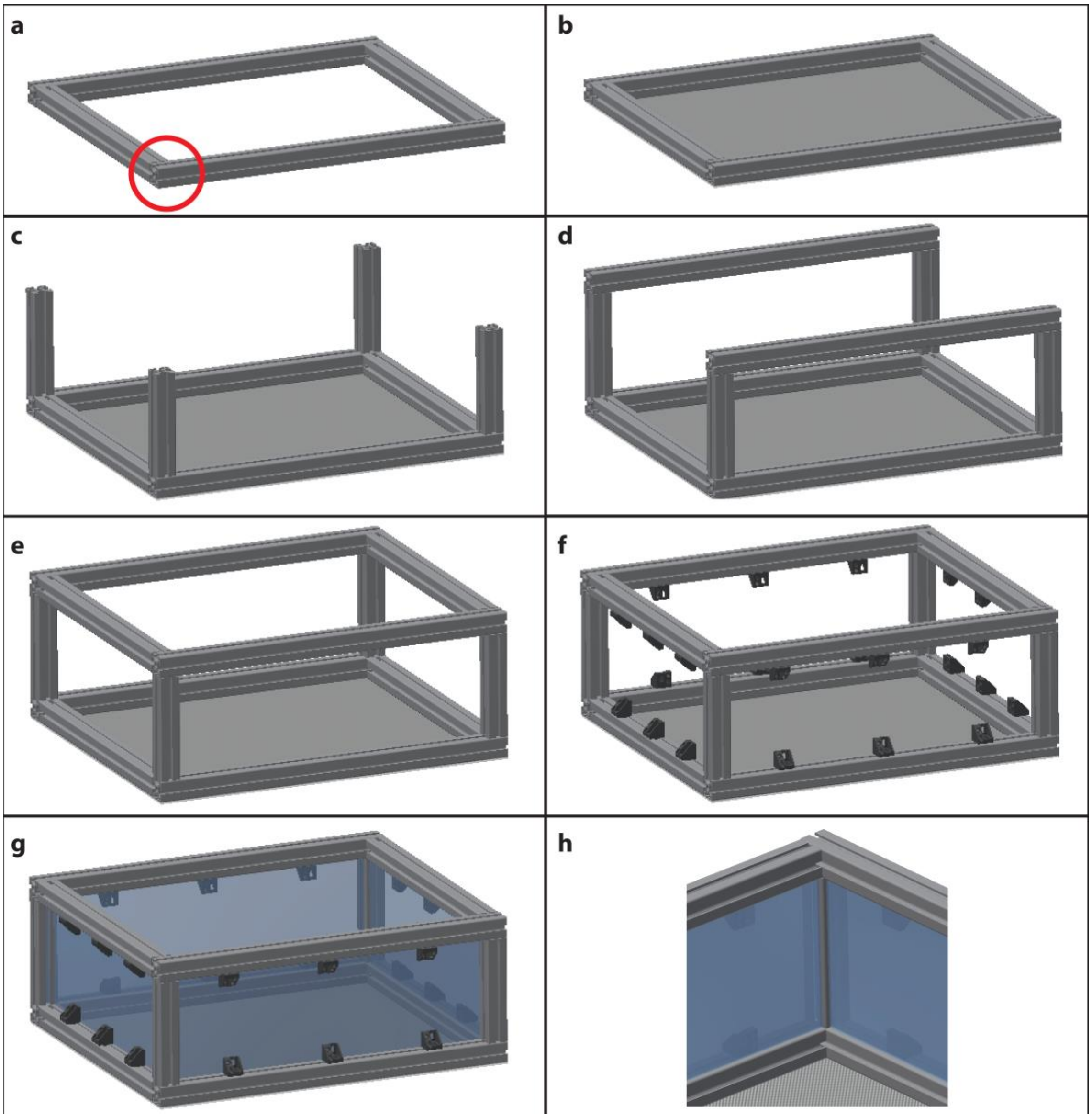

Supplementary Figure 1 Construction of the housing cage. (a) *Assemble the floor using two short (529.8 mm) and two long (700 mm) profiles. The lateral grove of the longer profiles must remain open to allow access for the slot rollers (red circle).* ***(b)*** *Attach the perforated metal sheet to the bottom side of the floor profiles* ***(c)*** *Add the four vertical corner profiles (200 mm).* ***(d)*** *Add the remaining two long (700 mm) profiles.* ***(e)*** *Insert the remaining two short (529.8 mm) profiles.* ***(f)*** *Place the mounting blocks on the profiles.* ***(g)*** *Insert the macrolon plates into the cage and fix them in place using the mounting blocks.* ***(h)*** *The macrolon plates should be placed flush against the metal profiles.*

2. Assemble the bedding stopper (**Supplementary Figure 2**).

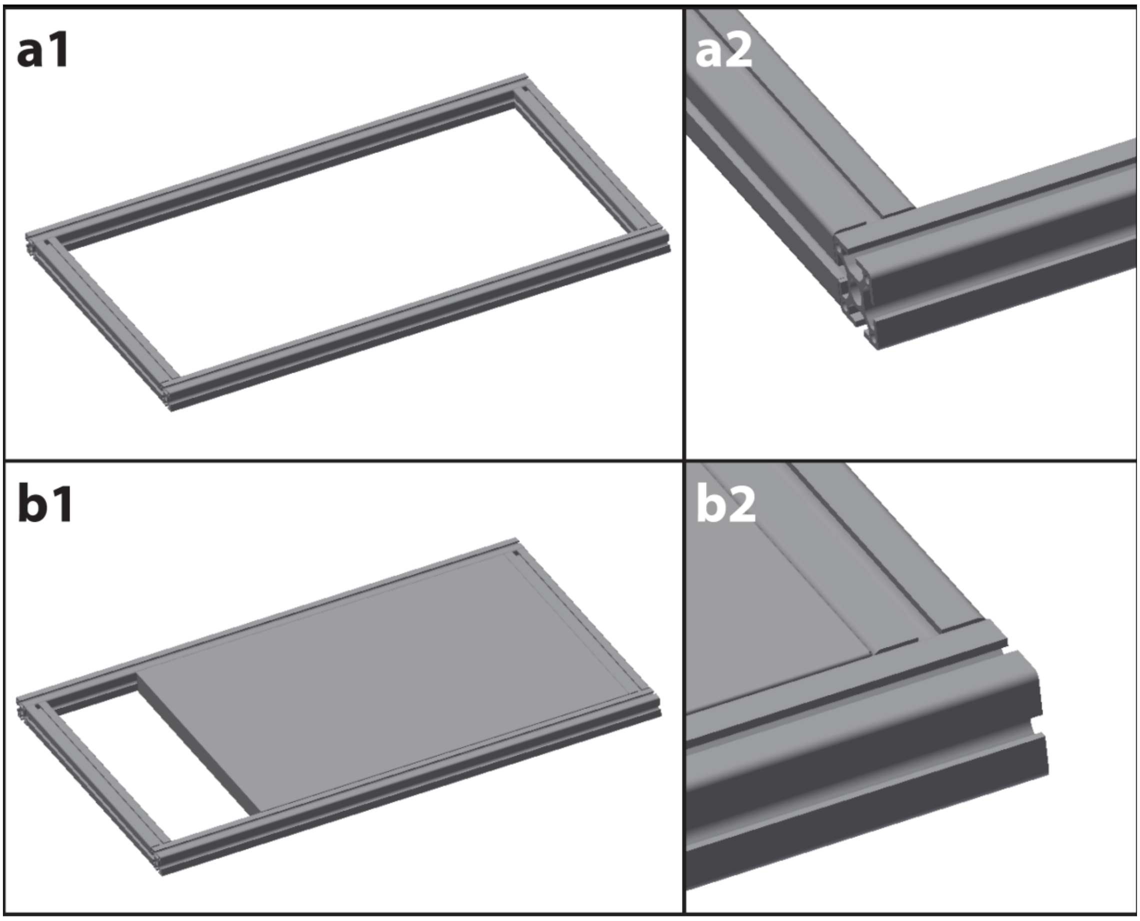

Supplementary Figure 2 Construction of the bedding stopper. (a1) *Assemble the floor using two short (529.8 mm) and two long (950 mm) profiles.* ***(a2)*** *The lateral grove of the longer profiles must remain open to allow access for the slot rollers.* ***(b1)*** *Attach the metal sheet to the insides of the profiles.* ***(b2)*** *The top surface of the metal sheet should form a flat continuous surface with the top sides of the profiles.*

3. Assemble the housing cage lid (**Supplementary Figure 3**).
    a. Some acrylic glass parts need to be glued and some adhesives are toxic when inhaled.

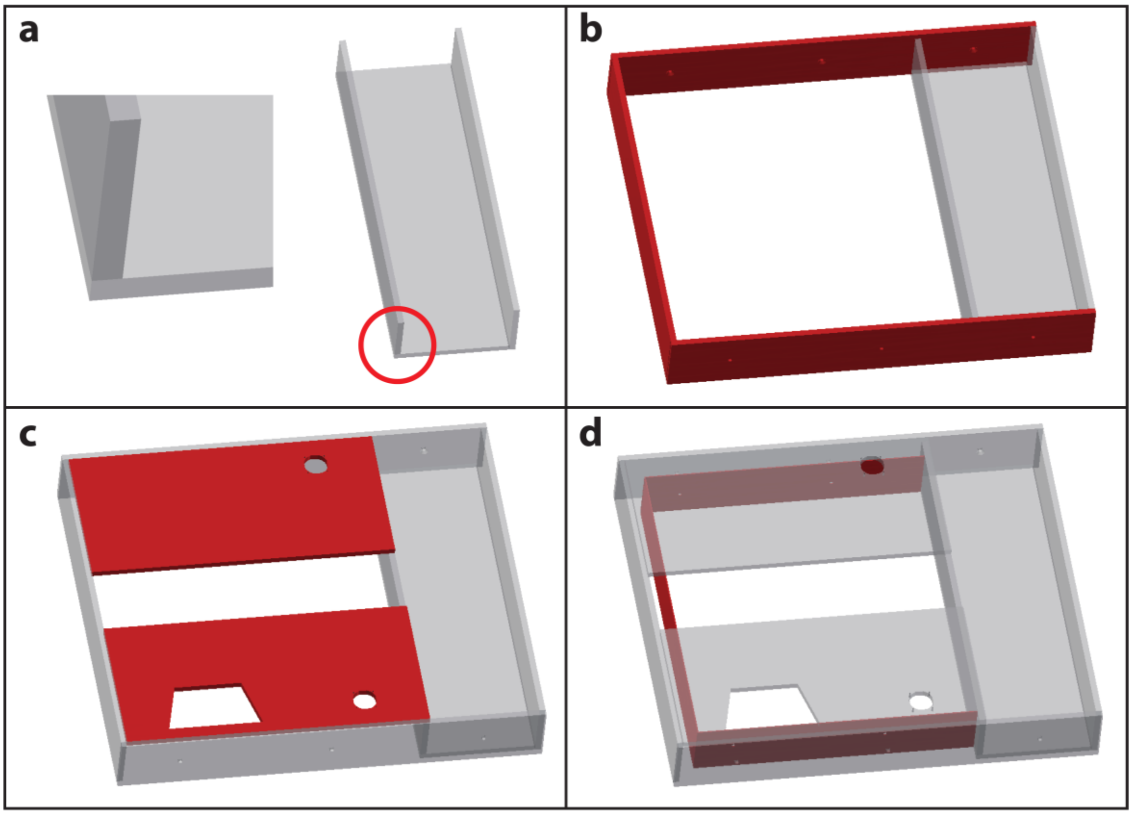

Supplementary Figure 3 Construction of the housing cage lid. (a) *Place the lateral side panels of the scale compartment onto the floor plate of the scale compartment. The side panels should be on top of the floor plate.* ***(b)*** *Attach the two side panels (700 mm length; with drill holes) and the front panel (600 mm length) to the scale compartment.* ***(c)*** *Attach the two top panels to the lid.* ***(d)*** *Attach the inlet places to the inside of the cage lid. The inlet plates should be spaced approximately 40 mm from the inner sides of the cage lid.*

4. Assemble the tunnel leading to the odour port (**Supplementary Figure 4**).
   a. Some acrylic glass parts need to be glued and some adhesives are toxic when inhaled.

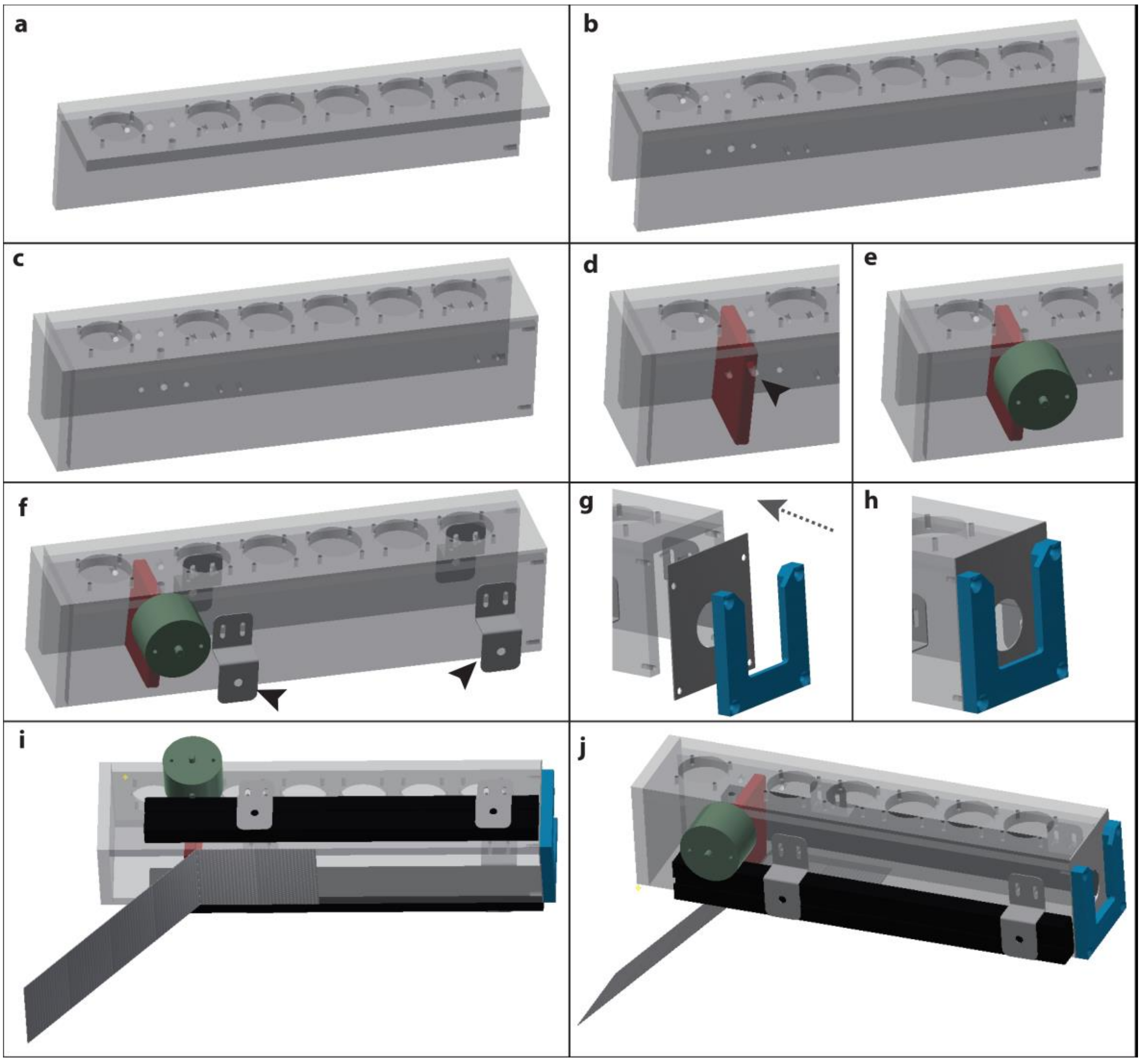

Supplementary Figure 4 Construction of the tunnel leading towards the odour port. (a) *Attach the lid with the fan holes to the first lateral tunnel panel (ventilation holes facing upwards).* ***(b)*** *Add the second lateral tunnel panel.* ***(c)*** *Attach the front tunnel panel. All of these pieces* ***(a-d)*** *should be glued.* ***(d)*** *Insert the door (shown in red) and align the hole in the door with the central hole of the three motor holes (arrow).* ***(e)*** *Insert the motor into the central hole and fix it in place using the two lateral screw threads.* ***(f)*** *Attach the four IR beam holders (highlighted with arrows) to both lateral sides of the tunnel.* ***(g)*** *Place the tunnel back cover (dark grey) and the sliding mechanism for the odour port (blue) at the back of the tunnel.* ***(h)*** *Fix both pieces simultaneously using the four screw-holes located at the back of the tunnel.* ***(i)*** *Slide the two IR curtains into the IR Beam holders and attach the perforated metal sheet to the bottom of the tunnel. The fold in the perforated metal sheet should be located roughly at the relaxed position of the door.* ***(j)*** *The finished tunnel can be placed on the cage lid.*

5. Mount the tunnel onto the housing cage lid (**Supplementary Figure 5**).

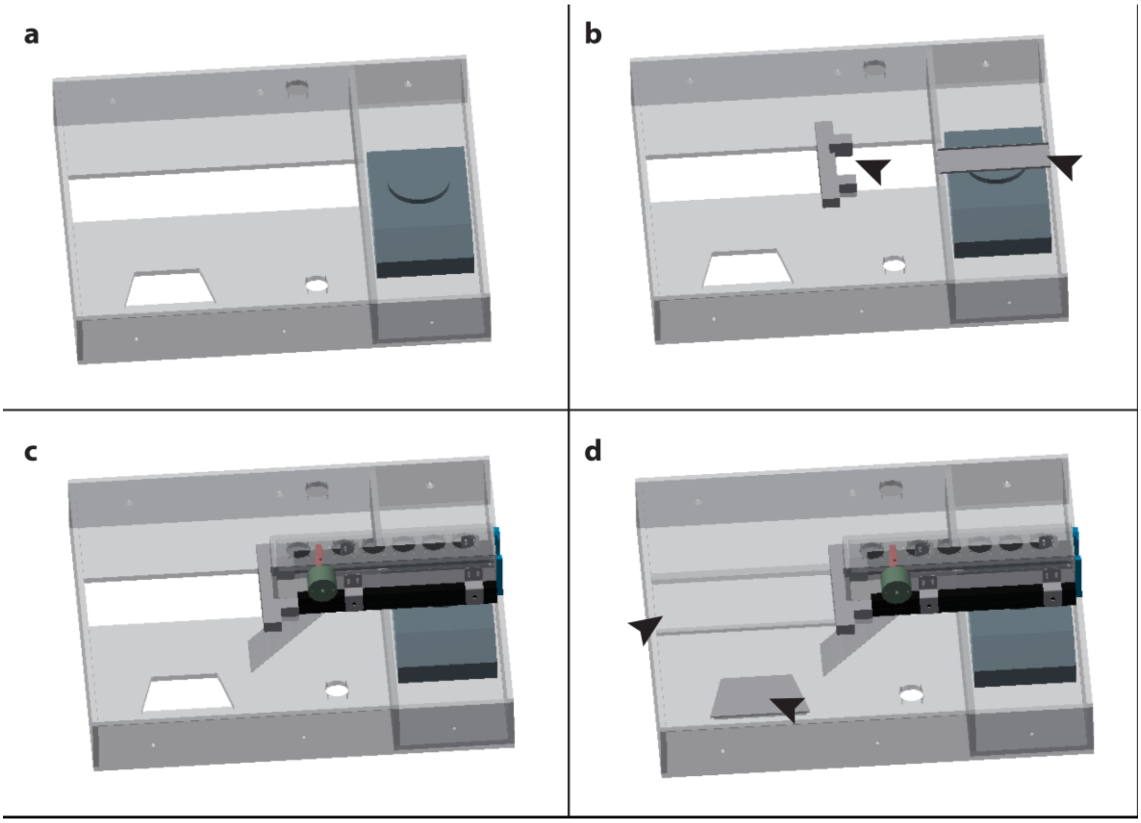

Supplementary Figure 5 Placement of the tunnel onto the housing cage lid. (a) *Place the scale into the scale compartment of the behaviour cage lid.* ***(b)*** *Place the tunnel holder between the two lid top plates and the tunnel floor plate onto the scale (arrows).* ***(c)*** *Place the assembled tunnel into the holder. The floor plate should be flush against the insides of the tunnel.* ***(d)*** *Add the lid for the food hopper (arrow) and the maintenance cover (arrow) to close the lid.*

6. Assemble the setup frame (**Supplementary Figure 6** - **Supplementary Figure 8**) and directly mount the pre-assembled housing cage lid, housing cage and bedding stopper in the frame.

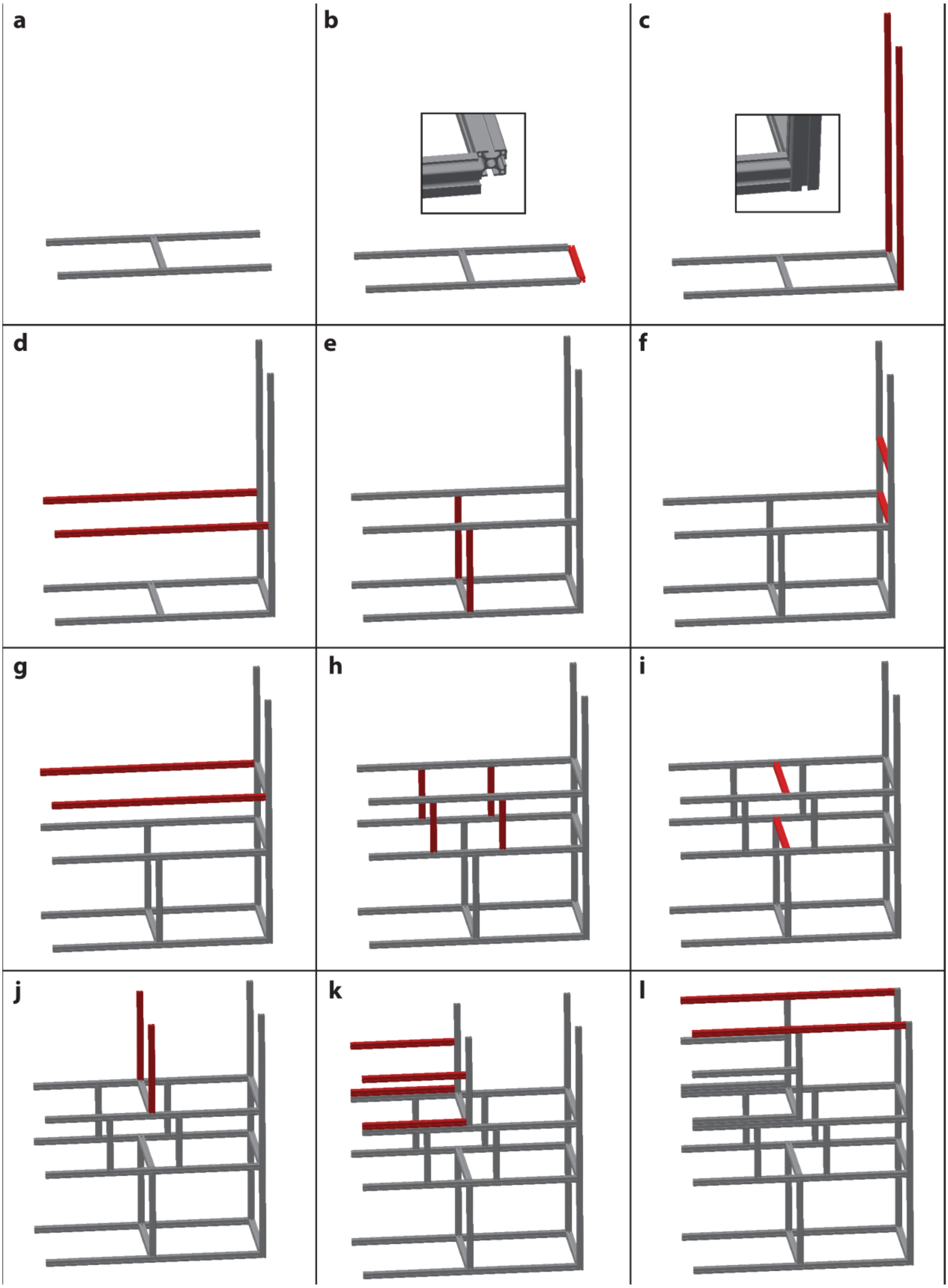

Supplementary Figure 6 Part 1 of the assembly of the setup frame. (a) *Assemble two 1360 mm and one 620 mm aluminium profile in the form of the letter "H".* ***(b)*** *Place another 620 mm profile on one end of the H leaving a gap at the corner.* (c) *Add two vertical beams (1627 mm) into the free corner.* ***(d)*** *Add two 1360 mm profiles horizontally spaced 550 mm from the bottom profiles.* ***(e)*** *Add two vertical 550 mm beams.* ***(f)*** *Add two 620 mm beams with one spaced 550 mm from the bottom and the second 880 mm from the bottom.* ***(g)*** *Add two 1360 mm profiles horizontally spaced 880 mm from the bottom.* ***(h)*** *Add four vertical beams (330 mm) between the horizontal beams. The rightmost two should be spaced roughly 490 mm from the vertical beams to accommodate the NIM-Bin power supply.* ***(i)*** *Add two 620 mm beams.* ***(j)*** *Add two vertical beams (587 mm)*

spaced roughly 660 mm from the right-hand vertical beam. ***(k)*** *Add four horizontal beams (660 mm). The bottom ones should sit flush on top of the vertical beams and will hold the rollers for the housing cage. The top ones should be placed roughly 278 mm higher and will serve as the anchor point for the housing cage lid.* ***(l)*** *Add two 1360 mm horizontal beams at the top of the setup.*

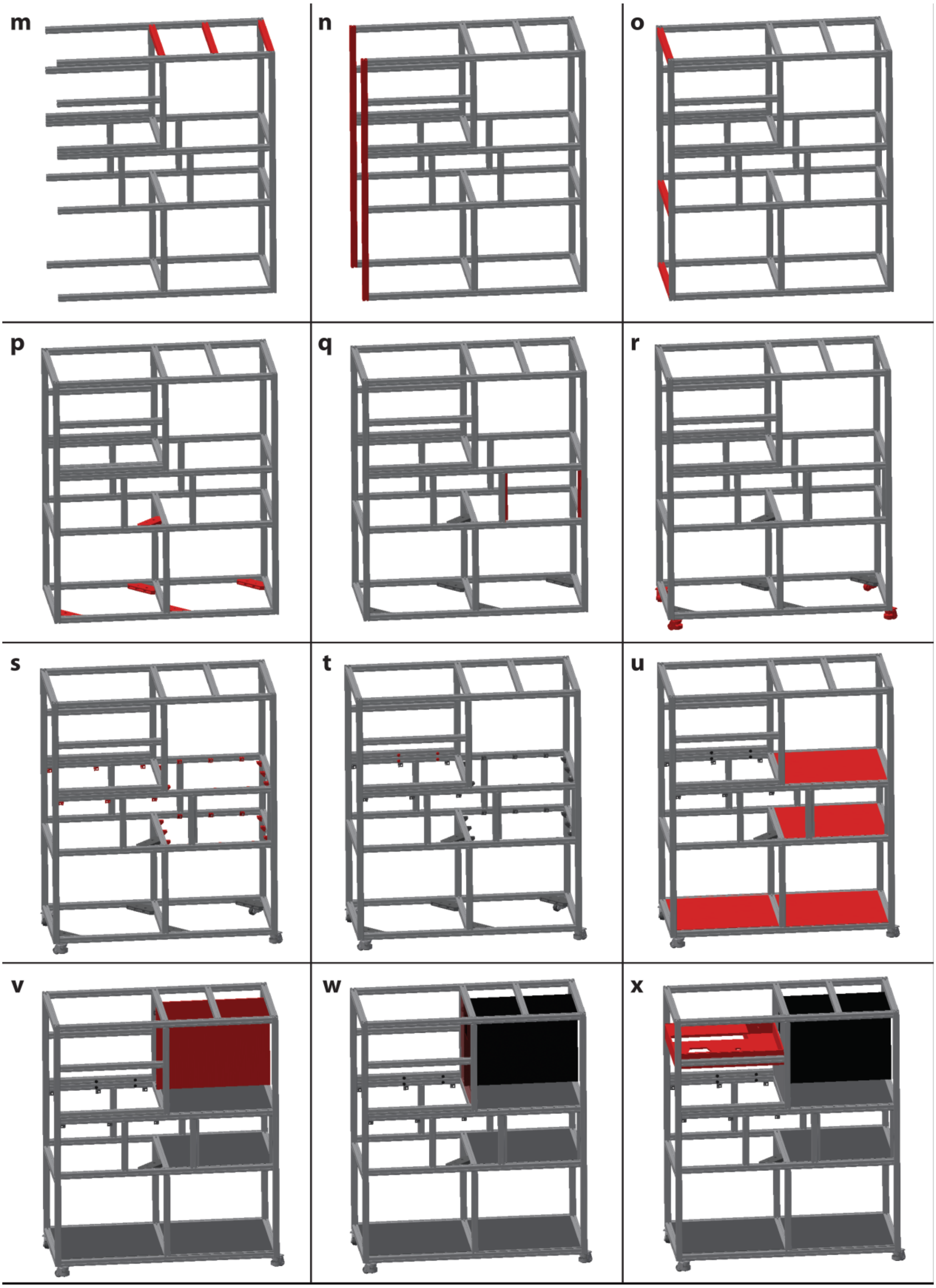

Supplementary Figure 7 Part 2 of the assembly of the setup frame. (m) *Add three 620 mm profiles at the top of the setup. The leftmost one should be the same position as the vertical beams installed in* ***(j)****.* ***(n)*** *Add two vertical 1627 mm beams at the left side of the setup.* ***(o)*** *Add three 620 mm beams at the left side of the setup.* ***(p)*** *Add the corner pieces to stabilize the bottom and middle compartments of the frame.* ***(q)*** *Add the attachments for 19'' profiles in the right-hand middle compartment. These will be used to secure the NIM-Bin power supply in place.* ***(r)*** *Add the wheels to the bottom of the setup.* ***(s)*** *Add mounting blocks to support the PVC plates in the middle and top compartment.* ***(t)*** *Add the slot rollers for the housing*

cage and the bedding stopper. Make sure to use the appropriate M8 T-nuts to fasten the slot rollers into the profile. ***(u)*** *Add the PVC plates for the three compartments and secure them in place using the mounting blocks.* ***(v)*** *Add the pneumatics backplane to the right-hand top compartment.* ***(w)*** *Add the backplane for the mouse housing cage.* ***(x)*** *Add the lid of the housing cage.*

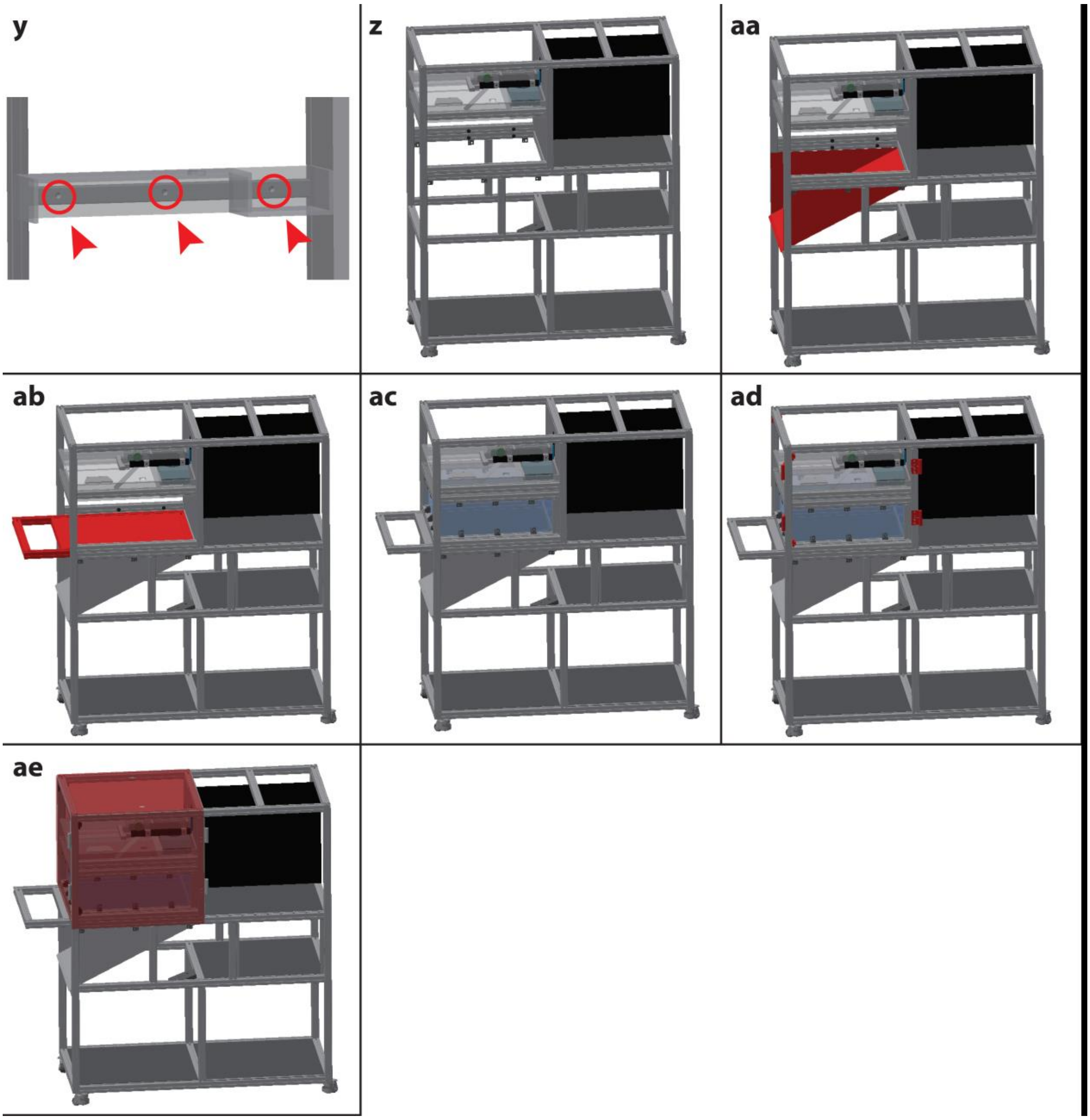

Supplementary Figure 8 Part 3 of the assembly of the setup frame. (y) *Ensure that the T-nuts are placed at the correct location before inserting the housing cage lid into the setup frame.* ***(z)*** *Add the tunnel to the housing cage lid.* ***(aa)*** *Add the metal bedding shute.* ***(ab)*** *Insert the bedding stopper.* ***(ac)*** *Insert the housing cage.* ***(ad)*** *Add the aluminium hinges and magnetic locks for the setup covers.* ***(ae)*** *Add the cover plates to enclose the setup.*

7. Attach the food hopper and the ladder giving mice access to the ramp (**Supplementary Figure 9**).

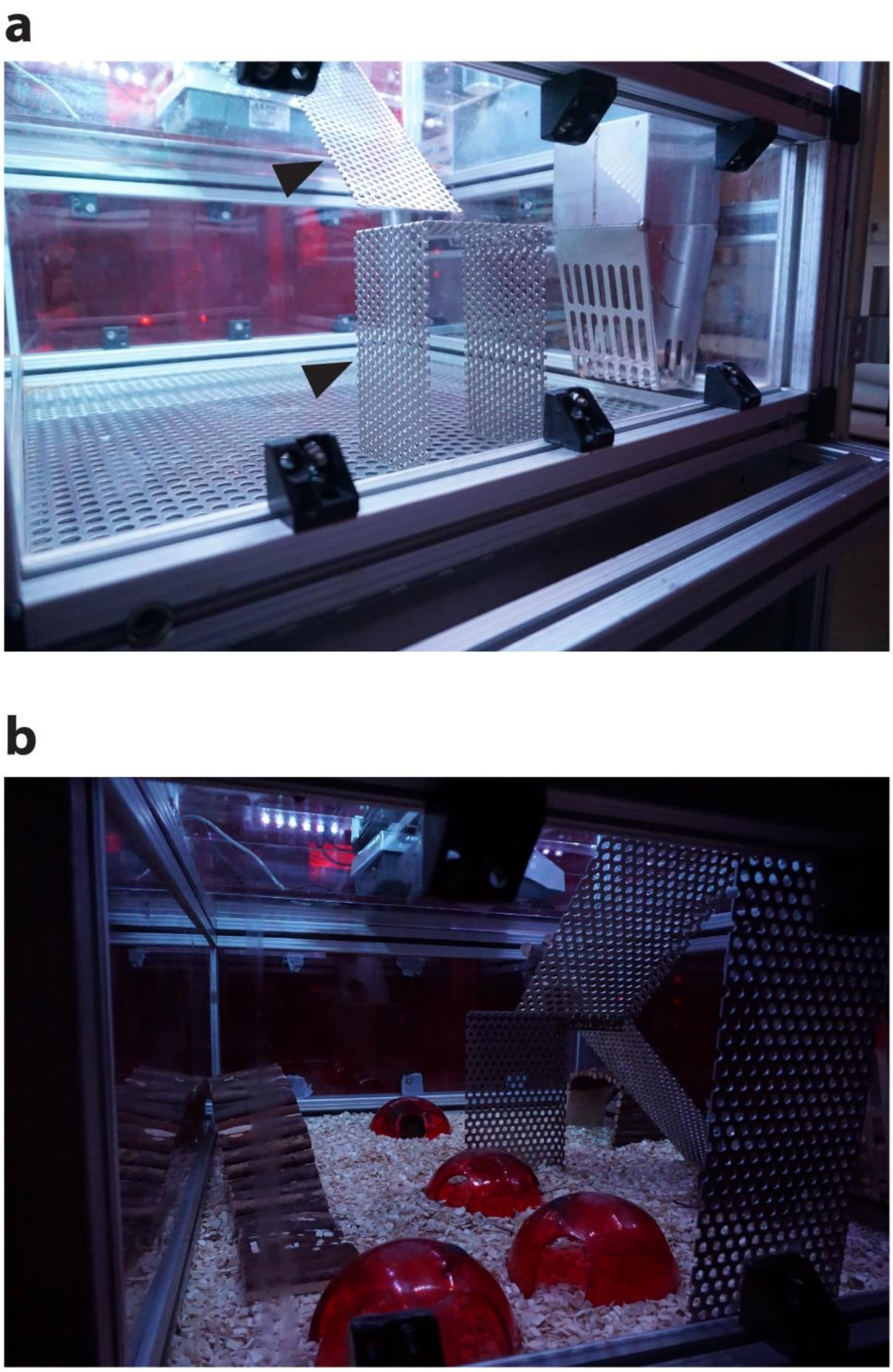

Supplementary Figure 9 Overview of the inside of the housing cage. (a) *Inside of the housing cage without bedding or enrichment showing placement of the ramp (arrow) underneath the ladder (arrow).* ***(b)*** *Fully supplied housing cage.*

8. Assemble the odour port and slide it in the holder at the back end of the tunnel (**Supplementary Figure 10**).

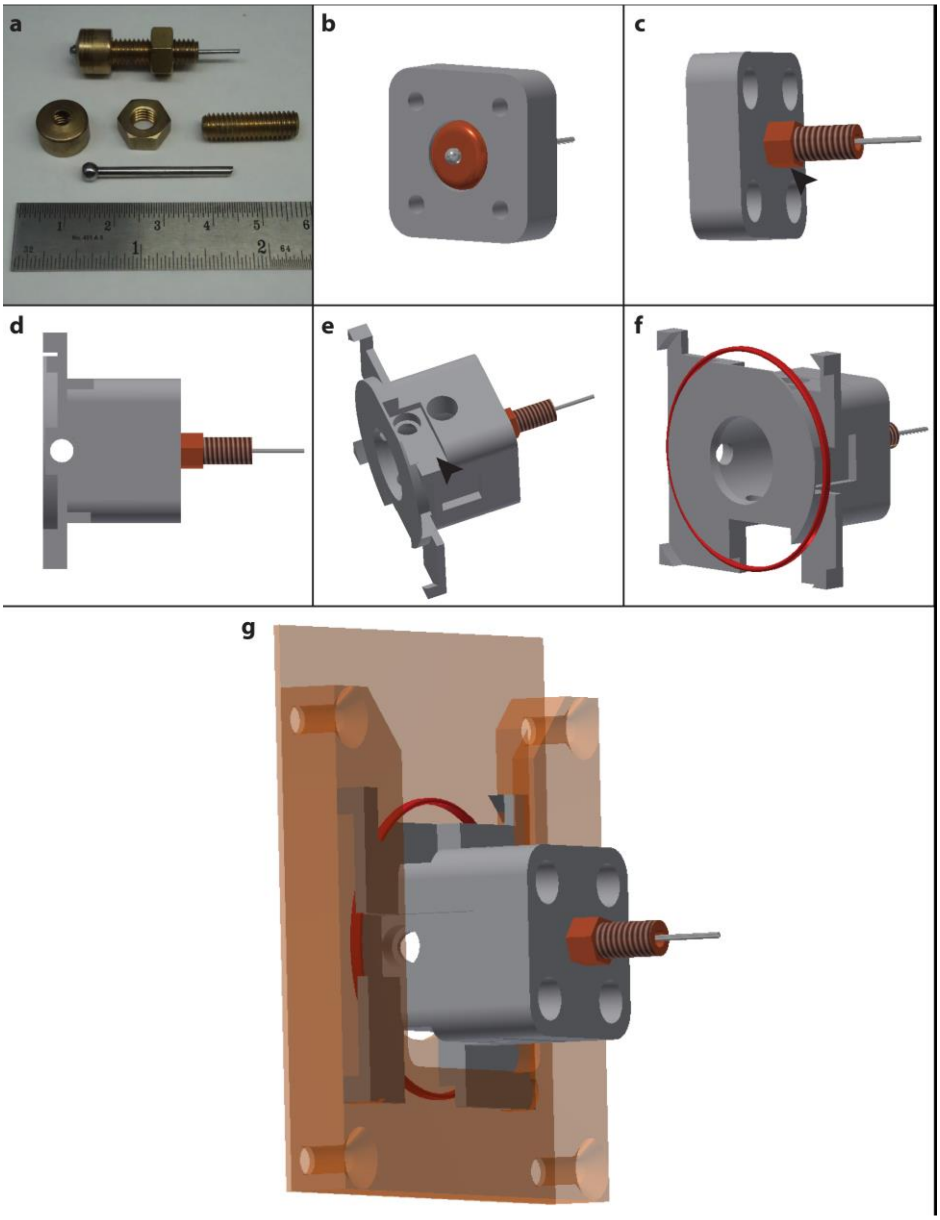

Supplementary Figure 10 Assembly of the odour port. (a) *Details of the custom made lick spout. The lick spout consists of a shortened 14G-feeding tube enclosed in a threaded copper pipe and copper cap.* ***(b)*** *Insert the lick spout through the central hole of the odour port back plate.* ***(c)*** *Fix the lick spout in place by screwing the copper nut (arrow) onto the threaded copper pipe.* ***(d)*** *Fix the back plate to the main odour port body using the four screw holes located on the back of the odour port body. Both pieces should be tightly fixed leaving no gaps in between.* ***(e)*** *Insert the IR Beams and IR Detectors into the cavities (arrow) on the outside of the odour port.* ***(f)*** *Place the RFID Reader Antenna (red) into the grove in the front side of the olfactometer.* ***(g)*** *Finished odour port assembly placed into the sliding holder fixed to the back end of the tunnel (both translucent orange).*

9. Attach an LED-strip (Adafruit, #285) to the setup frame above the housing cage lid to provide lighting for the housing cage and connect it to a timer clock (Conrad, #616020-62).
10. Assemble the water delivery system and mount it on the setup frame above the odour port (**Supplementary Figure 11**). The dosing nozzle of the water delivery system is inserted into the lick spout.
    a. The lick spout should not be sealed around the dosing nozzle to prevent mice from forcefully sucking water out of the delivery system if no reward is given.

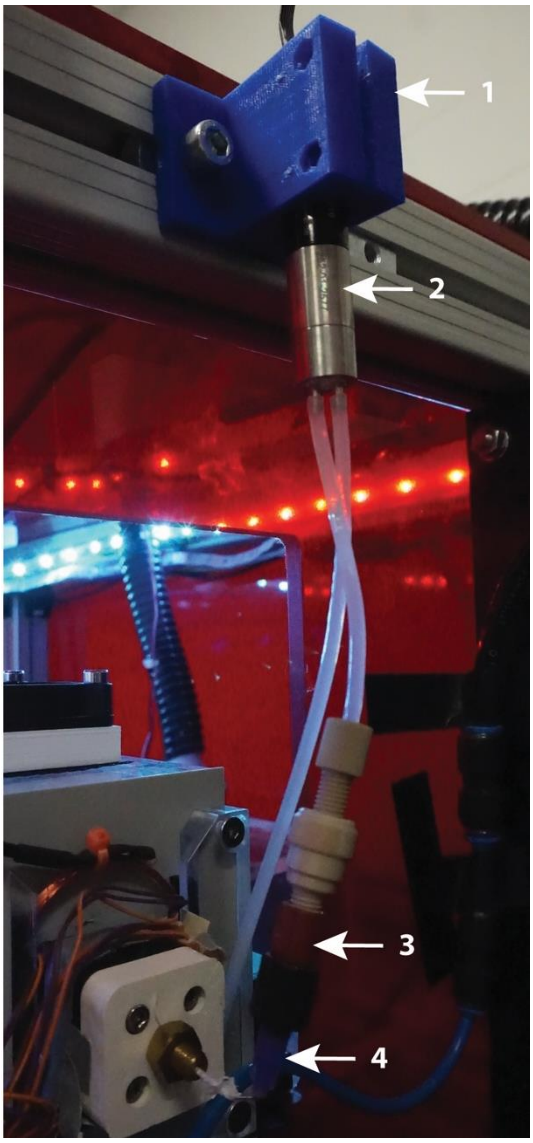

Supplementary Figure 11 Detailed overview of the water delivery system at the odour port. ***(1)*** *Custom 3D printed holder.* ***(2)*** *Micro Annular Gear Pump.* ***(3)*** *Pipe Fitting, Filter and Fluid Adapter.* ***(4)*** *Dosing Nozzle.*

11. Assemble the olfactometer (**Supplementary Figure 12**) and mount it to the pneumatics back plane of the setup frame.

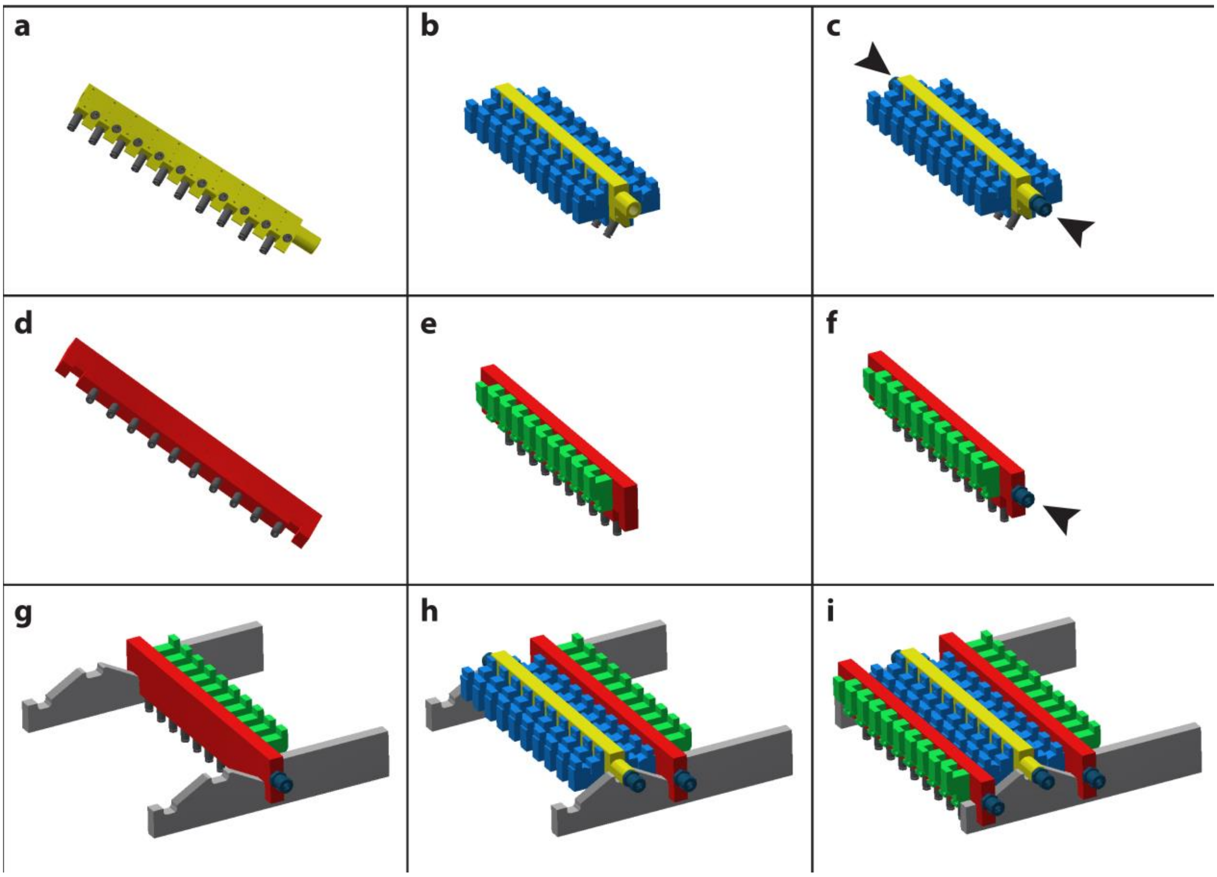

Supplementary Figure 12 Assembly of the olfactometer. ***(a)*** *Screw the odour tubing fittings (SMC, #KQ2S23-M5N) into the threaded holes at the tapered bottom edges of the central manifold (yellow).* ***(b)*** *Fix the 2/2-Odour Valves (Asco #SCS067A028) to the central manifold with the connector pins facing upwards.* ***(c)*** *Fix the tube connectors (Festo, #CK-1/8-PK-4) to the air inlet and outlet at the front and back of the central manifold (arrows).* ***(d)*** *Screw the odour tubing fittings (SMC, #KQ2S23-M5N) into the threaded holes along the bottom edges of the outer olfactometer channels (red).* ***(e)*** *Fix the 3/2-Micro Odour Valves (Asco # 18801088) to the outer olfactometer channels with the connector pins facing upwards.* ***(f)*** *Fix the tube connectors (Festo, #CK-1/8-PK-4) to the air inlet at the front of the outer olfactometer channels (arrow).* ***(g)*** *Place the right-hand side outer olfactometer channel into the olfactometer holders with the tube connector facing to the front and the valve connector pins facing upwards.* ***(h)*** *Place the central manifold into the olfactometer holder with the elongated side facing the front and the valve connector pins facing upwards.* ***(i)*** *Place the left-hand side outer olfactometer channel into the olfactometer holders with the tube connector facing to the front and the valve connector pins facing upwards.*

12. Fix the air filters to the back of the pneumatics back plane of the setup frame (**Supplementary Figure 13**).

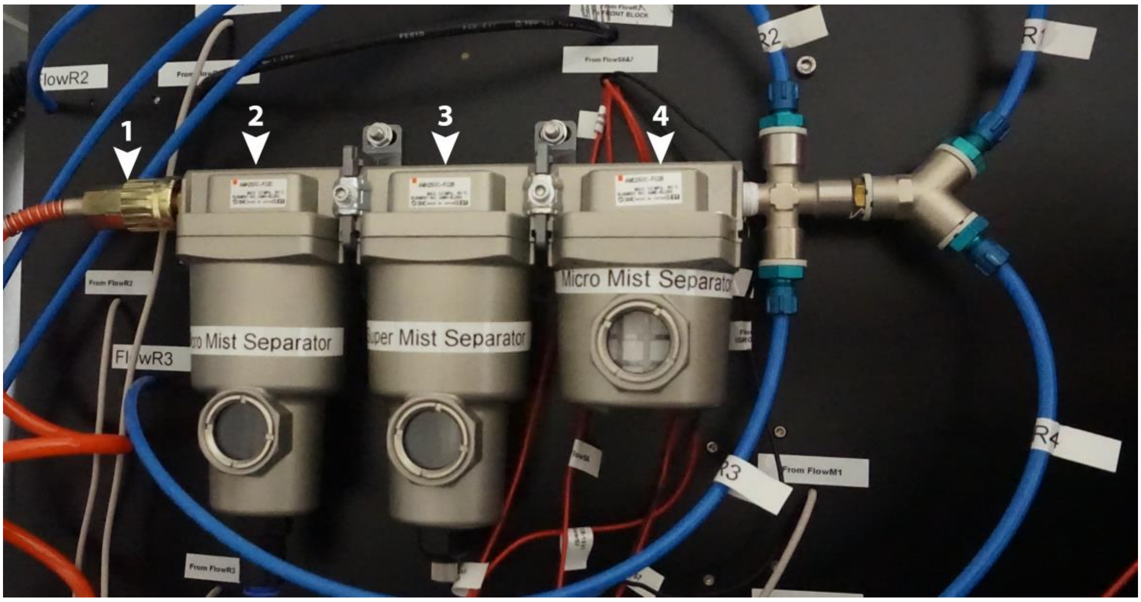

Supplementary Figure 13 Detailed placement of the air filters on the pneumatics back plane. (1) *Pressurized air input.* ***(2)*** *Micro Mist Separator (SMC, # AMH250C-F02D AMH-EL250).* ***(3)*** *Micro Mist Separator (SMC, # AMH250C-F02 AMF-EL250).* ***(4)*** *Supermicro Mist Separator (SMC, # AMH250C-F02 AME-EL250).*

13. Mount the flow sensors and pressure reducers to the front of the pneumatics back plane of the setup frame (**Supplementary Figure 14** - **Supplementary Figure 15**).

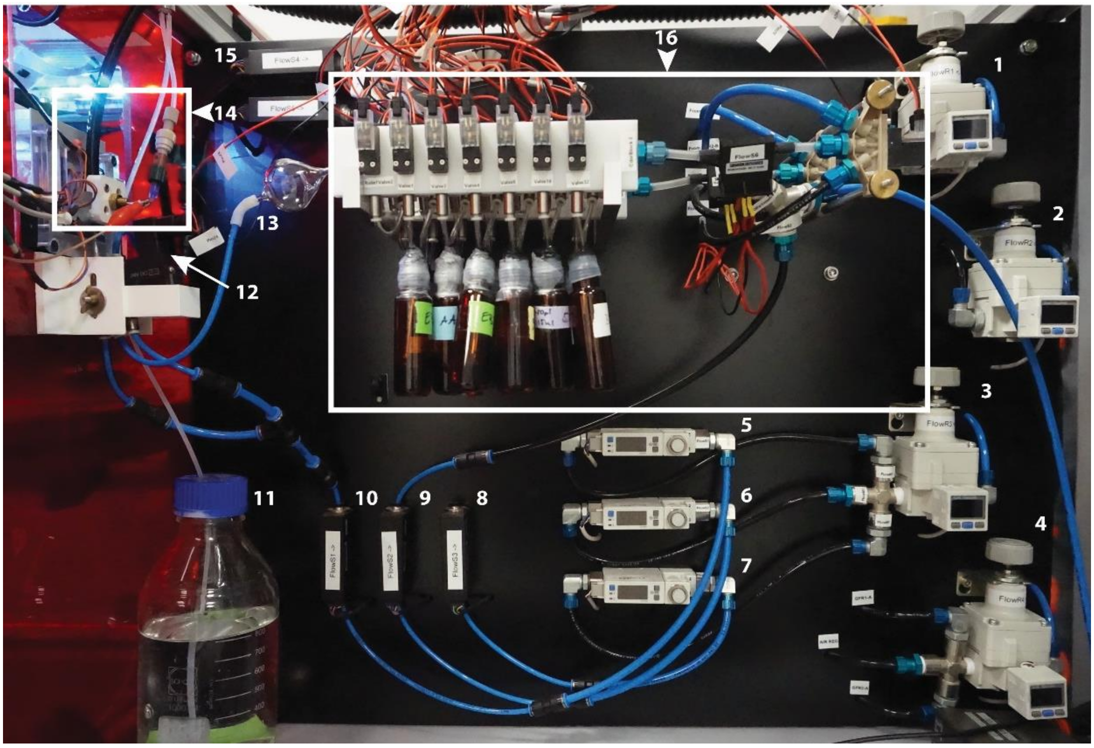

Supplementary Figure 14 Detailed overview of the front of the pneumatics back plane. (1-4) *Pressure reducers (SMC, #IR2000-F02).* ***(5-7)*** *Flowmeters (SMC, #PFM710S-F01-E).* ***(8-10)*** *Flow sensors (First Sensor, #WTAL005DUP).* ***(11)*** *Water reservoir.* ***(12)*** *Final Valve in 3D printed holder.* ***(13)*** *Glass air mixing bowl.* ***(14)*** *Odour Port with attached water delivery system.* ***(15)*** *Exhaust flow sensor (First Sensor, #WTAL010DUP).* ***(16)*** *Olfactometer and olfactometer input regulator.*

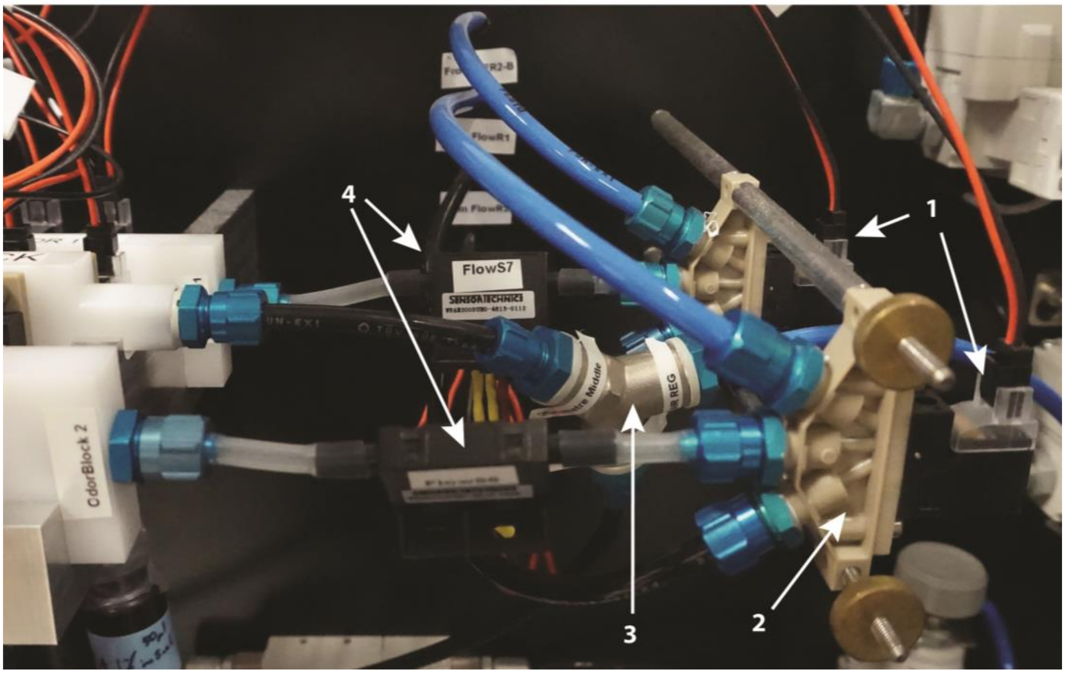

Supplementary Figure 15 Detailed overview of the input connections into the olfactometer. (1) *Prepulse valves (Asco #SCS067A108).* ***(2)*** *Valve Mounting Block (Asco, #36100040).* ***(3)*** *Y-Connectors (Festo, #NPFC-Y-3G14-F).* ***(4)*** *Flow sensors (First Sensor, #WBAM200DuHo).*

14. Mount the NIM-Bin Power supply unit into the right-hand middle compartment of the setup frame and fix it to the 19’’-profiles.
15. Insert the Electronics Driver Modules into the NIM-Bin Power Supply (**Supplementary Figure 16**).

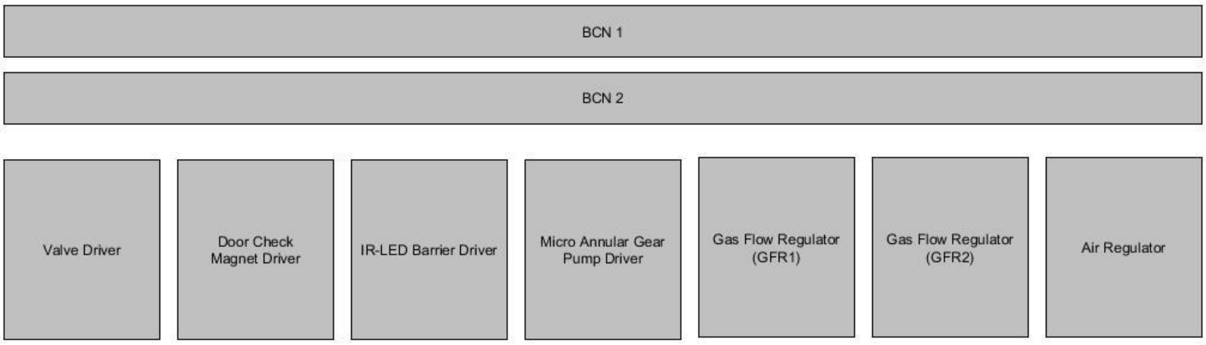

Supplementary Figure 16 Recommended placement of the electronics modules in the NIM-Bin power supply. *BNC 1 and BNC 2 are connected to the first computer (running the Behaviour Software) and the second computer (running the Sensor Acquisition Software) respectively. The BNC Connector Blocks should be placed above the NIM-Bin Power supply unit and fixed to the 19’’-profiles. All Electronics Driver Modules should be securely fixed to the NIM-Bin Power supply using the screws at their respective front panels.*

16. Mount the BNC Connector Blocks above the NIM-Bin Power Supply Unit and fix them to the 19’’-profiles (**Supplementary Figure 16**).

17. Connect the air-flow tubing according to the connection diagrams (**Supplementary Figure 14** - **Supplementary Figure 15** and Part 2 of the Supplementary Information).
18. Set up the electrical connections as detailed in Part 2 of the Supplementary Information.
    b. Improper wiring can be hazardous. Carefully check the manufacturer's specification for each electronic device before wiring it to the setup.
19. Install the PCI I/O devices according to the manufacturer's instructions and place the computers into the right-hand bottom compartment of the setup frame
20. Install the behaviour software as detailed in Part 3 of the Supplementary Information.
    c. Make sure all peripheral devices as well as input and output ports are correctly connected and properly referenced in the software.
21. Connect a constant source of pressurized air or carrier gas of choice to the air-filters on the back of the pneumatics back pane.
    d. We recommend use of a central air supply as local gas bottles would require constant replacement to maintain constant air flow
    e. Improper connection of pneumatic tubing can cause rupturing of tubes. Carefully check the connections before supplying pressurized air to the setup.
22. Adjust the baseline air-flow speed and pressure by adjusting the air flow meters and pressure regulators respectively.

Preparation of odour reservoirs

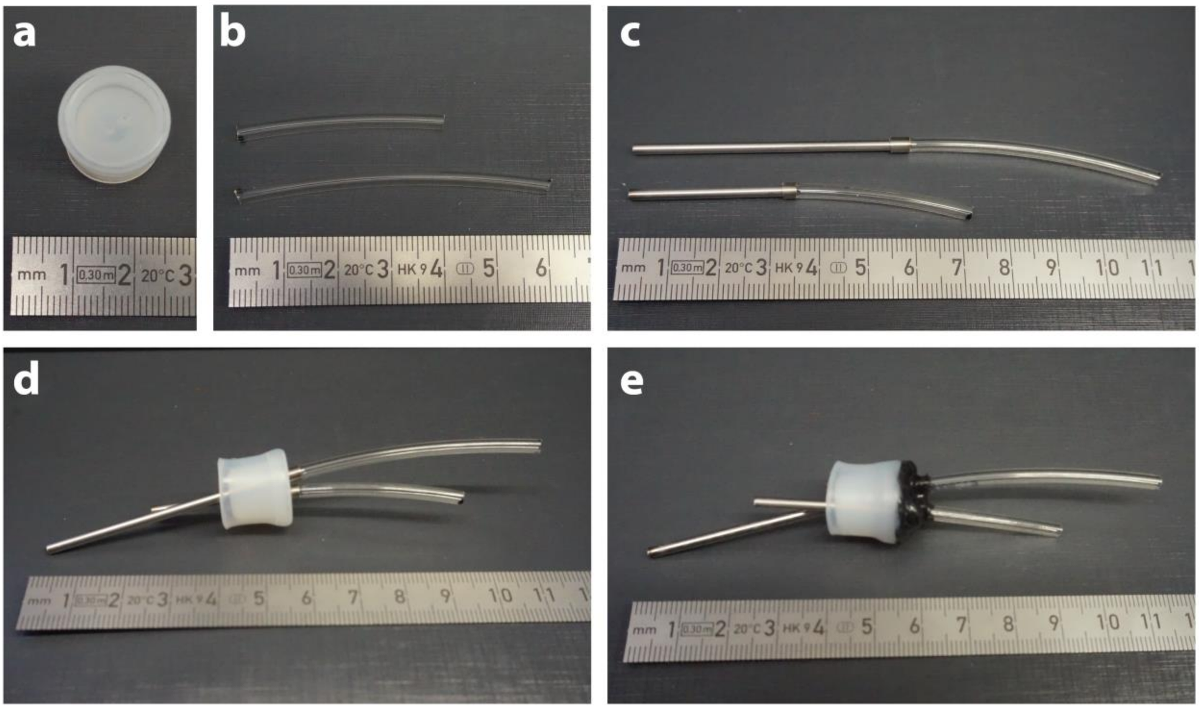

Supplementary Figure 17 Preparation of the odour reservoir caps. (a) *Silicone bottle cap with two centrally placed drill holes.* ***(b)*** *Pre-cut odour reservoir tubing.* ***(c)*** *The pre-cut stainless steel pipes are inserted into the odour tubing approximately 4-5mm deep.* ***(d)*** *One long and one short metal pipe with attached tubing are inserted into the holes of the silicone bottle cap.* ***(e)*** *Finished bottle cap. The outer top part of the silicone cap is thoroughly sealed with silicone (black) fixing the metal pipes and tubing in place.*

23. Drill two holes (drill diameter approx. 3 mm) into each silicone bottle cap (**Supplementary Figure 17**a).
24. Cut the odour tubing (#TIUB01C-20) into pieces of approx. 6 cm and 4 cm length. Each bottle cap requires one short and one longer piece of tubing (**Supplementary Figure 17**b).
25. Insert the short metal pipe into the short tubing piece and the long metal pipe into the longer tubing piece (**Supplementary Figure 17**c,b).
26. Insert one short and one long metal pipe with the attached tubing into the silicone caps (**Supplementary Figure 17**d).
27. Seal the silicone caps with silicone adhesive (**Supplementary Figure 17**e) and let it fully cure according to the manufacturer's specifications (i.e. over-night).
    a. Alternatively, the metal pipes can be omitted and longer odour tubing (12 cm and 7.5 cm respectively) can be used instead. We strongly advise against this, as any pressure applied to the bottle cap can exert pressure to the tubing which could cause fatigue of the tubing and impair the air flow.

Olfactometer preparation and validation

General considerations for odour application and validation of the olfactometer

It has to be noted that the odour used for validation of the olfactometer (2-Butanone) was not used during any behavioural experiments but used only for validation of the olfactometer due to its excellent compatibility with our ionisation detector and its high volatility. In our hands, use of this odour ensured reliable, stronger and less noisy signals compared to the odours used during the behavioural tasks. This is especially important, since the ionisation detector is very sensitive and very susceptible to slight changes in odour concentration We strongly recommend users familiarize themselves with optimal placement of the ionisation detector and the effects of incorrect location of the sensor (see [1] for a rigorous analysis) prior to starting the measurements.

In contrast, amyl acetate resulted in far smaller signals, however the shape of the odour pulse remained fairly stable (and square like) for the initial 1000 ms of odour application (**Supplementary Figure 18**a). Amyl acetate also resulted in slightly higher times for the odour onset (**Supplementary Figure 18**b) as well as more variable times for the peak of the odour concentration (**Supplementary Figure 18**c).

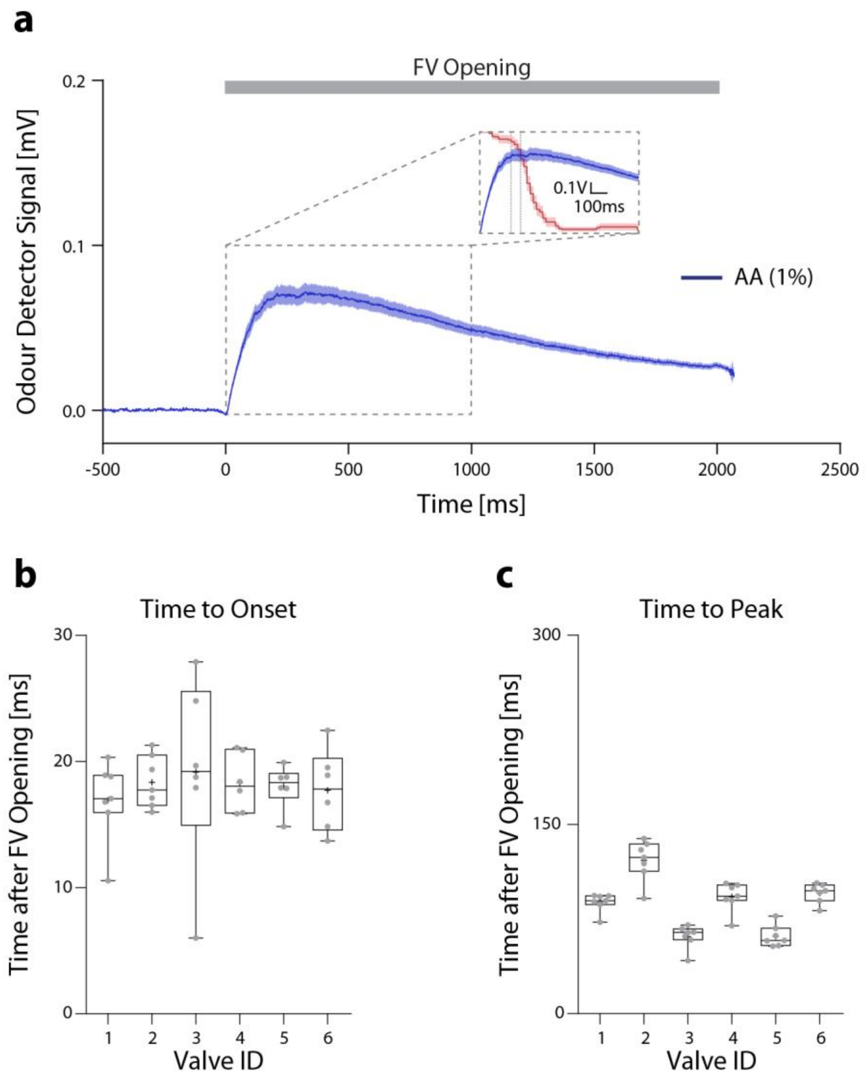

Supplementary Figure 18 High resolution temporal analysis of the presentation of amyl acetate. (a) *Representative signal obtained using amyl acetate (AA; 1% in mineral oil; n=6). Note the scale of the measured signal. Inset shows the initial 1000 ms of odour presentation (blue) superimposed on a representative sampling trace of one animal for unrewarded trials (red) as well as the discrimination times for this animal using a p-value if 0.05 and 0.001 respectively (grey vertical dashed lines).*

The scale of the sampling rate has been adjusted to a rate of 0.1 being equal to a PID signal of 0.1V. Time until ***(b)*** *odour onset and* ***(c)*** *peak odour concentration was reached relative to opening of the final valve (n=6).*

Apart from the odour identity and the concentration in the odour reservoir, a constant odour concentration can also be achieved by using larger odour reservoirs or by adjusting the speed of the input air. Generally, odour pulses used for behavioural analysis should ideally have a “square” shape for the entire duration of the odour presentation. Since (i) our usage of the automated behaviour setup focusses on simultaneous comparison of two experimental cohorts (i.e. transgenes and their littermate controls) and not on the determination of fundamental olfactory properties in wild-type mice and (ii) as mice typically respond to any given odour well before 1000 ms (see i.e. [2–5]), the decline in the odour concentration after this time would not have an effect on our test paradigms. However, users wishing to investigate i.e. differences in odour concentrations or odour pulses differing in their temporal sequences should carefully calibrate their odour pulses towards a fully square shape.

28. Supply each brown glass bottle with 5 ml mineral oil containing 2-Butanone (1% v/v), close the bottle with a pre-made silicone cap (**Supplementary Figure 17**) and connect them to the olfactometer by inserting the shorter odour tubing to a tubing fitting located on the outer olfactometer channel and the longer odour tubing to a tubing fitting located on the corresponding position on the central manifold.
    b. The reference odour should be selected based on the odour detector used to validate the olfactometer (for the Aurora Scientific miniPID200B we used 2-Butanone, for other detectors check with manufacturer’s specifications).
29. Leave the olfactometer unperturbed for at least 30 min to ensure full equilibration of the gas phase of the odour bottles.
30. Adjust the air-pressure in the two lines connected to the main gas-flow regulators to 0.025 MPa and the pressure of the two lines supplying the olfactometer to 0.1 MPa by adjusting the connected pressure reducers.
31. Set the baseline air-flow on all air-flow meters to 2 l/min.
    f. Both air-flow and air pressure should be absolutely stable even over months of testing. In case the values show any sign of instability check the tubing for leaks or switch to a more stable source of pressurized air.
32. Position the ionization detector in the odour port directly at the odour inlet, perpendicular to the odour inlet tube.
    a. The detector must be positioned as close to the air outlet as possible as any variation can severely influence the measurements[1].
33. Set the ionization detector to 1x Gain and high Pump levels to maximize the detection range of the sensor.
34. Turn the ionization detector on and wait for the sensor to warm up (as per the manufacturer’s instructions).

   b. Adjust the Offset only after the pre-warming period according the manufacturer's specifications.
35. Run the desired stimulus protocol and verify odour onset and odour peak relative to the opening of the final valve (see Figure 3A-C).
36. The odour onset should occur as fast as possible (i.e. <20 ms) for all valves and the peak odour concentration should be reached in <100-150 ms for all valves. To verify odour dilutions, supply one channel of the olfactometer with mineral oil only and repeat the measurements for at least three dilutions (see Figure 3D-F).

Animal and setup preparations

37. Implant the RFID chips as described in the main paper (also see Bolaños and Murphy[6]). Animals should be allowed to recover for at least 5 days before the start of the training but can be group housed directly following surgery in a dedicated group-housing cage. Correct placement of the RFID chip should be monitored after surgery.
    a. If the phenotype of the mice needs to be induced by i.e. stereotaxic delivery of viruses or by other means that require anaesthesia of the animals, the RFID surgery could be combined with the initial procedure to minimize the need for multiple anaesthesia.
38. Supply the setup housing cage with wood or plastic-based houses and enrichment (i.e. chew toys, running wheels, tunnels, nesting material, etc.).
    a. Avoid tissues or tissue-based nesting materials as mice can move this to the odour port and clog the port and the IR beam.
39. Set the light/dark cycle as desired using the timer clock.
40. Supply the olfactometer with the odours to be tested as well as blank samples containing the solvent (i.e. mineral oil) only. Present each odour from at least two individual valves and preferably include an initial training-odour pair (i.e. Cineol vs Eugenol) used only to train the animals on the odour discrimination aspect of the go/no-go task.
41. Set the behaviour training software with the RFID codes and the desired training protocols (see Part 3 of the Supplementary Information).
42. Restrict access to the odour port by closing the door or removing the ramp leading to the tunnel and transfer the mice to the housing cage of the behaviour setup.
    a. Check functionality and placement of the RFID chip using the hand-held RFID detector before placing them in the setup.
43. Wait at least one hour before opening access to the odour port to allow the animals to acclimatize to the new cage while closely monitoring them for aggressive behaviour.

a. If the mice were not group housed prior to moving them to the setup consider restricting access to the odour port for longer (i.e. 5-12 hours) while providing water in the housing cage.

Automated behaviour training

44. Allow access to the odour port to start the pre-training phase. As mice naturally like to explore their environment, they will find the odour port on their own and trigger trials by poking their nose into the odour port and breaking the IR beam.
45. During the initial acquisition of the test paradigm, closely monitor the progress of the mice. There is no need to interfere with the automated training unless a mouse exhibit signs of stress or fails to learn the trial structure.
    a. If mice are to be used for odour discrimination tasks, the discrimination accuracy at the end of the pre-training phase should be stable and well over 80% in the last 100 trials to ensure quick acquisition of the go/no-go task.
46. After completion of the pre-training phase the mice progress to the actual go/no-go task with odours being presented in a pseudo-randomized scheme (no more than three consecutive presentations of the same stimulus and equal numbers of rewarded and unrewarded trials within each 100 trial block).
47. Monitor the progress of the mice daily during the odour discrimination and advance the mice to the next odour pair or mixture once the desired performance level has been reached or enough trials for the desired analysis have been acquired.
    a. To determine the discrimination time (DT) at least 150 correct rewarded and unrewarded trials at high performance (≥ 95%) are required for analysis but we highly recommend 500 rewarded and unrewarded trials. Keep the number of trials fairly equal among mice as prolonged training will lead to overtraining of the mice which can artificially decrease the DTs.
    b. Over the course of long test-paradigms it is not uncommon for some mice (mostly animals with above average water intake) to show a drop in performance, stemming from a failure to lick when a rewarded odour is presented.
48. After completion of the required tasks, mice can be sacrificed and used for any necessary post-mortem analysis. The RFID chips can either be discarded or retrieved and disinfected to be re-used.
    a. If post-processing includes fixing the tissue, we recommend to remove the RFID chip prior to the actual fixing step to prevent contamination of the chips with the fixative.

Setup cleaning

49. To clean the cage, pull out the bedding stopper underneath the metal grid so that the soiled bedding can fall through the grid into the disposal bag. Bedding in nests can be left undisturbed.
    a. If clumps of bedding have already formed gently tap on the metal grid to loosen the bedding.
50. Slowly reinsert the bedding stopper taking care not to pinch any tails or toes of mice investigating the floor.
51. Pull out the housing compartment and the bedding stopper simultaneously and refill the bedding from the top.
    a. It is not necessary to manually distribute the bedding in the cage as the mice will eventually do this themselves.
52. To clean the tunnel leading towards the odour port, first make sure no mice are in the tunnel and block access to the tunnel by closing the door.
53. Disconnect the lick port and water delivery system and remove the odour port from the holder.
54. Remove the tunnel from the setup and clean it using only water. Do not use soap, alcohols or disinfectants to clean the cage as any residual traces can be ingested by the animals and any smell of the used products could impair the olfactory testing.
55. Thoroughly dry all parts and reassemble the tunnel and the odour port (follow steps 48 to 53 in reverse order).
    a. Residual water can be ingested by the mice and can lead to reduced activity.

Troubleshooting

Table 1 Troubleshooting information for the respective steps of the protocol.

Step	Problem	Possible Reason	Recommended Solution
35	Odour detector signal is noisy or detects no odour at all.	The detector has a low ionization efficiency for the odour used.	Determine the right odour according to the manufacturer's specification. If none are available try an odour with a high vapour pressure.
		The odour detector is not positioned properly.	Carefully reset the detector position. Refer to Vetter et al. for an in depth analysis of the most common parameters[1].

		The odour concentration is too low.	Gradually increase the odour concentration taking care not to oversaturate the detector.
35	Odour onset and/or odour peak are too high/low.	The tubing leading from the final valve to the odour port is too long/short.	Shorten/lengthen the tubing and re-measure the parameters to validate the success of the changes.
		The air-flow is too fast/slow.	Adjust the air-flow
38	The RFID beams and/or the odour port are blocked with paper.	Animals were provided with paper based bedding or enrichment.	Remove any paper and tissue and provide the animals with wood-based bedding or enrichment.
45	Animal has not performed any trials within 24 hours of starting the training	The animal has not discovered the odour port.	If allowed under the experimental animal license: Manually place the animal in the tunnel alone and lock the door. If the animal does not investigate the odour port on its own consider placing some peanut butter on the odour port to make it more appealing to the animal.
		The RFID chip is not detected by the reader.	If allowed under the experimental animal license: Manually place the animal in the tunnel alone and lock the door. If the RFID chip is indeed not detected when the animal inserts its head in the odour port the chip may not have been placed correctly in the neck fold. This can be fixed by securing the RFID chip in the neck fold using sutures
47	Performance drops when the animal progresses through pre-training stages.	The animal has not understood the newly added conditions for receiving water	Adjust the pre-training protocol by, for example, lowering the additional sampling time until water is delivered or reducing the lick-frequency.<br>Additionally, animals can be manually rewarded with water to prevent loss of motivation.
47	Animal has received less than 1ml water within 24 hours.	The animal has drunk an excessive amount of water during the previous day.	If allowed under the experimental animal license: Check if the water intake in the previous days was higher than usual. If this is the case no intervention is necessary.
		The animal does not understand the current rules for receiving water.	During pre-training consider manually adjusting the training protocol. During odour training manually reward the S+ odour to strengthen the association between the odour and the water reward.

47	Performance of animal during odour training drops for S+ trials.	The animal receives enough water and has lost motivation to actively lick at S+ trials.	If allowed under the experimental animal license: Gradually reduce the amount of water given per correct S+ trial to increase the animal's motivation for responding correctly at every trial.
47	Activity and/or performance drops after cage cleaning.	Animals ingested residual water left from cleaning, reducing the need to perform trials or respond correctly.	Thoroughly dry every cleaned part with high-absorbance paper to remove residual water.
			If allowed under the experimental animal license: Provide the animals with dry food (i.e. oat flakes) to increase thirst after water has already been ingested.

Part 2 – Detailed installation instructions

Connection of pneumatic tubing

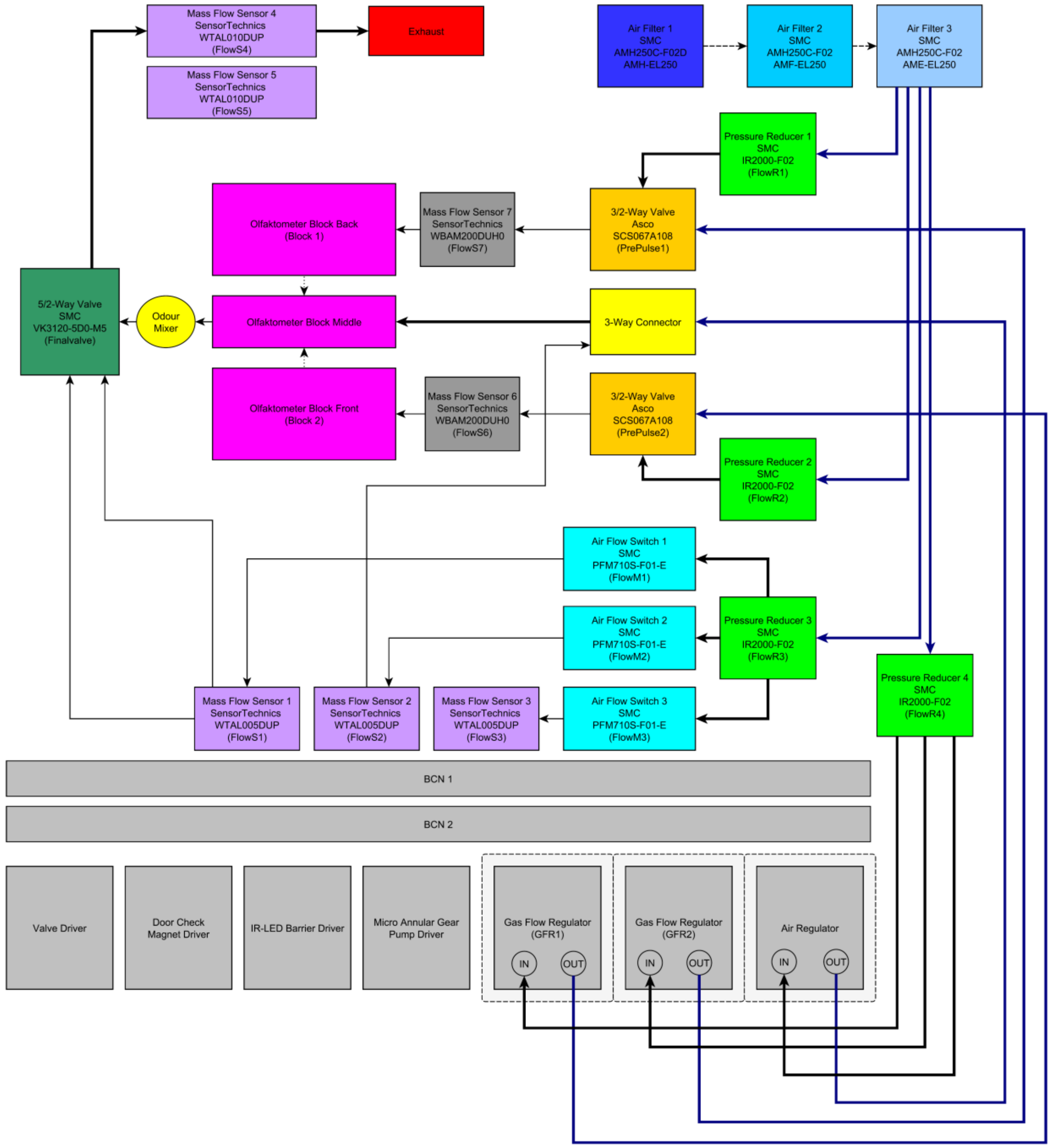

***Supplementary Figure 15 Schematic representation of pneumatic tubing**. Dashed lines between the tree air-filter units denote direct physical connection. Thick lines denote use of large diameter tubing (Festo, # PUN-6x1-SW or # PUN-6x1-BL) while thin lines denote us of smaller diameter tubing (Festo, # PUN-4X0,75-BL). The dotted lines between the three olfactometer blocks denote the connection through the odour reservoirs.*

All tubing connections between air-flow regulators and sensors should be made using dedicated pneumatic tubing as the pressure within the system increases to up to 5 bar during odour training.

Threaded tubing connectors should be additionally secured using Teflon tape to prevent loosening of connections.

Electrical Connections

Physical connection of the DAQ devices

Each of the two PCI I/O devices has two connectors and is physically connected to one of the two BNC connector blocks. Additionally, the PCI device of the first computer is also connected to the valve driver electronics module located in the NIM-Bin power supply unit.

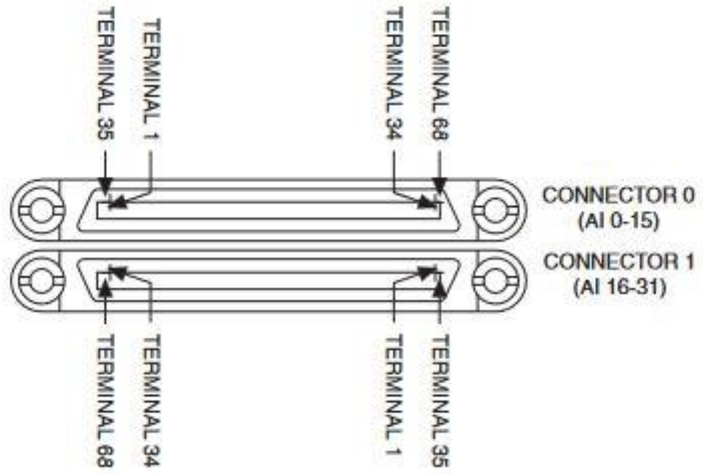

Supplementary Figure 16 Schematic highlighting the pinouts of the PCI device. *(Adapted from „DAQ M Series M Series User Manual“ National Instruments, July 2016)*

PC 1

Of the two connectors of the PCI device, the lower connector (Connector 1) should be connected to the valve driver module while the upper connector (Connector 0) should be connected to the first BNC connector block.

PC2

At the second PCI device, the lower Connector (Connector 0) should be connected to the second BNC connector block.

Wiring of the BNC Boards

All BNC Connectors should be set to Single End (SE) mode. Please refer to the manufacturer's instructions for details on proper grounding and prevention of signal ghosting. Connectors not listed are unconnected.

BNC 1 – BNC Connectors

BNC Label	Connection 1	Connection 2
**AI 0**	BNC 1 on IR Detector Module	AI 0 BNC Connector on BNC 2
**AI 1**	BNC 2 on IR Detector Module	AI 1 BNC Connector on BNC 2
**AI 2**	Output from Darlington Lick Sensor	AI 2 BNC Connector on BNC 2
**AO 0**	Analog-IN on Water Pump Module	

BNC 1 – Spring Block Terminal

Terminal Label	Connection 1	Connection 2	Connection 3	Connection 4

**DGND**	First DGDN on BNC 2 Spring Block Terminal	AI 7 BNC Connector on BNC 2	AI 15 BNC Connector on BNC 2	PFI 0 BNC Connector on BNC 2
**P0 0**	PFI 0 BNC Connector on BNC 2			
**P0 1**	AI 7 BNC Connector on BNC 2			
**P0 4**	P0 4 on BNC 2 Spring Block Terminal			
**P0 5**	PFI 2 on BNC 2 Spring Block Terminal			
**P0 6**	P0 6 on BNC 2 Spring Block Terminal			
**P0 7**	P0 7 on BNC 2 Spring Block Terminal			
**DGND**	Trig. In Signal on Air Regulator Module	Second DGDN on BNC 2 Spring Block Terminal		
**PFI 6**	Trig. In Signal on Air Regulator Module			

BNC 2 – BNC Connectors

BNC Label	Connection 1	Connection 2
**AI 0**	AI 0 BNC Connector on BNC 1	
**AI 1**	AI 1 BNC Connector on BNC 1	
**AI 2**	AI 2 BNC Connector on BNC 1	
**AI 3**	Mass Flow Sensor 7 (Flow S7)	
**AI 4**	Mass Flow Sensor 6 (Flow S6)	
**AI 5**	Gas Flow Regulator 1 (GFR 1)	
**AI 6**	Gas Flow Regulator 2 (GFR 2)	
**AI 7**	P0 1 on BNC 1 Spring Block Terminal	First DGND on BNC 1 Spring Block Terminal
**AI 8**	Mass Flow Sensor 1 (FlowS1)	
**AI 9**	Mass Flow Sensor 2 (FlowS2)	
**AI 10**	Mass Flow Sensor 3 (FlowS3)	
**AI 11**	BNC 3 on IR Detector Module	
**AI 12**	Air Flow Switch 1 (FlowM1)	
**AI 13**	Air Flow Switch 2 (FlowM2)	
**AI 14**	Air Flow Switch 3 (FlowM3)	
**AI 15**	PFI 0 BNC Connector on BNC 2	
**PFI 0**	AI 15 BNC Connector on BNC 2	

BNC 2 – Spring Block Terminal

Terminal Label	Connection 1
**DGND**	First DGDN on BNC 1 Spring Block Terminal
**P0 0**	PFI 0 BNC Connector on BNC 1
**P0 1**	AI 7 BNC Connector on BNC 1
**P0 4**	P0 4 on BNC 1 Spring Block Terminal
**P0 6**	P0 6 on BNC 1 Spring Block Terminal
**P0 7**	P0 7 on BNC 1 Spring Block Terminal
**DGND**	Second DGDN on BNC 1 Spring Block Terminal
**PFI 2**	P0 5 on BNC 1 Spring Block Terminal

Wiring and Settings of the Electronic Driver Modules

Valve Driver

The 25-pin main connector is directly connected to the olfactometer valves. Both valves (i.e. the valves on the outer manifolds as well as the corresponding valves on the central manifold) should be connected to a corresponding 25-pin D-Sub connector as follows:

Pin	Block	Valve	Connector 1
**1**	1 (Back Block)	1	P0 8
**2**	1 (Back Block)	2	P0 9
**3**	1 (Back Block)	4	P0 10
**4**	1 (Back Block)	8	P0 11
**5**	1 (Back Block)	16	P0 12
**6**	1 (Back Block)	32	P0 13
**7**	1 (Back Block)	64	P0 14
**8**	1 (Back Block)	128	P0 15
**9**	1 (Back Block)	256	P0 16
**10**	2 (Front Block)	1	P0 17
**11**	2 (Front Block)	2	P0 18
**12**	2 (Front Block)	4	P0 19
**13**	2 (Front Block)	8	P0 20
**14**	2 (Front Block)	16	P0 21
**15**	2 (Front Block)	32	P0 22
**16**	2 (Front Block)	64	P0 23
**17**	2 (Front Block)	128	P0 24
**18**	2 (Front Block)	256	P0 25
**19**			P0 26
**20**	1 (Back Block)	Odour Pre-Pulse	P0 27
**21**	2 (Front Block)	Odour Pre-Pulse	P0 28
**22**	1 (Back Block)	Relief Valve	P0 29
**23**	2 (Front Block)	Relief Valve	P0 30
**24**		Final Valve	P0 31
**25**			24V DC

Rotary Magnet Controller

The voltage output should be directly connected to the rotary magnet. For direct control of the door, the inputs to the magnet controller can be connected to the BNC outputs of the IR Detector.

IR-LED Barrier Driver

The desired number of individual IR-LEDs should be connected to the IR-LED Driver and the corresponding BNC outputs connected to the two BNC Connector boards. We recommend using two perpendicular IR-LEDs at the odour port to reliably detect the sampling behaviour of the animal.

Micro Annular Gear Pump Driver

The Analog-In BNC connector should be connected to the AO O BNC connector on BNC 1 while the MRZ-Output port should be directly connected to the Micro Annular Gear Pump. The toggle should be set to “Extern” to drive the pump using the Igor software. Setting the toggle to “Poti” allows the user to manually turn on the pump and disperse water independently of the setup control software.

Calibration of the Micro Annular Gear Pump

Prior to starting a behaviour run, the calibration of the water pump should be checked to make sure animals receive the intended amount of water. To do this locate the function “RewardIt”, output the desired amount of water and check if the actually delivered amount of water matches the intended value. If this is not the case, adjust the code of the “RewardIt” function using the comments in the code as guidelines.

Gas Flow Regulator 1

The left toggle switch should be set to the neutral middle position while the right toggle should be set to “Ext PC-In”. The “Durchfluss Signal OUT” BNC connector should be connected to the AI 5 BNC connector on BNC 2, while the “Ext. PC-IN” BNC connector should be connected to the topmost BNC Connector on the Valve driver.

The pneumatics input (left connector) should be connected to the fourth pressure reducer while the output (right connector) should be connected to the Pre-pulse valve for olfactometer block 1 (back block).

Gas Flow Regulator 2

The left toggle switch should be set to the neutral middle position while the right toggle should be set to “Ext PC-In”. The “Durchfluss Signal OUT” BNC connector should be connected to the AI 6 BNC connector on BNC 2, while the “Ext. PC-IN” BNC connector should be connected to the second BNC Connector on the Valve driver.

The pneumatics input (left connector) should be connected to the fourth pressure reducer while the output (right connector) should be connected to the Pre-pulse valve for olfactometer block 2 (back block).

Air Regulator Main Block

The trigger input of the main air regulator (“Trig IN”) should be connected to the PFI 6 port on the spring block terminal of BNC 1. The ground wire for this BNC should be connected to the DGND port of the spring block terminal of BNC 1.

The pneumatics input (left connector, “Pressure IN”) should be connected to the fourth pressure reducer while the output (right connector, “Pressure OUT”) should be connected to the three-way connector supplying the central olfactometer manifold.

Installation of the RFID reader

Install the RFID reader software (“LID650-665”) according to the manufacturer’s instructions and connect the assembled RFID reader. The following settings should be adjusted:

Setting	Value	Software Tabsheet

**RS232/RS485 Baudrate**	9600	Communication (Tab 1)
**Sec. waittime after read**	0.5 sec	Settings (Tab 2)
**Use buzzer during waittime**	ON	Settings (Tab 2)

The buzzer provides an auditory feedback once an RFID signal is detected. We recommend using this feedback during initial training to verify that all RFID chips are properly placed and can be read out. Once all RFID chips have been verified the buzzer can be turned off as not to irritate humans interacting with the setup. We have found no difference in animal behaviour or performance between times with and without the buzzer feedback.

After adjusting, the new settings need to be sent to the RFID reader by pressing the “Set new settings” button. The software must be closed once the Igor-based behaviour software is started as the communication to the reader is otherwise blocked.

Installation of the video surveillance

Software

The remote video surveillance setup is based on multiple Raspberry Pi 2 (Model B) equipped with Raspberry Pi NoIR infrared Cameras (Conrad, #1316978-62 and Adafruit, #3100). The monitoring system was developed by Miguel Mota (https://miguelmota.com) and detailed instructions can be found here:

https://miguelmota.com/blog/raspberry-pi-camera-board-video-streaming/

In order to view all cameras simultaneously in any web-browser the following html-code was written:

```
    <head></head>
    <body>
        <img src="http://IP-RASPBERRY1:9000
/?action=stream" STYLE="position:absolute; TOP:5px; LEFT:5px; WIDTH:640px; HEIGHT:480px" alt="http:
//IP-RASPBERRY1:9000/?action=stream"></img>
        <DIV STYLE="position:absolute; top:450px; left:20px; width:200px; height:15px">
<CENTER><FONT SIZE="+2" COLOR="848484">RaspiCam1</FONT></CENTER>
        </DIV>
    <img src="http://IP-RASPBERRY2:9000
/?action=stream" STYLE="position:absolute; TOP:5px; LEFT:650px; WIDTH:640px; HEIGHT:480px" alt="http:
//IP-RASPBERRY2:9000/?action=stream"></img>
        <DIV STYLE="position:absolute; top:450px; left:660px; width:200px; height:15px">
<CENTER><FONT SIZE="+2" COLOR="848484">RaspiCam2</FONT></CENTER>
        </DIV>
    <img src="http://IP-RASPBERRY3:9000
/?action=stream" STYLE="position:absolute; TOP:490px; LEFT:5px; WIDTH:640px; HEIGHT:480px" alt="http:
//IP-RASPBERRY3:9000/?action=stream"></img>
        <DIV STYLE="position:absolute; top:930px; left:20px; width:200px; height:15px">
<CENTER><FONT SIZE="+2" COLOR="848484">RaspiCam3</FONT></CENTER>
        </DIV>
    <img src="http://IP-RASPBERRY4:9000
/?action=stream" STYLE="position:absolute; TOP:490px; LEFT:650px; WIDTH:640px; HEIGHT:480px" alt="http:
//IP-RASPBERRY4:9000/?action=stream"></img>
        <DIV STYLE="position:absolute; top:930px; left:660px; width:200px; height:15px">
<CENTER><FONT SIZE="+2" COLOR="848484">RaspiCam4</FONT></CENTER>
        </DIV>
</body>

</html>
```

Supplementary Figure 17 HTML-Code used to simultaneously view four Raspberry Pi based cameras in a web-browser. *The placeholders „IP-RASPBERRY“ 1 to 4 need to be replaced with the IP-address of the Raspberry Pis used.*

Please be advised that the IP-addresses of the individual Raspberry Pis need to be added and that the rotation of the images may need to be adjusted depending on the individual placement of the cameras in the setup.

For mounting the cameras, 3D printed cases with moveable camera mounts were used. The specifications can be found here:

https://www.thingiverse.com/thing:685074

The cases can either be firmly fixed directly to the PVC plate above the housing cage or attached using magnets to allow for changes and removal of the systems if necessary.

Installation of temperature and humidity logging

For automated logging of temperature and humidity in the animal housing cage, one of the Raspberry Pis (Conrad, #1316978-62) was additionally fitted with a temperature and humidity sensor (Adafruit, #385) according to the following guide:

https://learn.adafruit.com/dht-humidity-sensing-on-raspberry-pi-with-gdocs-logging/overview

Part 3 - BEAST Software

Introduction

This short guide to the **B**ehavioural **E**valuation of **A**nimal **S**ensory **T**esting (BEAST) software assumes the user is familiar with the basics of Igor Pro 6 (Wavemetrics) as well as fundamental programming concepts. In particular, the software relies heavily on interconnected custom written functions and coded referencing of waves.

For first time Igor users we highly recommend completing at least the dedicated Igor tutorials available from Wavemetrics, including the “Guided Tour”.

Principle

The setup itself uses two computers running two independent instances of Igor 6, with one controlling the actual training (PC 1) and the other collecting data from the different sensors (PC2).

Installation of Igor Pro 6

Igor Pro 6 should be installed according to the manufacturer’s instructions. Additionally, the following packages are required:

- Igor NIDAQ Tools MX (available from Wavemetrics)
- Igor XOP Toolkit (available from Wavemetrics)

Recommended changes to the default parameters

Some settings should be adjusted to ensure proper unsupervised functionality of the software:

- The Command History needs to be limited (i.e. to 1000 lines) as an unlimited history inflates the file size and will cause Igor Pro 6 to crash once the file size exceeds 4 GB. While newer versions of Igor Pro do not have a 4GB file size limit, proper functionality of the program should be closely monitored if the Command History is used.
- The Debugger and specifically the “Debug on Error” option should be switched off as these will cause the software to stop and await active user interaction once an error is detected (mostly a missing wave reference). If this option is used the system needs to be closely monitored to prevent animals from developing aberrant behavioural responses due to an unresponsive setup.

Installation of DAQ devices

The setup is controlled through dedicated input/output devices (National Instruments, #779068-01), which are directly installed at the computers PCI slots. Please closely follow to the manufacturer’s instructions and install the recommended most-current drivers as specified by the manufacturer.

During installation of accompanying software, the user is asked to supply a name for the DAQ device. As this exact name is needed as a hardware reference in the Igor code (see *Hardware Referencing*) we highly recommend using “Dev1” to avoid large-scale adjustments to the code. After completed installation, the device name can be changed using the “National Instruments MAX” Software.

Hardware Referencing

Any hardware peripherals need to be correctly referenced in the Igor code to ensure full functionality of the setup. The following two devices are currently referenced in the code and may need to be adjusted depending on the individual installations:

Hardware Device	Manufacturer	Current Reference	Comments
**PCI Card**	National Instruments	Dev1	Must be identical to name specified during installation of the card (see *Installation of DAQ devices*)
**RFID Reader**	Euro I.D.	COM6	Reference must be adjusted to correspond to COM-port on setup (see *Hardware Referencing*)

Any connection to the two BNC-Boards (National Instruments, #779556-01) should be made exactly as specified in the chapter Electrical Connections as these connections are also referenced individually in the code.

Networking of the two PCs

If two independent PCs are used, the two should be connected either directly or through a shared network connection. We highly recommend a direct connection or a limited local network as any connection time-outs can affect the performance of the behaviour software.

Behaviour Software (PC 1)

Principle

This Igor code runs on the first PC and is the actual software controlling the behaviour training and running any data analysis of the acquired data. The code can be found in accompanying online repository (https://github.com/AutomatedOlfactoryBehaviour/Beast).

Main window

The user interface of the main behaviour software has three main components: the date and time panel at the top, a summary table showing the main parameters of the initiated trials and the main control panel on the right-hand side of the screen.

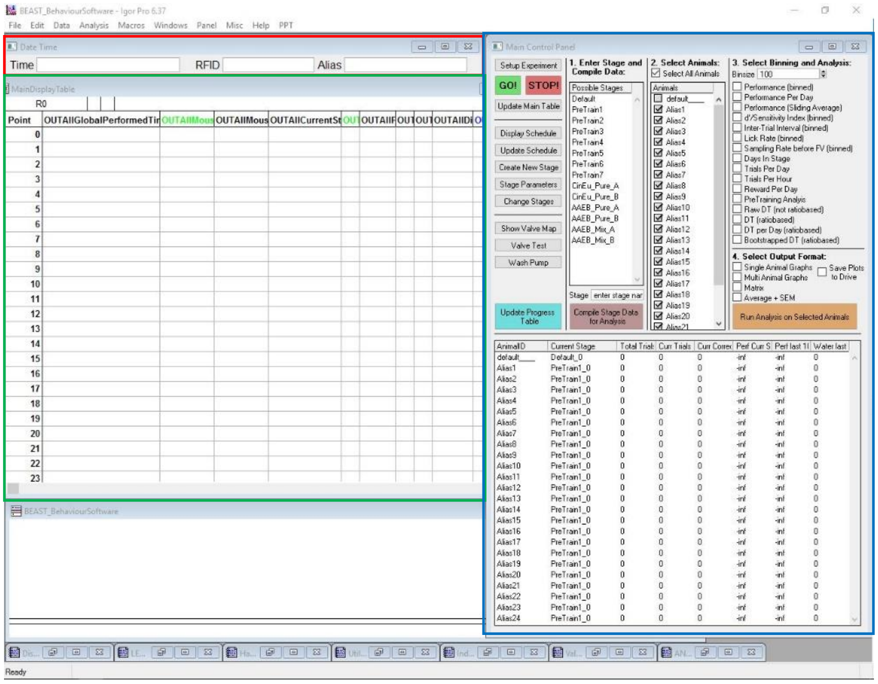

Supplementary Figure 18 User Interface of the main behaviour software. *The date and time panel (red) displays the current date and time and (in the event of a trial) the ID and the Alias of the animal. The summary table (green) shows the main parameters of the trials while the main control panel (blue) served to control the software.*

Starting a new behaviour run

To start a new behaviour run, press the “Display Schedule” button. This opens a new table containing the individual training protocols per animal.

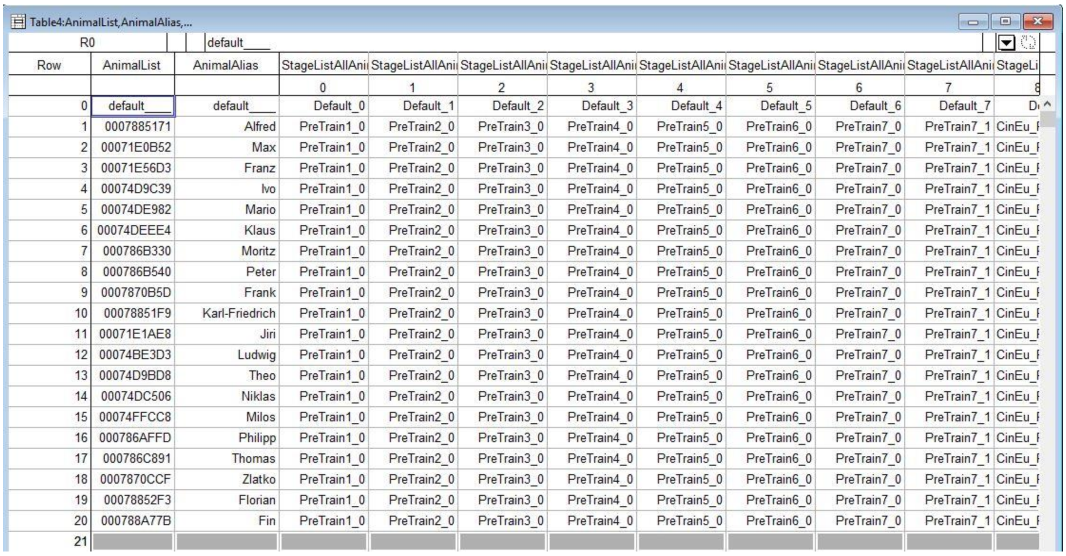

Table4:AnimalList,AnimalAlias,...

R0 | default____

Row	AnimalList	AnimalAlias	StageListAllAni 0	StageListAllAni 1	StageListAllAni 2	StageListAllAni 3	StageListAllAni 4	StageListAllAni 5	StageListAllAni 6	StageListAllAni 7	StageLi 8
0	default____	default____	Default_0	Default_1	Default_2	Default_3	Default_4	Default_5	Default_6	Default_7	D
1	0007885171	Alfred	PreTrain1_0	PreTrain2_0	PreTrain3_0	PreTrain4_0	PreTrain5_0	PreTrain6_0	PreTrain7_0	PreTrain7_1	CinEu_
2	00071E0B52	Max	PreTrain1_0	PreTrain2_0	PreTrain3_0	PreTrain4_0	PreTrain5_0	PreTrain6_0	PreTrain7_0	PreTrain7_1	CinEu_
3	00071E56D3	Franz	PreTrain1_0	PreTrain2_0	PreTrain3_0	PreTrain4_0	PreTrain5_0	PreTrain6_0	PreTrain7_0	PreTrain7_1	CinEu_
4	00074D9C39	Ivo	PreTrain1_0	PreTrain2_0	PreTrain3_0	PreTrain4_0	PreTrain5_0	PreTrain6_0	PreTrain7_0	PreTrain7_1	CinEu_
5	00074DE982	Mario	PreTrain1_0	PreTrain2_0	PreTrain3_0	PreTrain4_0	PreTrain5_0	PreTrain6_0	PreTrain7_0	PreTrain7_1	CinEu_
6	00074DEEE4	Klaus	PreTrain1_0	PreTrain2_0	PreTrain3_0	PreTrain4_0	PreTrain5_0	PreTrain6_0	PreTrain7_0	PreTrain7_1	CinEu_
7	000786B330	Moritz	PreTrain1_0	PreTrain2_0	PreTrain3_0	PreTrain4_0	PreTrain5_0	PreTrain6_0	PreTrain7_0	PreTrain7_1	CinEu_
8	000786B540	Peter	PreTrain1_0	PreTrain2_0	PreTrain3_0	PreTrain4_0	PreTrain5_0	PreTrain6_0	PreTrain7_0	PreTrain7_1	CinEu_
9	0007870B5D	Frank	PreTrain1_0	PreTrain2_0	PreTrain3_0	PreTrain4_0	PreTrain5_0	PreTrain6_0	PreTrain7_0	PreTrain7_1	CinEu_
10	00078851F9	Karl-Friedrich	PreTrain1_0	PreTrain2_0	PreTrain3_0	PreTrain4_0	PreTrain5_0	PreTrain6_0	PreTrain7_0	PreTrain7_1	CinEu_
11	00071E1AE8	Jiri	PreTrain1_0	PreTrain2_0	PreTrain3_0	PreTrain4_0	PreTrain5_0	PreTrain6_0	PreTrain7_0	PreTrain7_1	CinEu_
12	00074BE3D3	Ludwig	PreTrain1_0	PreTrain2_0	PreTrain3_0	PreTrain4_0	PreTrain5_0	PreTrain6_0	PreTrain7_0	PreTrain7_1	CinEu_
13	00074D9BD8	Theo	PreTrain1_0	PreTrain2_0	PreTrain3_0	PreTrain4_0	PreTrain5_0	PreTrain6_0	PreTrain7_0	PreTrain7_1	CinEu_
14	00074DC506	Niklas	PreTrain1_0	PreTrain2_0	PreTrain3_0	PreTrain4_0	PreTrain5_0	PreTrain6_0	PreTrain7_0	PreTrain7_1	CinEu_
15	00074FFCC8	Milos	PreTrain1_0	PreTrain2_0	PreTrain3_0	PreTrain4_0	PreTrain5_0	PreTrain6_0	PreTrain7_0	PreTrain7_1	CinEu_
16	000786AFFD	Philipp	PreTrain1_0	PreTrain2_0	PreTrain3_0	PreTrain4_0	PreTrain5_0	PreTrain6_0	PreTrain7_0	PreTrain7_1	CinEu_
17	000786C891	Thomas	PreTrain1_0	PreTrain2_0	PreTrain3_0	PreTrain4_0	PreTrain5_0	PreTrain6_0	PreTrain7_0	PreTrain7_1	CinEu_
18	0007870CCF	Zlatko	PreTrain1_0	PreTrain2_0	PreTrain3_0	PreTrain4_0	PreTrain5_0	PreTrain6_0	PreTrain7_0	PreTrain7_1	CinEu_
19	00078852F3	Florian	PreTrain1_0	PreTrain2_0	PreTrain3_0	PreTrain4_0	PreTrain5_0	PreTrain6_0	PreTrain7_0	PreTrain7_1	CinEu_
20	000788A77B	Fin	PreTrain1_0	PreTrain2_0	PreTrain3_0	PreTrain4_0	PreTrain5_0	PreTrain6_0	PreTrain7_0	PreTrain7_1	CinEu_
21											

Supplementary Figure 19 Example training schedule for a cohort of 20 animals. *The training schedule must include a default animal and training sequence which is executed in case no RFID tag is detected.*

Simply enter the animal ID (= RFID tag) into the first column, an Alias (i.e. a name) into the second column and the desired training sequence in the subsequent columns. This table must include a default-animal (“default____”) and a default-training schedule which is executed in case no RFID chip was detected. Also make sure to leave the last column of the table blank due to technical reasons.

After the animals and training parameters have been specified, close the table and press the “Setup Experiment” button. This will wipe any pre-existing data and generate all waves and folders needed during the training. To begin the training, press the “GO!” button. To pause the training, press the “STOP!” button.

Naming conventions for training schedules

The naming of the individual stages follows a simple convention, where the name of the training stage is followed by an underscore and a single-digit suffix. The name of the training stage has to be identical to the title of the corresponding subfolder in the Templates folder (*root:templates:*) and denotes the actual training program that is to be run. The single-digit suffix can be alphanumerical and is used to allow repetitions of different training stages. For example, “PreTrain7_0” and “PreTrain7_1” both refer to the seventh pre-training phase and hence both follow the same training parameters.

When animals are trained to distinguish two different odours, it is good practice to split the animals in two groups to use both odours as rewarded and unrewarded odours, respectively. This serves as a control for potential native aversion or attraction of the animals to one of the odours which could affect the discrimination abilities. In the pre-defined odour stages in the Templates folder (*root:templates:*), these groups are specified by the addition of the infix “_A” or “_B” to the stage name (i.e. “AAEB_Pure_A” and “AAEB_Pure_B”). Note that this infix is part of the stage name and used in addition to the suffix denoting repetitions of the training stage.

Adjusting the training schedule after initial initialization of the training

After the first start of the training, the schedule for each animal can be adjusted by making changes to the table. However, changes can only be made to upcoming training stages. Changes to the current or even previous stages will not change the future training schedule but may lead to loss of data, i.e. if a previously used training stage is repeated without adjusting the suffix to reflect this repetition.

After changes to the training table have been made, it is necessary to re-initialize the training schedule of the animals. This is done by pressing the “Update Stages” button and re-initialization is confirmed through a prompt in the command line.

Quick monitoring

To quickly monitor an animal’s progress, press the “Current Progress for All Animals” button. This will update the table underneath it and display the most important key facts for the current stage of each animal. The values include total number of trials, number of correct trials, the performance in the entire stage as well as across the last 100 trials and the water intake within the last 100 trials. Since this quick analysis option does not take into account trials where the pre-sampling threshold was not reached and hence no odour was presented, this analysis may understate the actual performance of an animal.

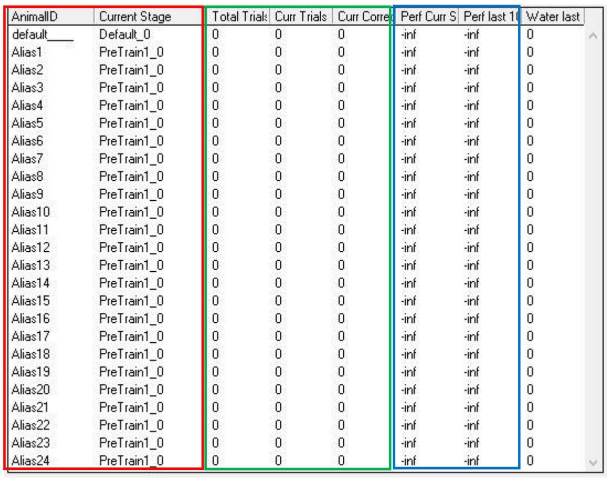

AnimalID	Current Stage	Total Trial	Curr Trials	Curr Correc	Perf Curr S	Perf last 1(	Water last
default____	Default_0	0	0	0	-inf	-inf	0
Alias1	PreTrain1_0	0	0	0	-inf	-inf	0
Alias2	PreTrain1_0	0	0	0	-inf	-inf	0
Alias3	PreTrain1_0	0	0	0	-inf	-inf	0
Alias4	PreTrain1_0	0	0	0	-inf	-inf	0
Alias5	PreTrain1_0	0	0	0	-inf	-inf	0
Alias6	PreTrain1_0	0	0	0	-inf	-inf	0
Alias7	PreTrain1_0	0	0	0	-inf	-inf	0
Alias8	PreTrain1_0	0	0	0	-inf	-inf	0
Alias9	PreTrain1_0	0	0	0	-inf	-inf	0
Alias10	PreTrain1_0	0	0	0	-inf	-inf	0
Alias11	PreTrain1_0	0	0	0	-inf	-inf	0
Alias12	PreTrain1_0	0	0	0	-inf	-inf	0
Alias13	PreTrain1_0	0	0	0	-inf	-inf	0
Alias14	PreTrain1_0	0	0	0	-inf	-inf	0
Alias15	PreTrain1_0	0	0	0	-inf	-inf	0
Alias16	PreTrain1_0	0	0	0	-inf	-inf	0
Alias17	PreTrain1_0	0	0	0	-inf	-inf	0
Alias18	PreTrain1_0	0	0	0	-inf	-inf	0
Alias19	PreTrain1_0	0	0	0	-inf	-inf	0
Alias20	PreTrain1_0	0	0	0	-inf	-inf	0
Alias21	PreTrain1_0	0	0	0	-inf	-inf	0
Alias22	PreTrain1_0	0	0	0	-inf	-inf	0
Alias23	PreTrain1_0	0	0	0	-inf	-inf	0
Alias24	PreTrain1_0	0	0	0	-inf	-inf	0

Supplementary Figure 20 Quick monitoring of all animals through the progress table. *The table lists each animal by alias (not by RFID code) and shows their current training stage (red). Progress can be assessed through the different numbers of trials (green) or the performance (blue).*

Built-in analysis options

For a more detailed analysis, the built in analysis options can be used which can be found in the top right half of the Main Control Panel:

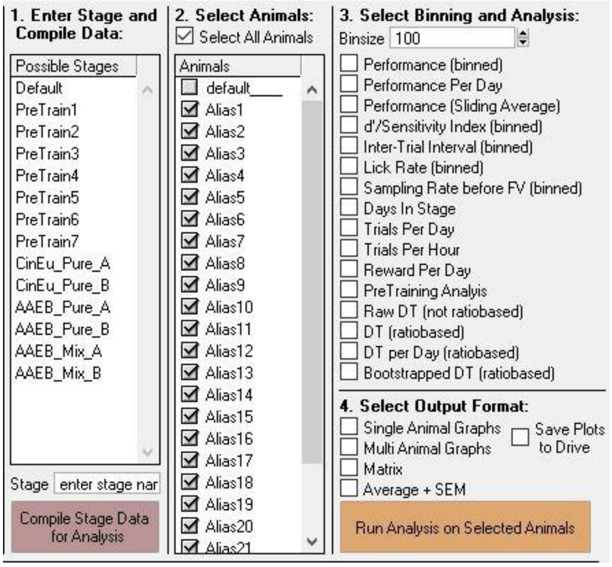

Supplementary Figure 21 Built-in analysis options. *Through selection of the individual animals and analysis options, the progress of the animals can be directly assessed using the BEAST software. All analysis options can be selected independently or in any arbitrary combination. DT = discrimination time.*

Apart from the different analysis options, the list boxes also list all animals in the current behaviour run as well as all different training stages assigned throughout the current behaviour run across all animals.

To run the analysis, first specify the stage that should be analysed. The stage name should utilize the standard wildcard syntax to analyse both cohorts of animals (i.e. “AAEB_Pure_A” and “AAEB_Pure_B” can be analysed jointly by entering “AAEB_Pure*”). Then press the “Compile Stage Data for Analysis” button to collect the data for this stage from all animals. Once all data has been collected, the command line will display “Finished compiling data. Ready to run analysis” to signal that the analysis can proceed.

Any analysis or combination of multiple analyses can be selected using the respective checkboxes. Similarly, the analysis can be restricted to specific animals or groups of animals. The data can be presented either as graphs for all selected animals ("Multi Animal Graphs") or as individual graphs per animal ("Single Animal Graphs"). If the underlying data is needed, the output includes matrices showing the individual values per animal ("Matrix") or tables containing the averages of all selected animals ("Average + SEM").

Advanced options

The main control panel offers additional features to quickly adjust the training schedule of the animals or for routine maintenance of the setup. Some of the more complex features involving new user interfaces are explained below.

Creating new training stages

To generate new training stages for odour discrimination training of two different odours, press the "Create Stage" button. This opens up a new GUI.

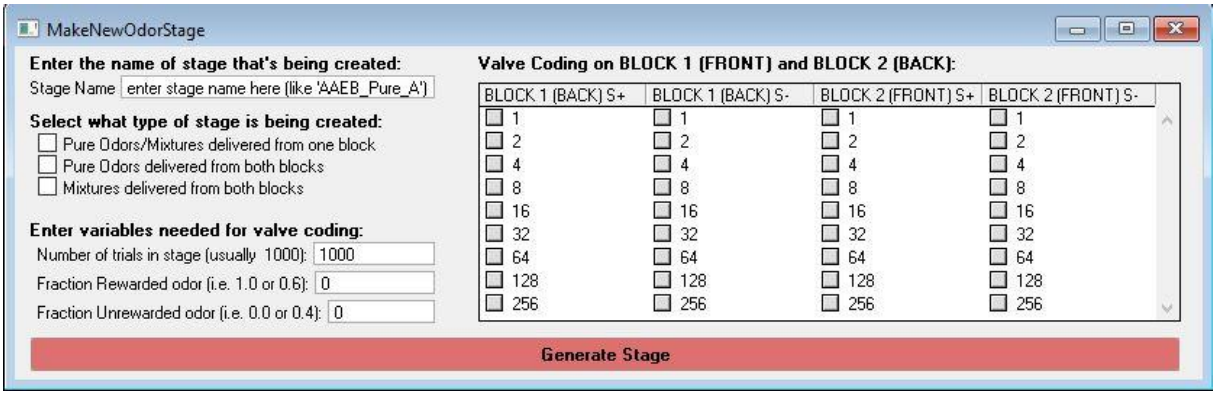

Supplementary Figure 22 The graphical user interface to aide generation of new training stage templates. *The user can specify the type if the training stage (i.e. presentation of pure or mixed odours) and input the coding for the individual odour valves.*

Here, the user can use the various input boxes and checkboxes to set the parameters for creating the stage. Any parameters not set via the GUI (i.e. amount of water reward per trial or the ITI) are hardcoded but can be adjusted after the new stage has created.

Quick changes to training stage templates

To adjust the baseline parameters for the stage templates, press the "Stage Parameters" button. This opens up a new GUI to select the stages that need to be changed.

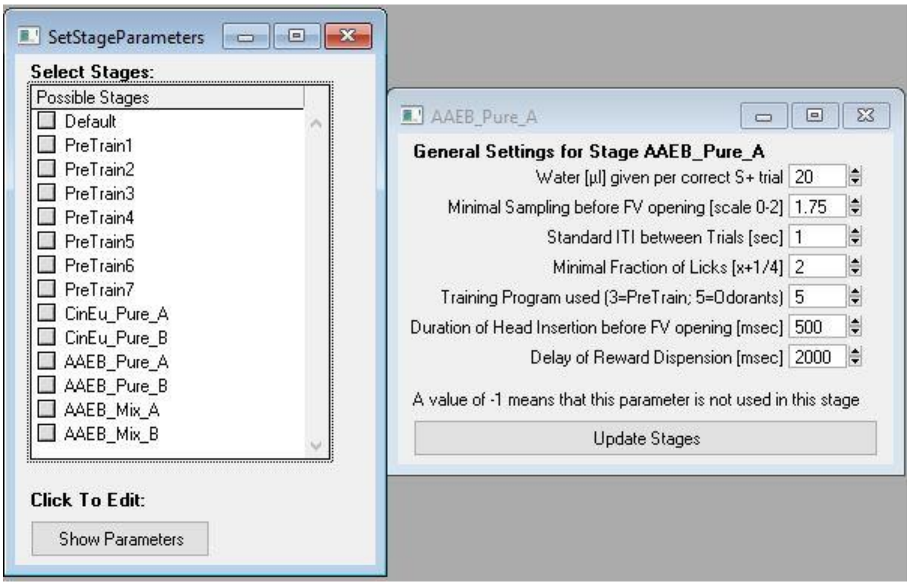

Supplementary Figure 23 The graphical user interface to aide changing of important stage parameters. *The initial GUI (left) lists all stages used in the training schedule. Upon selection of a given stage, a second GUI (right) is created, displaying the most important parameters for this stage.*

This initial GUI lists all stages used throughout the current behaviour run and allows the user to select the stages to edit the parameters. After clicking the "Show Parameters" button, new GUIs are opened (one for each selected stage) which can be used to change the most important training parameters of each stage. The changes affect the templates for the stages located in the *root:templates:* folder and hence only affect future instances of these stages.

Manually moving animals to specific training stages

To manually advance an animal to a different training stage, press the "Change Stages" button. This opens up a new GUI showing both the animals (identified by RFID code and Alias), as well as their current training stage (identified by name and stage number).

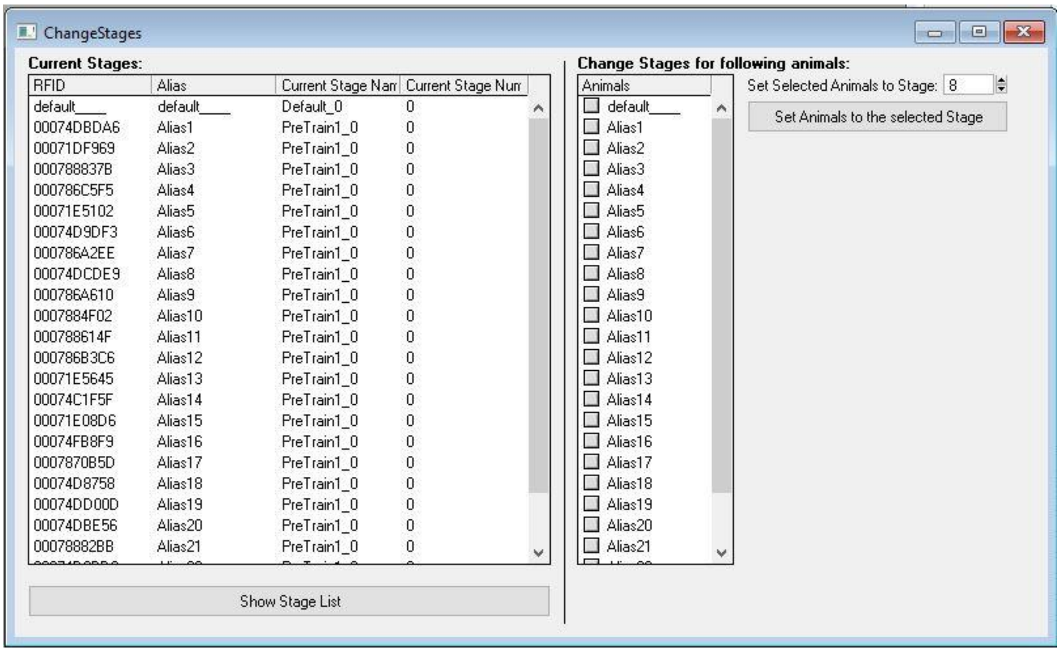

Supplementary Figure 24 The graphical user interface to manually change the training stage of any given animal. *The left table lists the animals and the checkboxes on the right allow animals to be selected and moved to different stages.*

To change the training stage, select the animals that should be changed, enter the number of the training stage the animals should be moved to and press “Set Animals to selected Stage” for the change to take effect. If the number of the new stage is not known, the user can press the “Show Stage List” button which will open the training protocol for all animals. The number of the stage is identical to the column number the stage is found in.

Switching locations of odour reservoirs

During odour training, it is good practice to change the location of the different odour reservoirs on the olfactometer to ensure that the animals do not use cues other than the odours to solve the discrimination tasks. To do this without making large scale alterations to the pre-defined training protocols, press the “Show Valve Map” button. Again, this opens up a new GUI to note the new locations.

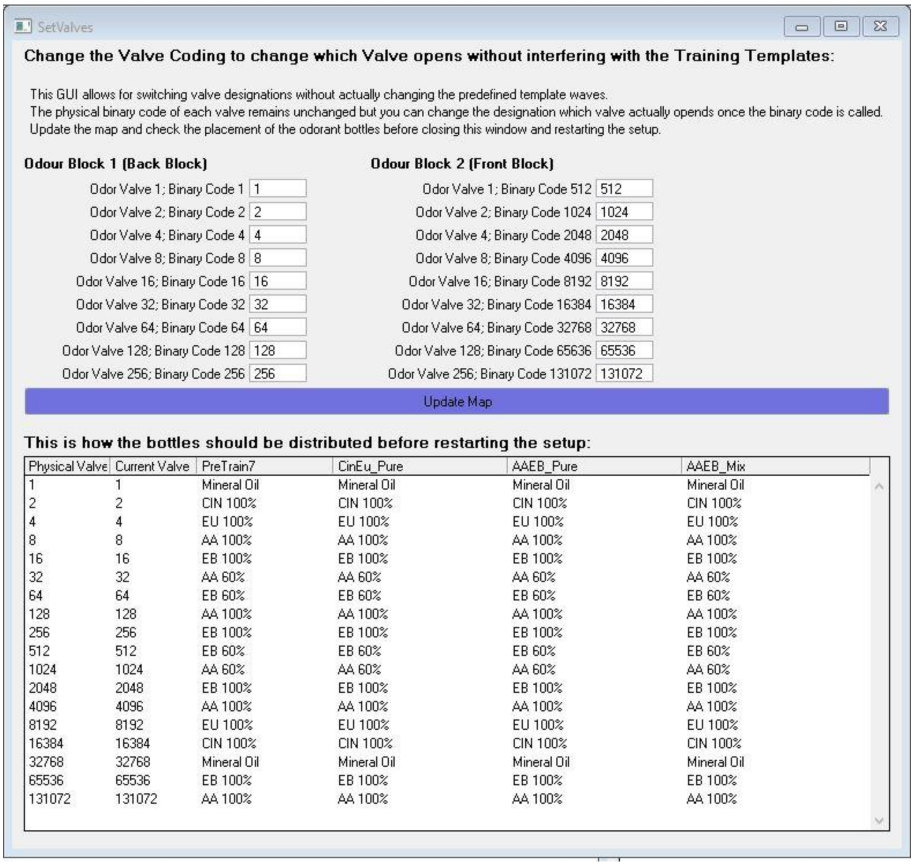

Supplementary Figure 25 The graphical user interface to aid switching of odour reservoirs to different valves. *The input boxes in the top half can be used to input the new location of the odour reservoirs while the table in the bottom half can be used to visualize the changes.*

In the top half of the GUI, the user can enter the new location of the odour reservoirs by entering the binary valve designation of the swapped valves in the input fields. I.e. if the odour bottles from valve 2 on the front block (binary designation 2) and valve 8 on the back block (binary designation 4096) are swapped, the user has to input the number 4096 in the input field for valve 2 on the front block and the number 2 in the input field for valve 8 on the back block.

The bottom half of the GUI shows the distribution of the odour reservoirs on the individual valves for the most commonly used pre-defined stages found in the *root:templates:* folder. This table can be updated by pressing the “Update Map” button above it.

Users should verify correct assignment of the valves by observing opening and closing of the valves (as visible by their indicator LED).

Technical considerations for the calculation of discrimination times

One major property for investigating olfactory processing is the so-called discrimination time (DT), i.e. the fastest reaction time point to a given odour stimulus. The method previously described by Abraham et al.[3] proposed the use of at least 150 rewarded and unrewarded trials with high (≥95%) performance to accurately determine the DT. As mice in our automated setting typically generate far higher numbers of trials over prolonged times, we modified the calculation algorithm to account for this fact. Mainly, the performance is not calculated in bins of trials but individually for each trial using a sliding average over the previous 100 trials. Subsequently, every correct trial with an accuracy ≥95% is included into the trial pool available for calculating the discrimination time. Lastly, the longer training durations are accounted for by randomly subsampling 500 trials (each rewarded and unrewarded) to calculate the discrimination time. This is repeated 10000 times and the final discrimination time for each animal is the average of all these repetitions.

As the underlying test-statistic for calculating the DT is a point-wise t-test, both the threshold p-value as well as the number of trials included in the analysis can affect the resulting discrimination time. The threshold p-value is used to determine at which time-point the sampling pattern for the unrewarded trials becomes significantly different from the sampling pattern of the rewarded trials. Or in other words, the time-point at which the mouse has determined that the odour will not lead to a water reward, and hence, has removed its head from the odour port. In the overwhelming majority of cases, the mice will show the prototypical sampling patterns (**Supplementary Figure 26**a1) described by Abraham et al. and others (see i.e. [3,7–10]) in which case the change in p-values will be rapid and easily detectable (**Supplementary Figure 26**a2). In these instances, changing the threshold from 0.05 to a more stringent value of 0.001 will result in a slightly increased discrimination time. However, if the sampling behaviour does not conform to this norm (**Supplementary Figure 26**b1, c1), a less stringent threshold may fail to detect a discrimination time (**Supplementary Figure 26**b2) or result in an unrealistically low discrimination time (**Supplementary Figure 26**c2).

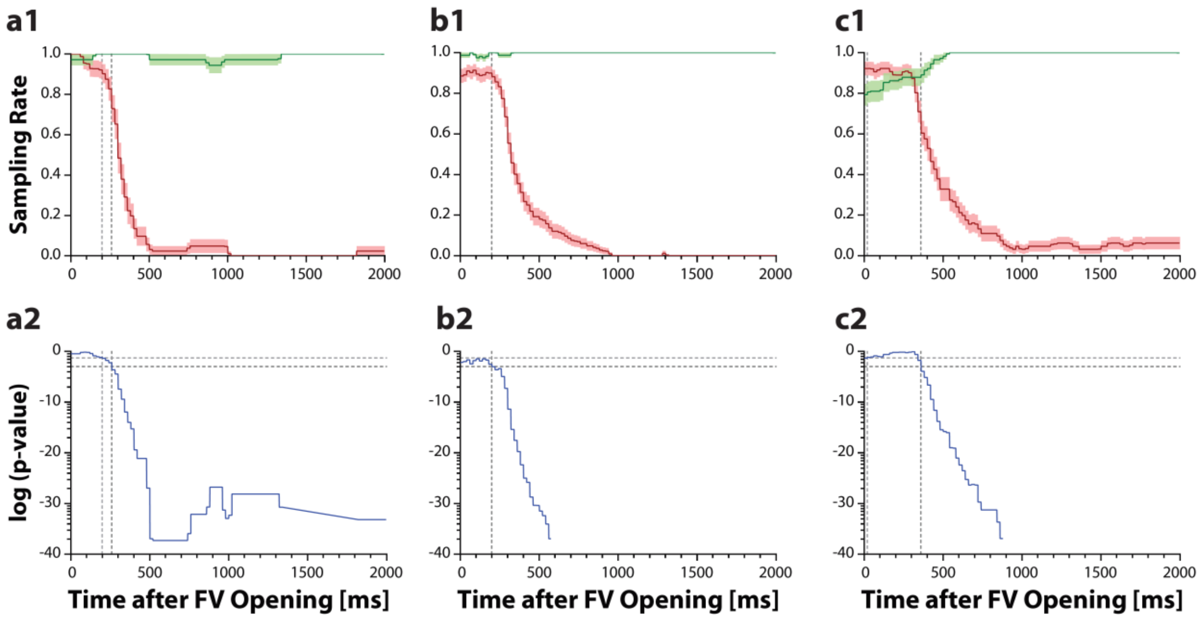

Supplementary Figure 26 Effect of varying threshold p-Values during determination of discrimination times. Representative examples of the average sampling behaviour of different animals for rewarded (green) and unrewarded (red) trials **(a1-c1)**.

While animals will remain in the odour port for rewarded trials, they will retract their heads for unrewarded trials. Pointwise comparison of these curves using a t-Test **(a2-c2)** is used to determine the discrimination time, defined as the time-point at which resulting p-value (blue) crosses a defined threshold. **(a1, a2)** Use of a threshold p-Value of 0.05 (light grey dotted lines) compared to a p-Value of 0.001 (dark grey dotted lines) yields comparable discrimination times. **(b1, b2)** Due to lower overall sampling behaviour for unrewarded trials, a threshold of 0.05 fails to detect a discrimination time while a threshold of 0.001 still detects the difference between the sampling curves. **(c1, c2)** A threshold of 0.05 results in an unrealistically low discrimination time while a threshold of 0.001 still yields a realistic value. For data shown in a1-c1, lines denote the mean while the shaded area denotes the SEM.

While these cases can be detected by manual inspection, any adjustments would need to be made manually by the experimenter (possibly introducing errors) and would significantly increase the workload if large cohorts of animals are to be tested. Hence, we decided to only use the more stringent threshold of 0.001 and validate this new algorithm by using it to re-analyse a dataset generated by Nunes et al. using manual behaviour setups[11]. As expected, using the identical threshold value of 0.05 resulted in few changes, while a more stringent value of 0.001 resulted in increased discrimination times (**Supplementary Figure 27**a).

Ultimately, this modified algorithm was used to analyse the effect of increasing stimulus complexity on the reaction time-point. As described previously by us and others, a more complex odour stimulus results in slower reaction times of the mice[3,11–13]. Using this automated setup we could clearly replicate the increase in reaction times observed for binary mixtures of two odours compared to the reaction times observed for unmixed pure odours (**Supplementary Figure 27**b). Moreover, even despite the overall increase in the discrimination times due to the more stringent threshold p-value, the difference in discrimination times of 80.2 ms is close to the value previously determined using manual testing (see Abraham et al., 2010; ΔDT=88.6 ms).

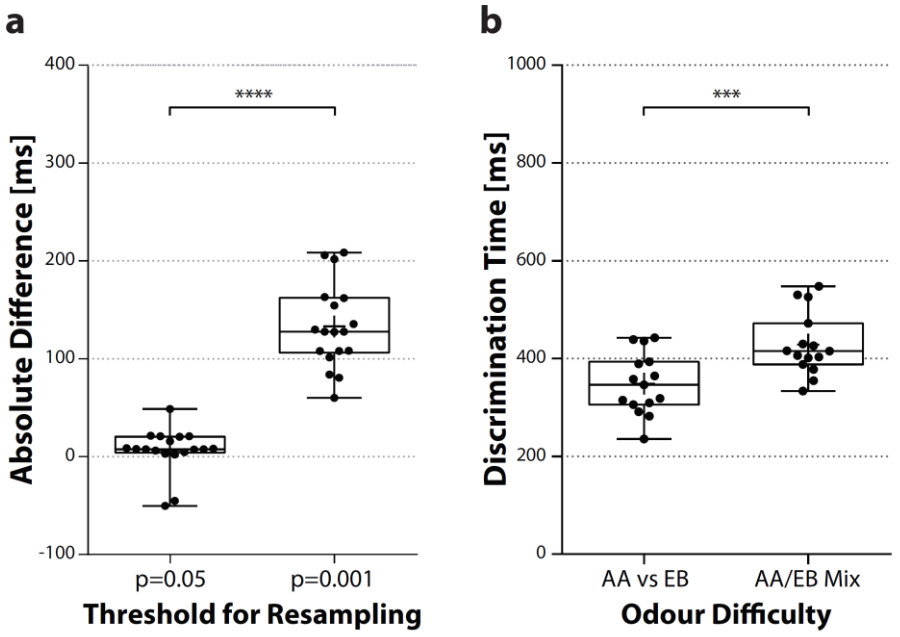

Supplementary Figure 27 Factors affecting the determination of discrimination times. (a) *Absolute differences of discrimination times determined with varying threshold p-Values using novel resampling algorithm compared to the method previously described by Abraham et al.[3]. While using the same threshold p-Value of 0.05 results in largely unchanged discrimination times, these times increase substantially when employing a threshold of 0.001 (p<0.0001; n=18)* ***(b)*** *More complex odour stimuli, like binary mixtures of pure odours, lead to significantly increased discrimination times. Despite the overall increase of discrimination time values due to the use of a more stringent threshold p-Value (p=0.0004; n=18), the absolute difference of 80.2 ms is close to the increase of 88.6 ms reported by Abraham et al.[14]. Whiskers range from min. to max. values. The line denotes median, the cross denotes the mean and black dots represent individual animals.*

Sensor Acquisition Software (PC 2)

Principle

This Igor code runs on the second PC and is dedicated to recording the data of all air-flow and pressure sensors as well as the lick response and the sampling rate of the animal. Start of the recording is triggered once a trial is initiated on the first PC and only ends once the animal has fully retracted its head from the odour port.

To correctly assign each file to a specific trial and animal, the current trial number as well as the animal-ID are stored as text-variables by the first PC upon initiation of a trial. These variables are directly loaded by the sensor acquisition code and used when saving the recorded data (see *Data output*).

User interface

As the user interface only serves to monitor data acquisition it is intentionally minimalistic. Data acquisition is initiated by pressing the "Start Data Acquisition" button. This results in two prompts to open, allowing the user to specify the folder in which the recorded data should be saved as well as the folder where the shared variables saved by the first PC are located.

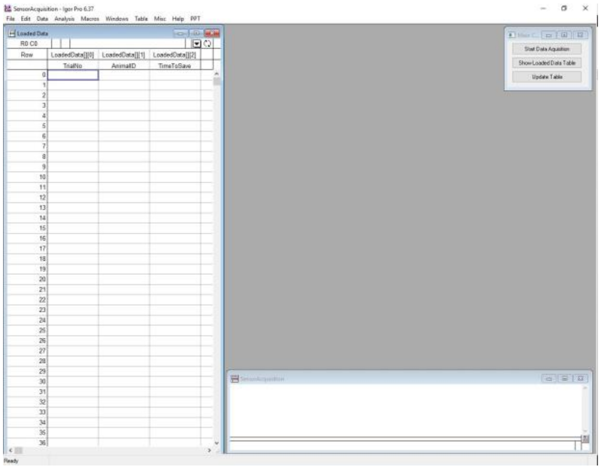

Supplementary Figure 28 User Interface of the Sensor Acquisition Software. *The table on the left displays the trial numbers along with the associated animal and the duration necessary to save the data. The panel in the top right-hand corner can be used to control the acquisition software*

For monitoring the recording progress, the table in the right hand side of the frame saves the number of the current trial as well as the associated animal ID. As an internal control it also saves the time (in seconds) that were needed to stop the recording and save the data to a file. In case the table is accidentally closed, it can be reopened by pressing the "Show Loaded Data Table" button. Lastly, by pressing the "Update Table" button, the table is shifted to show the five most recent trials recorded.

Required user input

To ensure proper functionality, the PCI Card must be referenced correctly (see *"Hardware Referencing"*). If a device name different from "Dev1" was used during installation this needs to be adjusted in the code accordingly.

Further, the location of the shared variables stored by the first computer must be provided. In the current setup, the first PC saves the variables to a shared folder on its own HDD. The second PC running

the Sensor Acquisition Software is granted access to this shared folder. This setup ensures that the variables can still be saved even in the unlikely event that the second computer crashed or is unresponsive. We recommend directly connecting the two PCs and mapping the shared folder on the first computer as a network drive to keep the name of the path short and free of putative escape characters to reduce the potential of incorrectly assigned folders.

Data output

At the end of each recording, the data is compiled into a two-dimensional wave and saved as an *.ibw file using the following file-name convention:

*TrialNumber_AnimalID_*Trialdata

Inclusion of both trial-number and animal-ID provides additional safety from incorrectly assigning a trial to a specific animal.

While it is possible to directly store these files on a server, this requires a constant stable network connection. Any connection time-outs may result in loss of data or crashes of the software. We therefore strongly recommend saving the data locally (if possible on a second drive and not the drive running the operating system of the computer) and transferring it later on (i.e. during cage-cleaning intervals or after completion of the behaviour run).

Potential modifications to conserve storage space

Currently, the software is configured to record 16 channels of the DAQ-device. These channels correspond to the 16 BNC-connectors of the BNC Connector block and hence each channel corresponds to one sensor. If less sensors are connected or of interest, the corresponding channels can be removed from the recording code to reduce the size of the output file.

Secondly, the recording is not controlled by presentation of the odour but is only terminated once the animal removes its head fully from the odour port which hence allows for recordings of arbitrary length. This makes it possible to record all different phases of the behaviour run using one software alone. Recording only a fixed time interval can help to reduce the file size. Yet, users should be aware that this fixed time may not be equally suited for rewarded and unrewarded trials (or even the different pre-training stages).

Lastly, the sampling rate can be reduced from the current millisecond-precision if events of a larger timescale are of interest.

Part 4 – Schematics of custom made electronics modules

The following figures show the circuit diagrams for the custom made electronic modules. Please refer to the original publication, the accompanying data repository as well as the Bill of Materials for detailed instructions on the parts used. All circuit diagrams were constructed using the “Target 3001!” software (Ingenieurbüro Friedrich, Eichenzell, Germany).

Supplementary Figure 30 – Rotary Magnet Controller

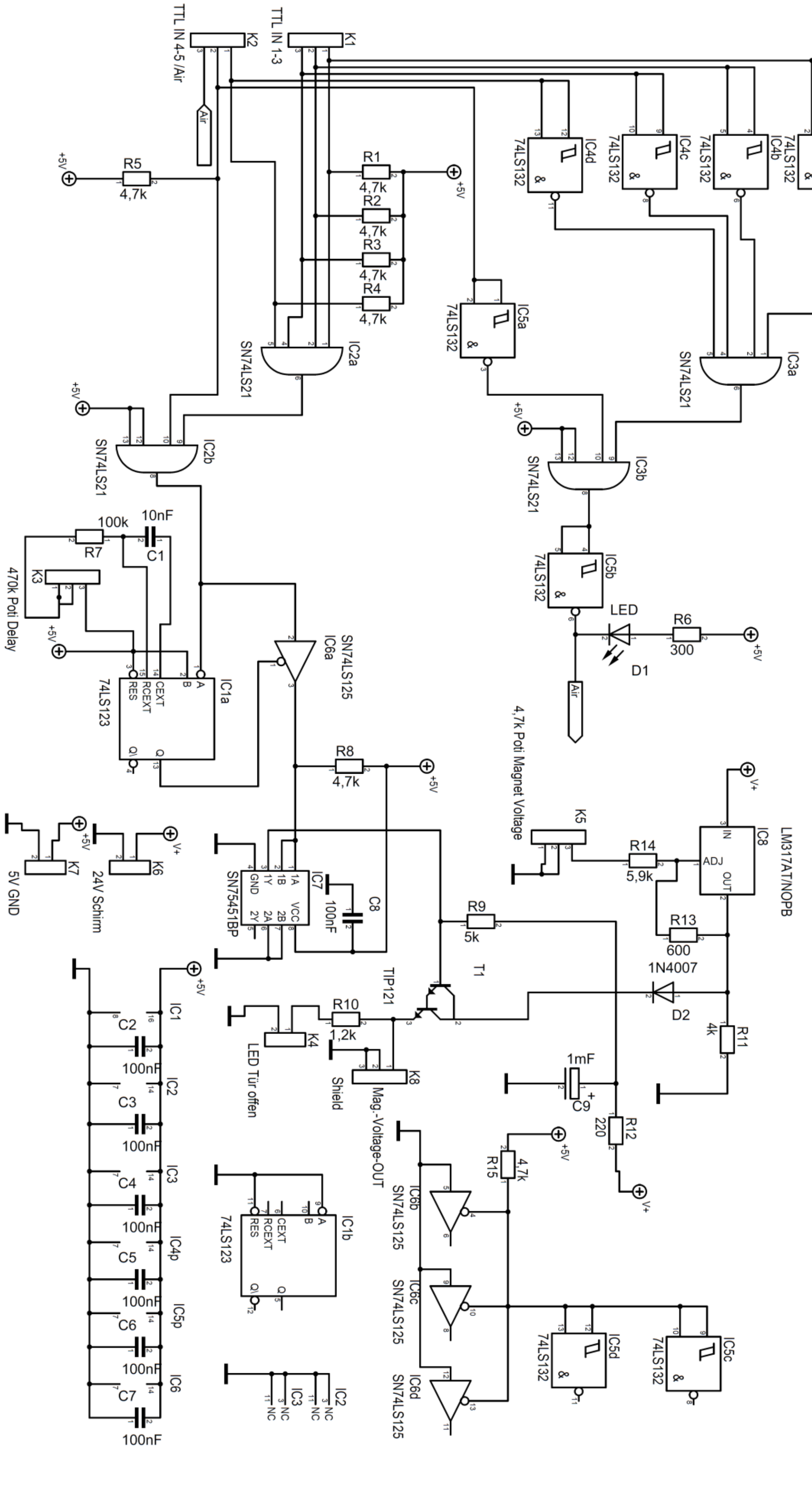

Supplementary Figure 31.1 – Gear Pump Controller Part 1

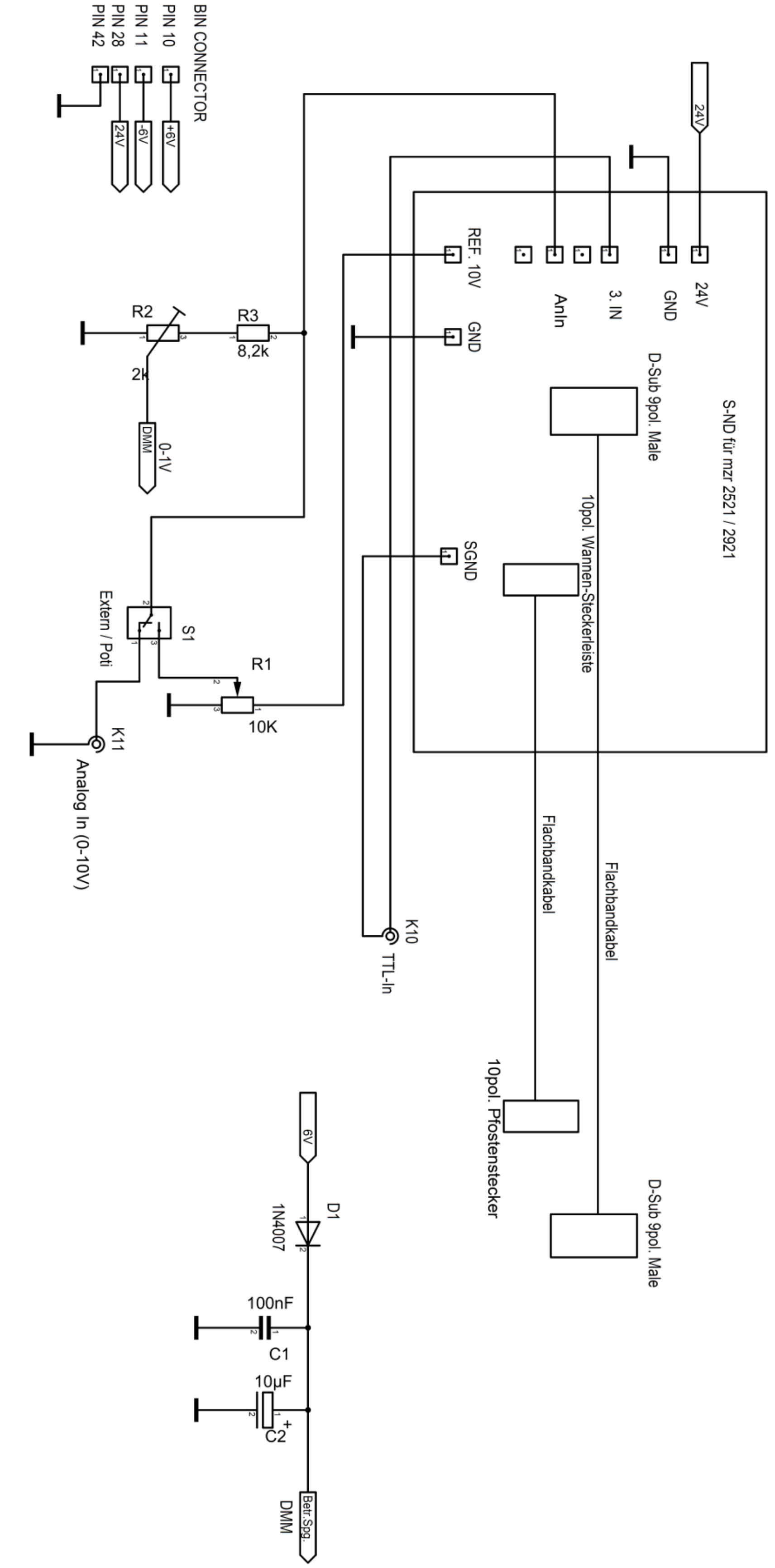

Supplementary Figure 31.2 – Gear Pump Controller Part 2

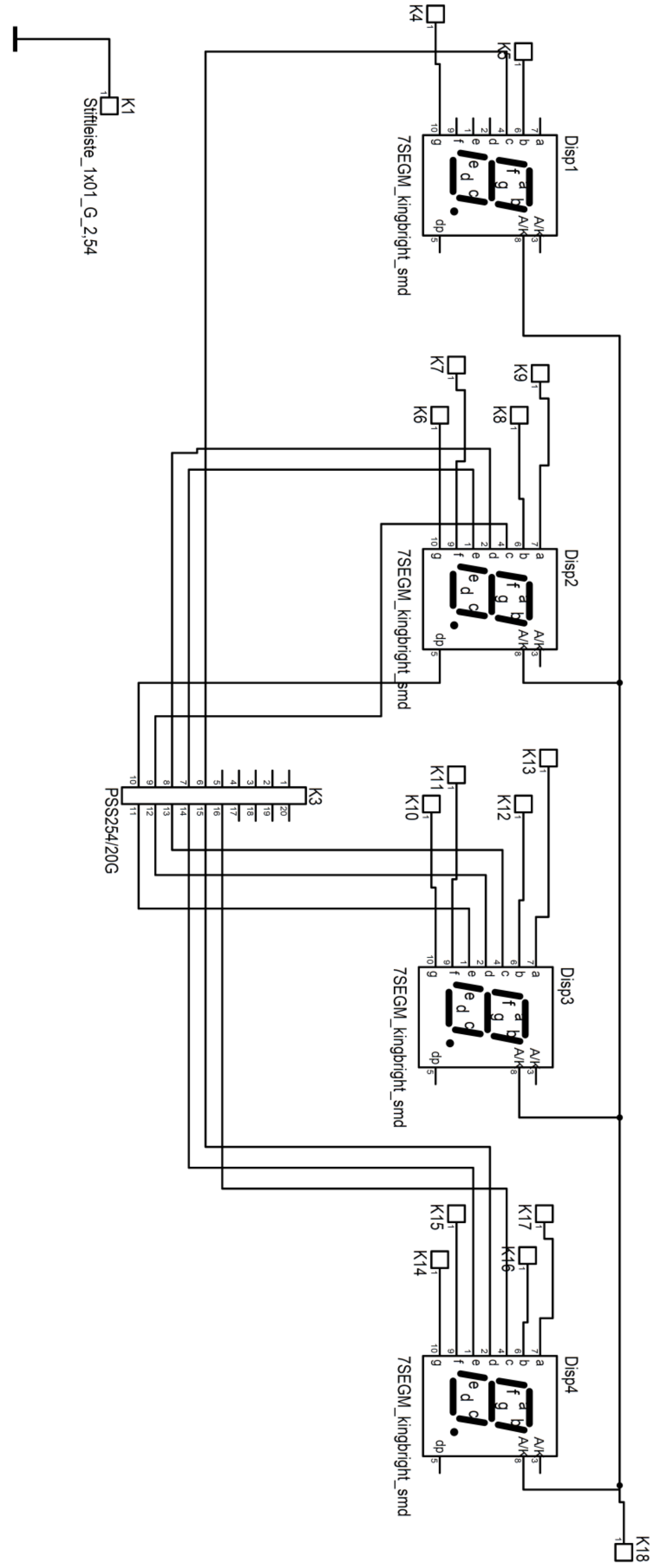

Supplementary Figure 31.3 – Gear Pump Controller Part 3

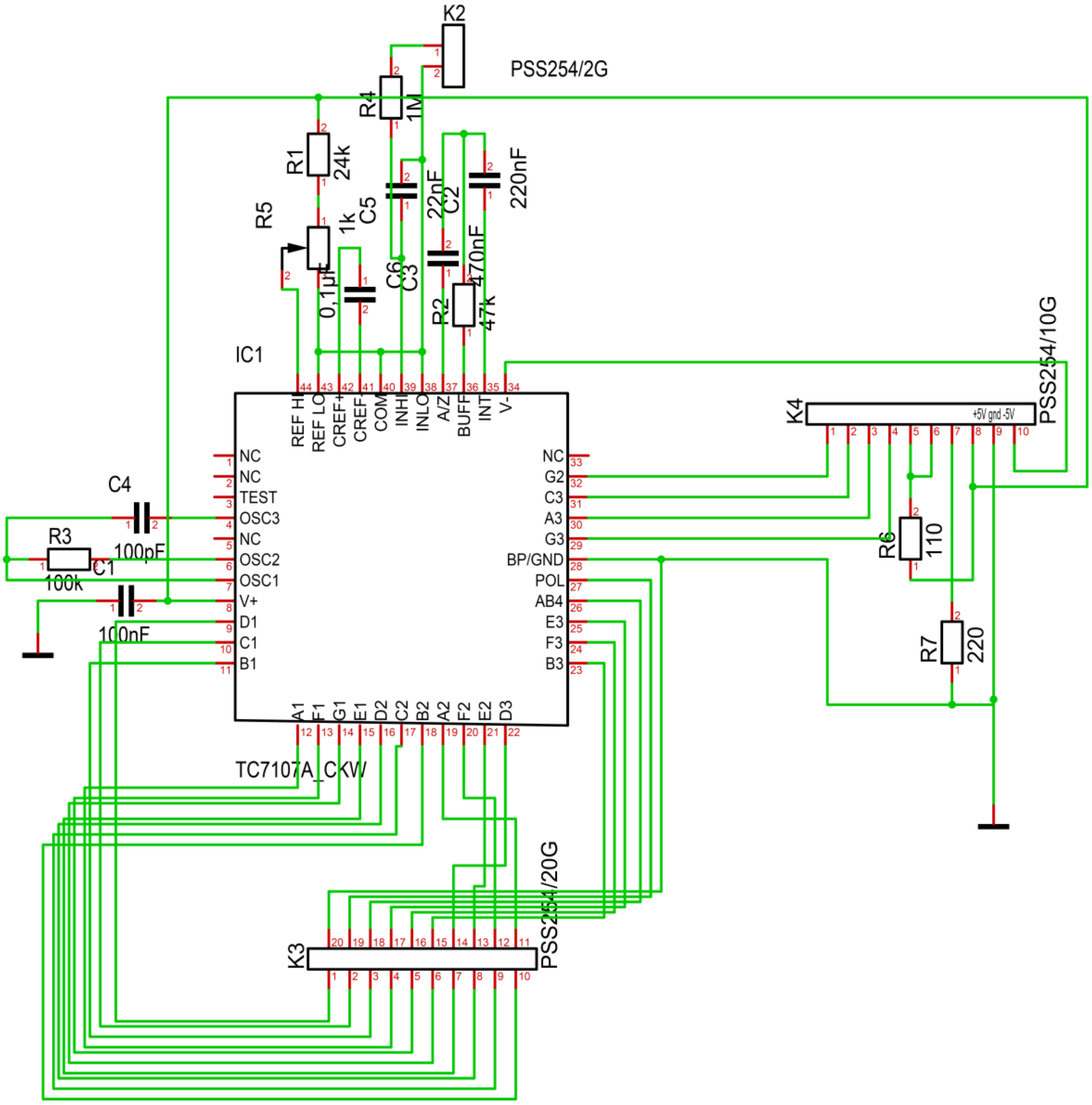

Supplementary Figure 32.1 – Valve Controller Part 1

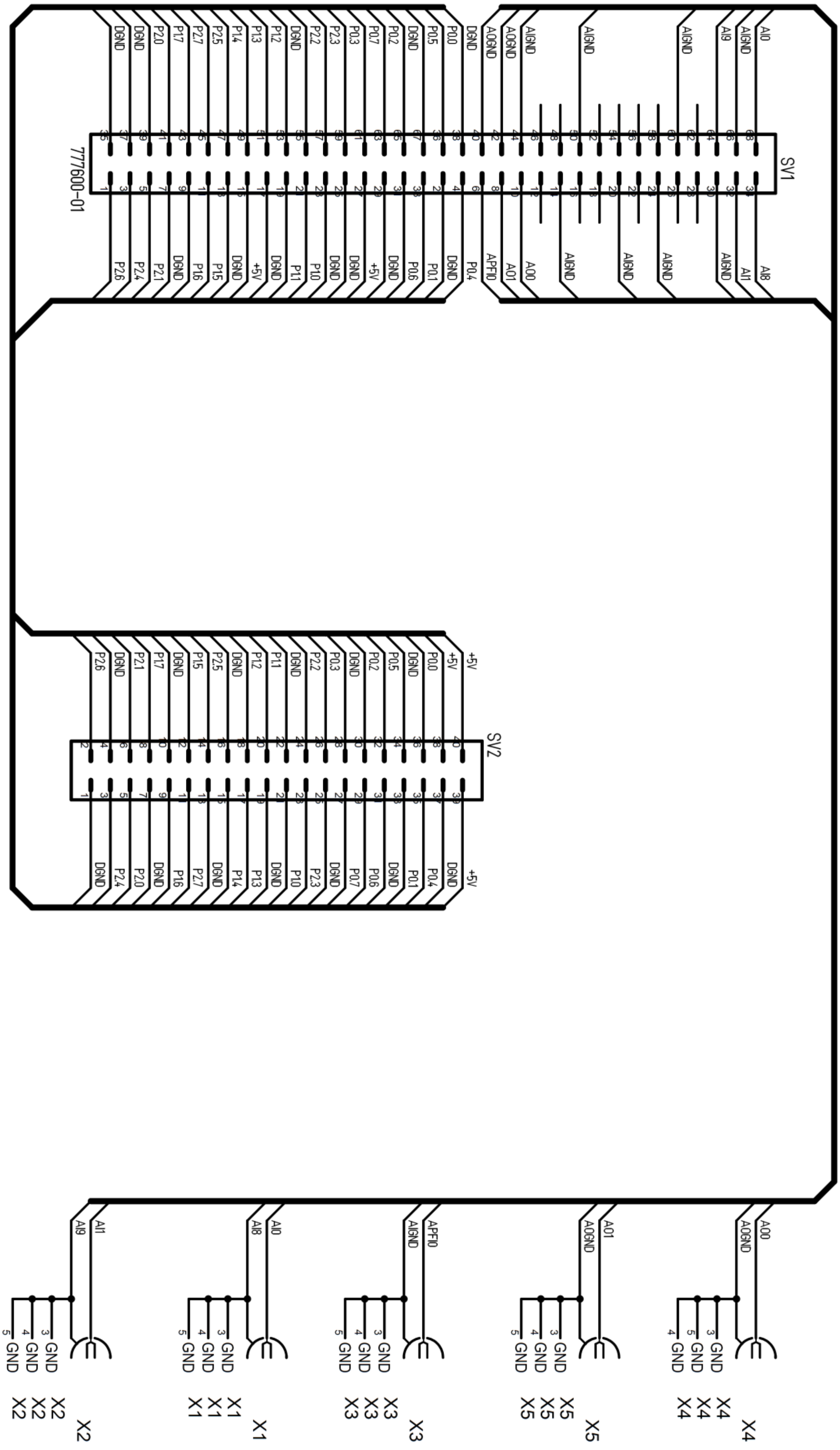

Supplementary Figure 32.2 – Valve Controller Part 2

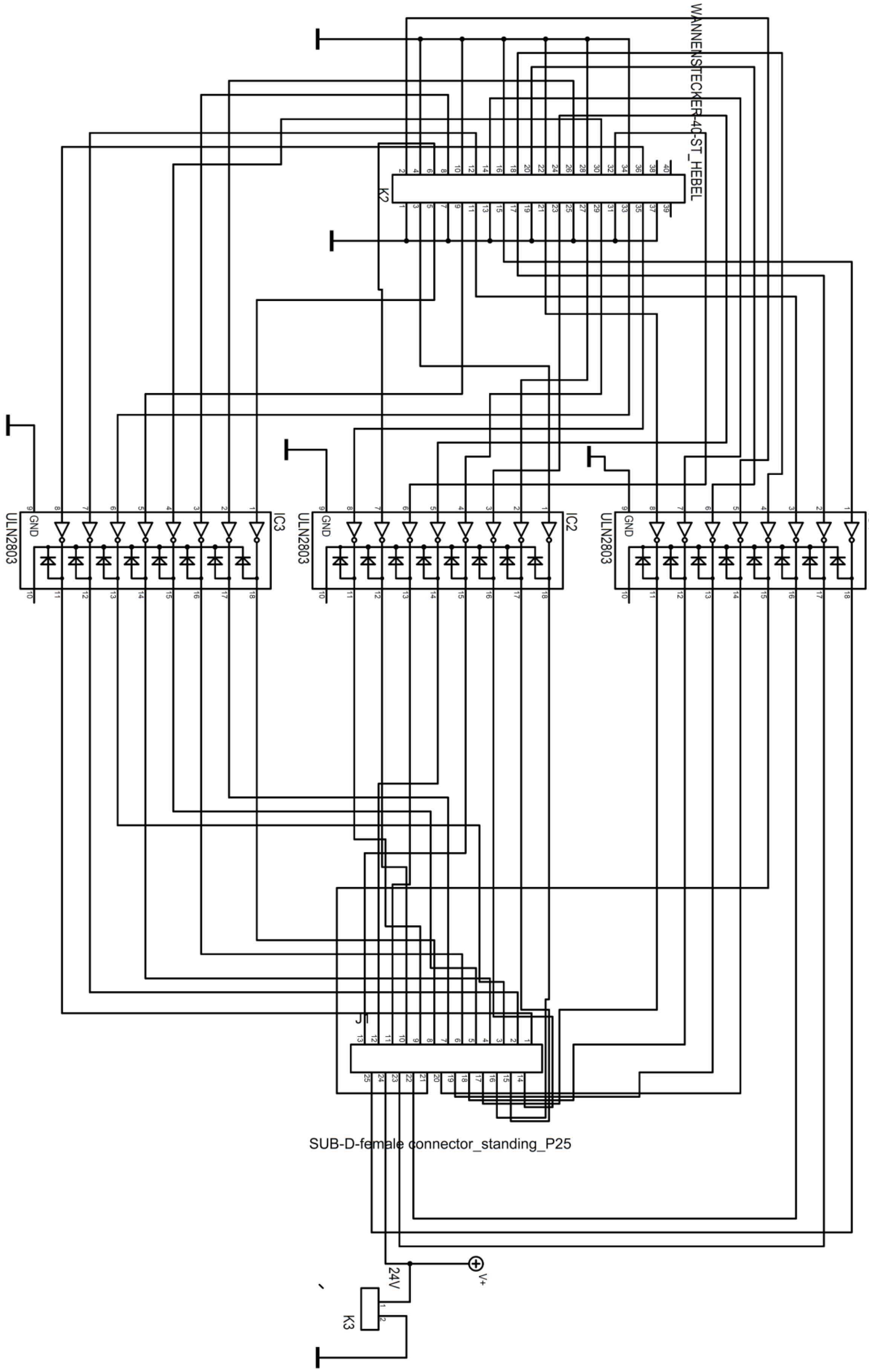

Supplementary Figure 32.3 – Valve Controller Part 3

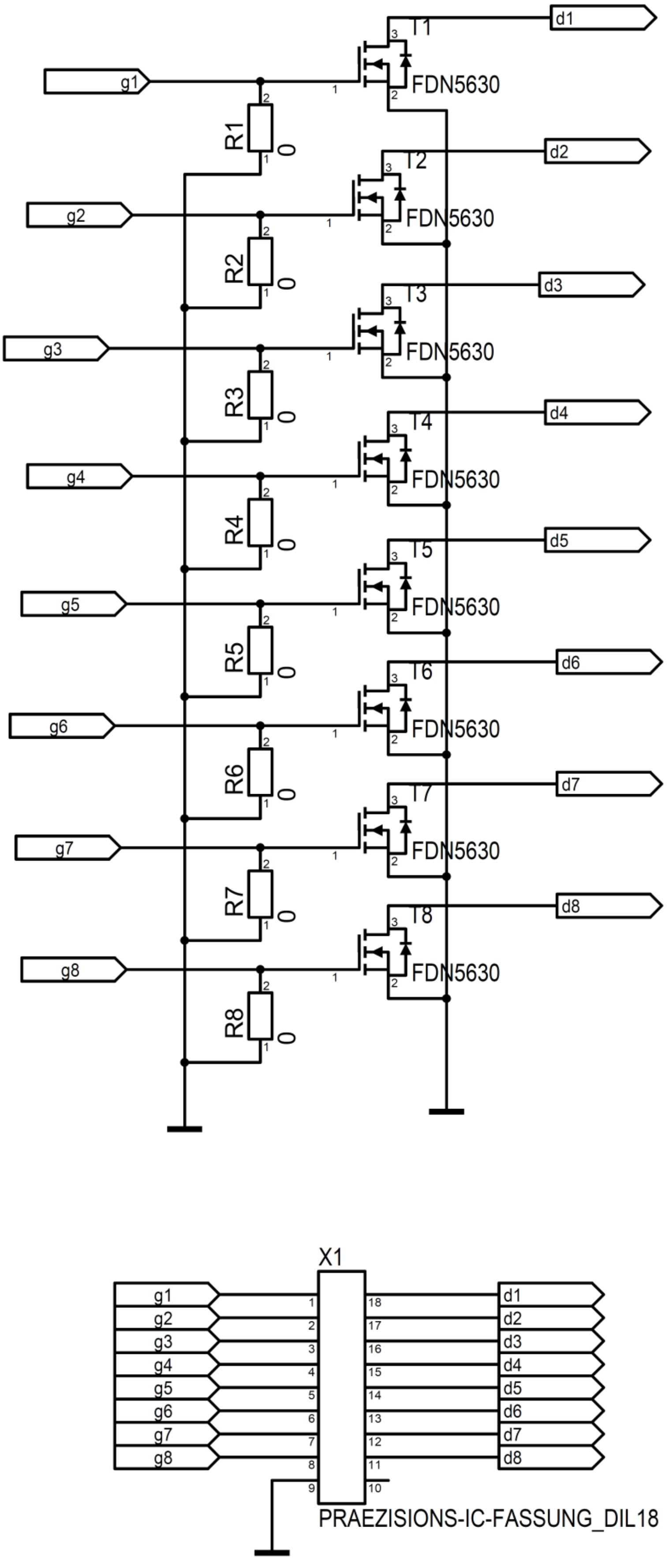

References

1. Vetter, R. S., Sage, A. E., Justus, K. A., Cardé, R. T. & Galizia, C. G. Temporal integrity of an airborne odor stimulus is greatly affected by physical aspects of the odor delivery system. *Chem. Senses* **31,** 359–69 (2006).

2. Khan, R. M. & Sobel, N. Neural processing at the speed of smell. *Neuron* **44,** 744–747 (2004).

3. Abraham, N. M. *et al.* Maintaining accuracy at the expense of speed: stimulus similarity defines odor discrimination time in mice. *Neuron* **44,** 865–76 (2004).

4. Kuner, T. & Schaefer, A. T. Molecules, cells and networks involved in processing olfactory stimuli in the mouse olfactory bulb. *e-Neuroforum* **2,** 61–67 (2011).

5. Uchida, N., Kepecs, A. & Mainen, Z. F. Seeing at a glance, smelling in a whiff: rapid forms of perceptual decision making. *Nat. Rev. Neurosci.* **7,** 485–91 (2006).

6. Bolaños, F., LeDue, J. M. & Murphy, T. H. Cost effective raspberry pi-based radio frequency identification tagging of mice suitable for automated in vivo imaging. *J. Neurosci. Methods* **276,** 79–83 (2017).

7. Shimshek, D. R. *et al.* Forebrain-specific glutamate receptor B deletion impairs spatial memory but not hippocampal field long-term potentiation. *J. Neurosci. Off. J. Soc. Neurosci.* **26,** 8428–8440 (2006).

8. Kepecs, A., Uchida, N. & Mainen, Z. F. Rapid and precise control of sniffing during olfactory discrimination in rats. *J. Neurophysiol.* **98,** 205–213 (2007).

9. Uchida, N. & Mainen, Z. F. Speed and accuracy of olfactory discrimination in the rat. *Nat. Neurosci.* **6,** 1224–1229 (2003).

10. Abraham, N. M. *et al.* Synaptic inhibition in the olfactory bulb accelerates odor discrimination in mice. *Neuron* **65,** 399–411 (2010).

11. Nunes, D. & Kuner, T. Disinhibition of olfactory bulb granule cells accelerates odour discrimination in mice. *Nat. Commun.* **6,** 8950 (2015).

12. Abraham, N. M., Guerin, D., Bhaukaurally, K. & Carleton, A. Similar odor discrimination behavior in head-restrained and freely moving mice. *PLoS One* **7,** e51789 (2012).

13. Shimshek, D. R. *et al.* Enhanced odor discrimination and impaired olfactory memory by spatially controlled switch of AMPA receptors. *PLoS Biol.* **3,** e354 (2005).

14. Strowbridge, B. W. Linking Local Circuit Inhibition to Olfactory Behavior: A Critical Role for Granule Cells in Olfactory Discrimination. *Neuron* **65,** 295–297 (2010).
